# Comparative safety signals of carboplatin and cisplatin in lung cancer: a pharmacovigilance study based on FAERS

**DOI:** 10.3389/fmed.2026.1846025

**Published:** 2026-07-22

**Authors:** Jiale Yang, Changbing Quan, Zixu Zhao, Yang Chen, Jincan Zhang, Rongbing Cai, Ge Sun

**Affiliations:** 1 Department of Thoracic Surgery, Second Affiliated Hospital of Dalian Medical University, Dalian, China; 2 Dalian Medical University, Dalian, China

**Keywords:** adverse events, carboplatin, cisplatin, FAERS, lung cancer, pharmacovigilance

## Abstract

**Background:**

Platinum-based chemotherapy remains a key component of lung cancer treatment. Although carboplatin and cisplatin are widely used, their real-world adverse-event reporting profiles may differ. This study compared carboplatin- and cisplatin-associated safety signals in lung cancer using the FDA Adverse Event Reporting System (FAERS), with emphasis on organ-specific signals, demographic heterogeneity, and time-to-onset (TTO) patterns.

**Methods:**

FAERS data from Q3 2005 to Q1 2026 were retrospectively analyzed. Reports were included when carboplatin or cisplatin was recorded as the primary suspect drug and lung cancer was listed as the indication. Disproportionality analyses were performed using reporting odds ratio (ROR), proportional reporting ratio (PRR), Bayesian Confidence Propagation Neural Network (BCPNN), and Multi-item Gamma Poisson Shrinker (MGPS). Positive Preferred Term (PT) signals were further screened for clinical relevance. TTO was summarized using median and interquartile range (IQR), and Weibull models were fitted to assess temporal failure patterns. Age- and sex-stratified analyses and exclusion-based sensitivity analyses were also conducted.

**Results:**

A total of 12,045 eligible case reports were included, comprising 10,159 carboplatin-associated and 1,886 cisplatin-associated reports. Carboplatin-related reports were mainly characterized by hematologic and infection-related signals, including anaemia, neutropenia, pancytopenia, thrombocytopenia, febrile neutropenia, leukopenia, and neutropenic sepsis, together with hepatobiliary and hypersensitivity-related signals. Cisplatin-related reports showed broader renal, vascular, auditory or vestibular, gastrointestinal, and electrolyte-related signals, including renal salt-wasting syndrome. Valid TTO information was available for 4,487 carboplatin-associated and 823 cisplatin-associated reports. Median TTO was 25 days for carboplatin (IQR, 8–67 days) and 21 days for cisplatin (IQR, 8–62 days). More than half of reports with valid TTO occurred within 0–30 days after treatment initiation: 55.0% for carboplatin and 59.7% for cisplatin. Weibull analysis supported an early-failure reporting pattern for both agents.

**Conclusion:**

Carboplatin and cisplatin showed distinct but overlapping adverse-event reporting profiles in lung cancer. These findings may support organ-specific and time-stratified safety monitoring, particularly during the first treatment cycle. However, they should be interpreted as pharmacovigilance signals rather than estimates of incidence, absolute risk, or causality, and require further prospective validation.

## 1 Introduction

Lung cancer is the leading cause of cancer-related mortality worldwide (1). Platinum-based chemotherapy, comprising cisplatin and carboplatin, remains the standard first-line treatment for patients with lung cancer (2, 3).

Cisplatin and carboplatin both exert antitumor activity by forming platinum-DNA adducts that disrupt DNA replication and transcription, yet their biochemical activation kinetics differ substantially (4, 5). Cisplatin undergoes relatively rapid aquation through its chloride leaving groups, whereas carboplatin contains a more stable bidentate 1,1-cyclobutanedicarboxylate leaving group that slows aquation and DNA-binding kinetics (4, 6). The resulting DNA platination rate of carboplatin has been reported to be approximately one to two orders of magnitude lower than that of cisplatin (4, 7). These kinetic differences help explain the lower nephrotoxic potential of carboplatin, while its systemic exposure profile is clinically associated with greater myelosuppression, including thrombocytopenia, neutropenia, and anaemia (8, 9).

The selection between these two agents is therefore not straightforward and requires careful consideration of multiple patient-specific factors (10). Clinicians must consider various factors, including patient age, renal function, and performance status (11). However, most clinical trials have historically excluded older patients and those with impaired renal function; consequently, these specific populations represent the very patients who most commonly receive these agents in routine clinical practice (10). This evidence-practice gap has resulted in a paucity of real-world safety data for these underrepresented populations, potentially compromising the ability to make truly evidence-based treatment decisions (12).

The FDA Adverse Event Reporting System (FAERS) offers a valuable means to bridge this evidence-practice gap by capturing safety reports from diverse real-world clinical settings (13). Unfortunately, most prior FAERS analyses have either aggregated data across all cancer types or treated cisplatin and carboplatin as therapeutically interchangeable (14). This approach fails to capture important toxicity differences between these two agents (14). Additionally, the temporal dynamics of these toxicities—specifically, whether they manifest early in the treatment course or accumulate progressively over time—remain sparsely investigated (15).

In the present study, we leveraged FAERS data spanning from Q3 2005 to Q1 2026 to characterize and compare adverse-event reporting profiles associated with carboplatin and cisplatin specifically in lung cancer reports. We applied four standard disproportionality signal detection methods to identify adverse events at both the System Organ Class (SOC) and Preferred Term (PT) levels. We examined the time-to-onset of these adverse events, evaluated TTO distributions, fitted Weibull models to characterize temporal failure patterns, conducted sensitivity analyses excluding selected co-reported chemotherapy agents, and performed age- and sex-stratified subgroup analyses (15). Through this comprehensive analytical approach, we aimed to delineate distinct adverse-event reporting profiles for these two platinum agents and to inform pharmacovigilance-oriented monitoring in clinical practice (16).

## 2 Materials and methods

### 2.1 Data collection and processing

**Figure 1:**
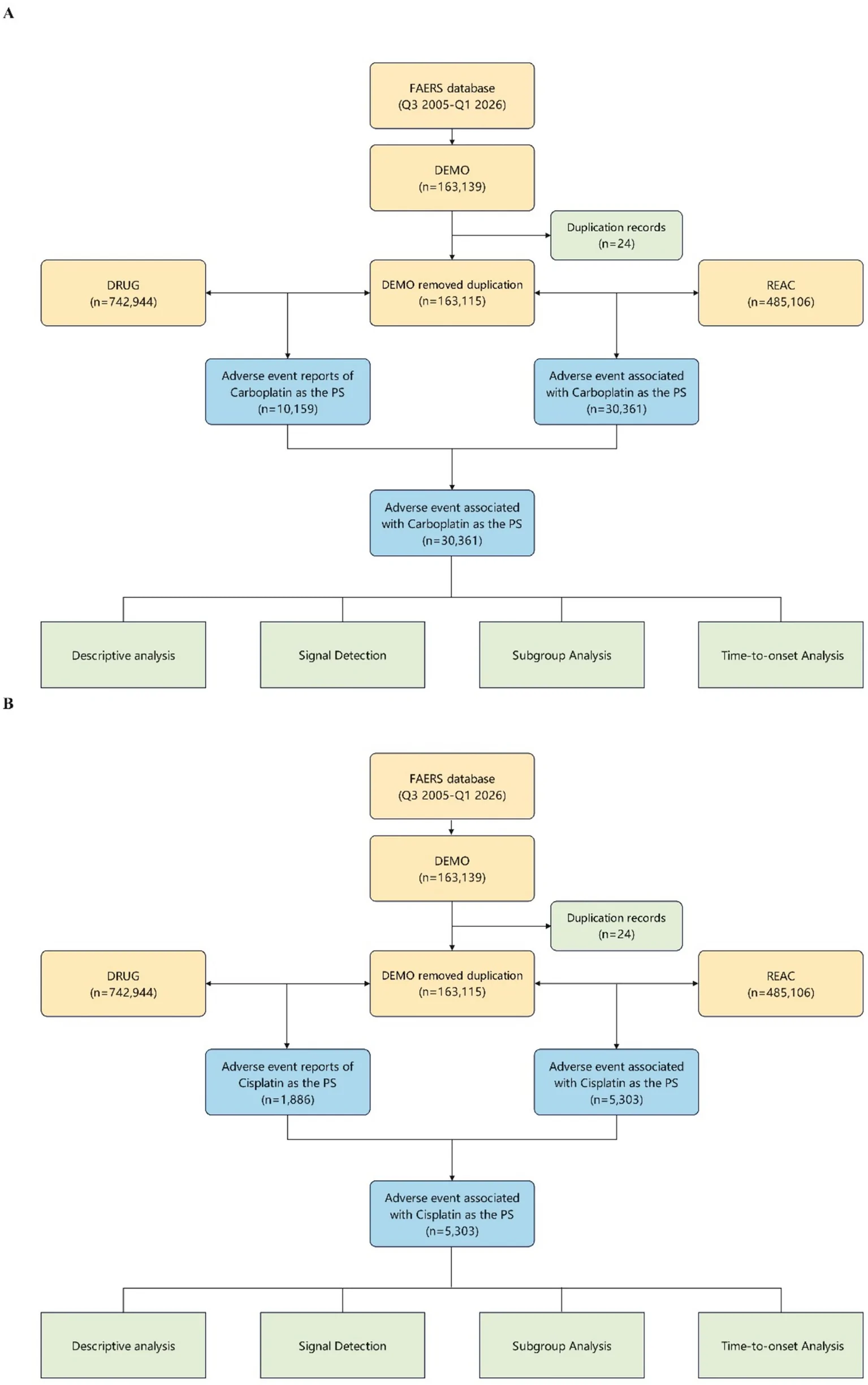
Study workflow for cohort selection and analysis of carboplatin and cisplatin reports from the FAERS database. **(A)** Workflow for identifying adverse event reports in which carboplatin was recorded as the primary suspect drug. **(B)** Workflow for identifying adverse event reports in which cisplatin was recorded as the primary suspect drug. DEMO, demographic file; DRUG, drug file; REAC, reaction file; PS, primary suspect drug; FAERS, FDA Adverse Event Reporting System.

This study leveraged data from the FAERS, a publicly available pharmacovigilance database maintained by the U.S. Food and Drug Administration that compiles spontaneous adverse event reports from healthcare professionals, consumers, and pharmaceutical manufacturers (17). Individual quarterly data extracts, spanning from Q3 2005 to Q1 2026, were downloaded from the FDA website. Following FDA-recommended standardized procedures, duplicate reports were identified and removed by evaluating unique case identifiers (CASEID), FDA receipt dates (FDA_DT), and primary report identifiers (PRIMARYID), with only the most recent and complete record retained for each case (16). This deduplication process is essential to prevent artificial inflation of signal strength that could result from multiple reports of the same adverse event (18). Adverse events were subsequently standardized and coded according to MedDRA (Medical Dictionary for Regulatory Activities) version 26.1, at both the PT and SOC levels (19). Reports were included if they met the following criteria: (i) designation of carboplatin or cisplatin as the primary suspect drug (PS); (ii) an indication of lung cancer (including non-small cell lung cancer (NSCLC), small cell lung cancer (SCLC), and unspecified lung malignancies); and (iii) availability of demographic data for baseline characterization. A detailed schematic overview of the study workflow is presented in Figure 1.

### 2.2 Statistical analysis

Four established disproportionality analysis algorithms—the Reporting Odds Ratio (ROR), the Proportional Reporting Ratio (PRR), the Bayesian Confidence Propagation Neural Network (BCPNN), and the Multi-item Gamma Poisson Shrinker (MGPS)—were applied to detect potential safety signals (20). Detailed formulas for these algorithms are provided in the Supplementary materials (Supplementary Table S1 (2 × 2 Contingency Table for Disproportionality Analysis) and Supplementary Table S2 (Disproportionality Analysis Algorithms and Signal Criteria)).

A positive disproportionality signal was defined by simultaneously meeting the pre-specified positive thresholds of all four methods: a ROR with the lower bound of its 95% confidence interval (CI) exceeding 1, based on at least 3 reported cases; a PRR ≥ 2 with a corresponding Yates-corrected chi-square (χ^2^) ≥ 4, based on at least 3 cases; a BCPNN with the lower bound of the 95% CI for the Information Component (IC025) > 0; and an MGPS with the lower bound of the 95% CI for the Empirical Bayes Geometric Mean (EBGM05) > 2 (21). This conservative definition prioritized specificity over sensitivity and was used to reduce false-positive signal detection (22). Because the disproportionality analyses were intended for exploratory signal detection rather than confirmatory hypothesis testing, formal multiplicity adjustment was not applied (23). To reduce the likelihood of false-positive findings, PT-level signals were required to meet the predefined positive criteria of all four algorithms and were further subjected to clinical relevance filtering before final interpretation (24). Detailed contingency table definitions and computational formulas are provided in the Supplementary materials (Supplementary Tables S1, S2).

After algorithmic signal detection, all positive PTs were further screened for clinical relevance (25). PTs were retained only if they represented clinically interpretable adverse events with plausible pharmacological or toxicological relevance to platinum exposure (26). PTs primarily reflecting disease progression, tumor status, treatment failure, drug-use patterns, medication errors, product-use issues, device or manufacturing problems, procedures, administrative outcomes, or nonspecific reporting circumstances were excluded from the final clinically relevant signal set. This filtering step was applied after disproportionality testing and before final PT-level tabulation, subgroup analyses, sensitivity analyses, and interpretation.

Time-to-onset (TTO) was defined as the interval, in days, between the reported therapy start date (START_DT) and the adverse event onset date (EVENT_DT). The overall TTO analysis was conducted at the deduplicated case-report level. For each platinum agent, each eligible report contributed one TTO value. Reports with multiple PTs were counted once in the overall TTO analysis to avoid over-weighting reports containing multiple adverse events. Reports were included in the TTO analysis only when both START_DT and EVENT_DT were available and chronologically valid. Reports with missing, incomplete, or implausible date information were excluded from the TTO analysis. The number and proportion of reports included in the TTO analysis among all eligible reports were reported in the Results section.

TTO was summarized using the median and interquartile range (IQR), together with the observed minimum and maximum values. To characterize the temporal distribution of adverse event onset, TTO was categorized into prespecified intervals: 0–30, 31–60, 61–90, 91–120, 121–150, 151–180, 181–360, and >360 days. The proportions of reports within each interval were calculated and visualized separately for carboplatin and cisplatin among reports with available TTO data. Because TTO analyses were restricted to reports with complete and valid date information, the findings were interpreted as descriptive temporal reporting patterns rather than incidence-based estimates of onset timing (27).

A Weibull distribution model was fitted to the TTO data to evaluate the temporal failure pattern of adverse events (28). The scale parameter (*α*), shape parameter (*β*), and corresponding 95% confidence intervals were estimated. Failure patterns were classified according to the *β* estimate and its 95% confidence interval: early failure was defined as *β* < 1 with an upper 95% confidence limit below 1; random failure was defined when the 95% confidence interval included 1; and wear-out failure was defined as *β* > 1 with a lower 95% confidence limit above 1 (29).

Exclusion-based sensitivity analyses were conducted to evaluate the robustness of the primary disproportionality findings after separately excluding cases co-reported with selected chemotherapy agents, including pemetrexed, paclitaxel, and albumin-bound paclitaxel, as applicable (30).

Age- and sex-based subgroup analyses were first performed at the PT level to explore demographic heterogeneity in reporting patterns. These analyses compared PT-level reporting odds between patients aged <65 years and those aged ≥65 years, and between male and female patients. PTs detected in both comparison strata were included in the comparative subgroup analyses, whereas PTs detected only in one stratum were presented as stratum-specific signals without comparative ROR estimates.

To further assess whether the primary PT-level signals were potentially driven by imbalance in age or sex distribution, additional age- and sex-restricted sensitivity analyses were subsequently performed. In these analyses, PT-level disproportionality analyses were repeated separately among patients aged <65 years, patients aged ≥65 years, male patients, and female patients. These restricted sensitivity analyses were used to examine whether selected positive signals persisted after limiting the analysis to specific demographic strata (16). However, both the subgroup analyses and the restricted sensitivity analyses were interpreted descriptively because stratification by age or sex alone cannot fully account for treatment selection in real-world thoracic oncology, which is also influenced by performance status, renal function, comorbidity burden, frailty, baseline marrow reserve, dose intensity, and supportive care. All data processing, statistical analyses, and visualizations were performed using R software.

## 3 Results

### 3.1 Descriptive analysis

The analysis included 10,159 FAERS case reports in which carboplatin was listed as the primary suspect drug, corresponding to 30,361 PT-level adverse-event records, and 1,886 FAERS case reports in which cisplatin was listed as the primary suspect drug, corresponding to 5,303 PT-level adverse-event records (Figure 1). Among all carboplatin-associated reports, those aged ≥65 years constituted the largest proportion in the carboplatin group (47.9%), followed by patients aged 18–64 years (35.0%). Conversely, in the cisplatin group, patients aged 18–64 years represented the majority (50.6%), whereas those aged ≥65 years comprised 33.0%. Regarding patient sex, adverse events in the cisplatin group were reported for 55.2% males and 33.4% females. The carboplatin group demonstrated a similar distribution, with 53.5% males and 33.8% females. Notably, body weight data were missing in over 60% of reports for both agents. The primary reporting countries for carboplatin were France (18.8%), the United States (14.3%), and Germany (12.1%), whereas for cisplatin, the primary reporting countries were the United States (22.5%), France (13.8%), and Italy (13.0%) (Table 1).

**Table 1:** Clinical characteristics of treatment-related adverse events (AEs).

Characteristics	Carboplatin N (proportion)	Cisplatin N (proportion)
Age		
Age <18 years	8 (0.1%)	4 (0.2%)
18 ≤ age < 65 years	3,555 (35.0%)	954 (50.6%)
65 ≤ age < 85 years	4,828 (47.5%)	621 (32.9%)
Age ≥ 85 years	40 (0.4%)	1 (0.1%)
Unknown	1,728 (17.0%)	306 (16.2%)
Sex		
Male	5,440 (53.5%)	1,041 (55.2%)
Female	3,434 (33.8%)	630 (33.4%)
Unknown	1,285 (12.6%)	215 (11.4%)
Weight		
< 50 kg	293 (2.9%)	44 (2.3%)
50 kg ≤ weight ≤ 100 kg	3,374 (33.2%)	627 (33.2%)
> 100 kg	197 (1.9%)	28 (1.5%)
Unknown	6,295 (62.0%)	1,187 (62.9%)
Reporting countries (top5)		
	FR (1,913, 18.8%)	US (425, 22.5%)
	US (1,455, 14.3%)	FR (261, 13.8%)
	DE (1,230, 12.1%)	IT (246, 13.0%)
	JP (949, 9.3%)	JP (145, 7.7%)
	IT (786, 7.7%)	DE (132, 7.0%)

**Figure 2:**
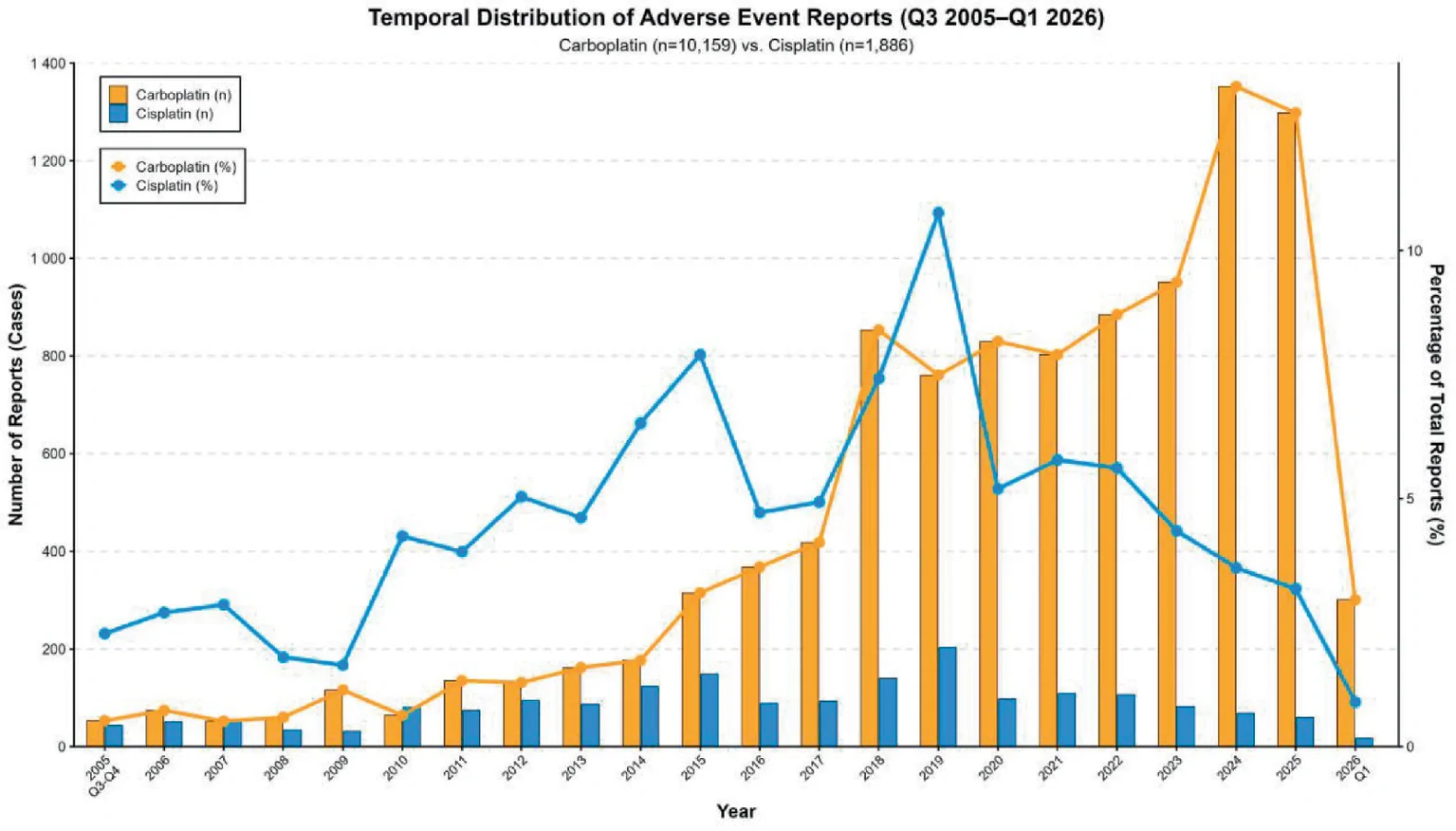
Temporal distribution of adverse event reports for cisplatin and carboplatin in FAERS (Q3 2005–Q1 2026).

The temporal distribution of adverse event reports was analyzed by calendar year. The proportion of AEs associated with cisplatin peaked in 2019 at 10.8% and subsequently declined steadily, falling to 3.2% by 2025. In contrast, carboplatin exhibited the opposite trend, with a consistent increase throughout the study period and a marked acceleration after 2017, reaching a peak of 13.3% in 2024 (Figure 2).

### 3.2 System organ class (SOC) distribution

**Figure 3:**
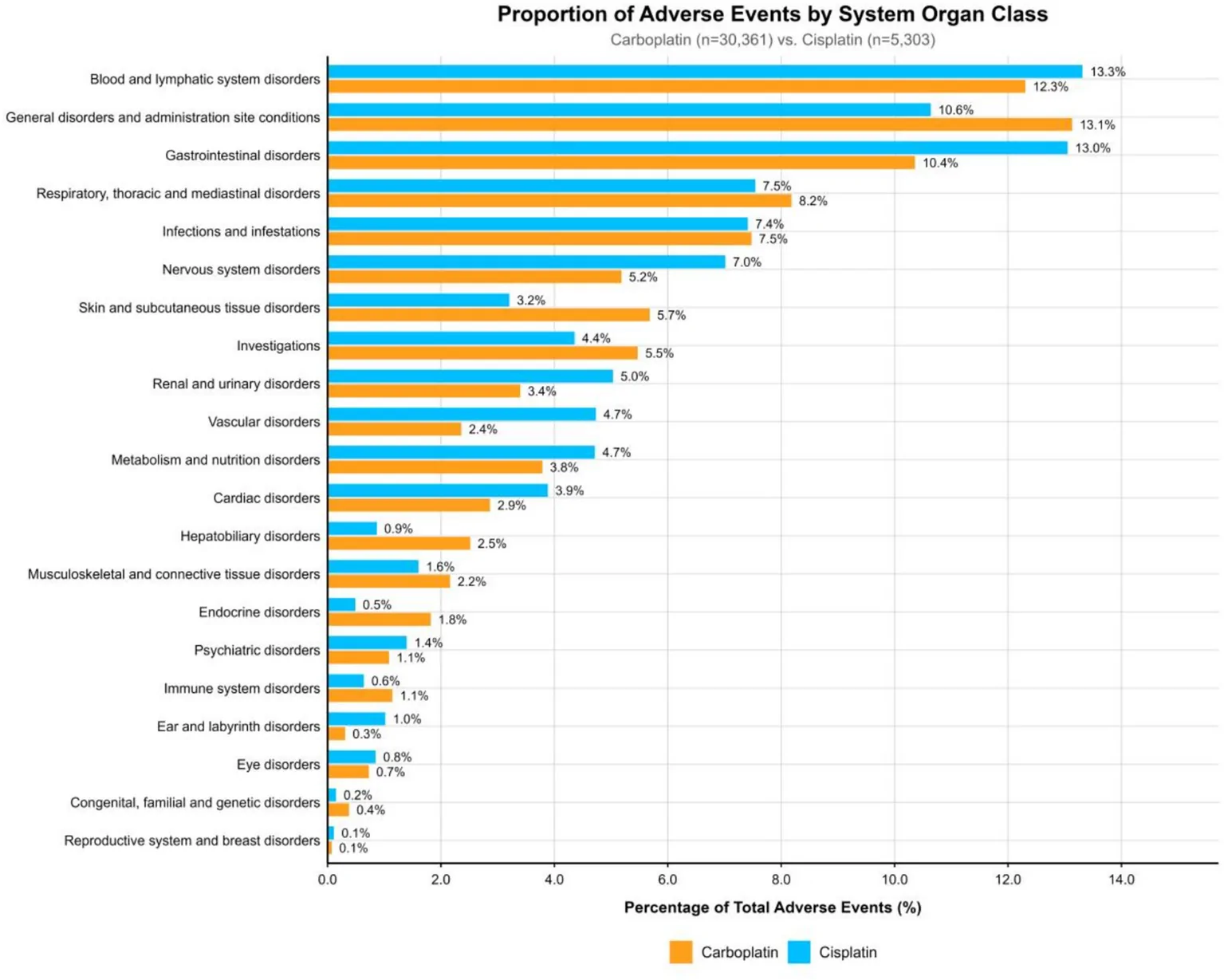
Distribution of adverse events by system organ class for carboplatin and cisplatin in the FAERS database.

After mapping PT-level adverse-event records to SOC categories and excluding categories less directly related to adverse drug events, such as product issues, social circumstances, injuries, poisoning, and procedural complications, 21 SOC categories remained for descriptive distribution analysis. The distribution of reported cases for each agent across these SOCs is shown in Figure 3. For carboplatin, the largest SOC proportions were general disorders and administration site conditions, followed by blood and lymphatic system disorders, gastrointestinal disorders, and respiratory, thoracic and mediastinal disorders (Figure 3). A broadly similar pattern was observed for cisplatin, with blood and lymphatic system disorders and gastrointestinal disorders accounting for the largest proportions, followed by general disorders and administration site conditions, while respiratory, thoracic and mediastinal disorders were also among the frequently reported SOCs (Figure 3). The comprehensive disproportionality analysis results of all 21 SOCs (including ROR, PRR, EBGM and IC values) are detailed in the Supplementary materials (Supplementary Table S3: Disproportionality analysis of System Organ Classes (SOCs) related to carboplatin in the FAERS database, Supplementary Table S4: Disproportionality analysis of System Organ Classes (SOCs) related to cisplatin in the FAERS database).

A total of 12 SOCs met the predefined positivity criteria across all four algorithms (ROR, PRR, BCPNN, and MGPS) (Table 1). Among these, 3 SOCs were reported by both drugs, 3 SOCs were specific to carboplatin, and 6 SOCs were specific to cisplatin. Among the SOCs reported by both drugs, blood and lymphatic system disorders (3,736 cases for carboplatin vs. 706 cases for cisplatin) and infections and infestations (2,270 cases vs. 393 cases) both accounted for relatively high reporting proportions for both drugs, and direct comparison showed no clear difference between carboplatin and cisplatin for blood and lymphatic system disorders (ROR = 0.91, 95% CI: 0.84–1.00) or infections and infestations (ROR = 1.01, 95% CI: 0.90–1.13), suggesting that the frequency of adverse event reports for these two systems was generally similar for carboplatin and cisplatin. It is notable that the reporting of renal and urinary disorders was significantly lower for carboplatin (ROR = 0.66, 95% CI: 0.58–0.76), indicating that nephrotoxicity-related reports were significantly fewer for carboplatin than for cisplatin, which is consistent with the clinical feature of relatively mild nephrotoxicity of carboplatin.

For drug-specific SOCs (Table 2), carboplatin showed hepatobiliary disorders (764 cases), endocrine disorders (552 cases), and immune system disorders (347 cases), suggesting that carboplatin may have these system-specific adverse event signals. In contrast, the specific SOCs of cisplatin are more extensive, including gastrointestinal disorders (692 cases), nervous system disorders (372 cases), vascular disorders (251 cases), metabolism and nutrition disorders (250 cases), cardiac disorders (206 cases), and ear and labyrinth disorders (54 cases). This distribution is highly consistent with the known toxicity profile of cisplatin, and its gastrointestinal toxicity, ototoxicity, neurotoxicity, and cardiovascular toxicity have been widely recognized in clinical practice.

**Table 2:** Disproportionality analysis of System Organ Classes (SOCs) between carboplatin and cisplatin.

SOC	Carboplatin (N)	Cisplatin (N)	ROR (95% CI)
Both drugs reported			
Blood and lymphatic system disorders	3,736	706	0.91 (0.84–1.00)
Infections and infestations	2,270	393	1.01 (0.90–1.13)
Renal and urinary disorders	1,032	267	0.66 (0.58–0.76)
Carboplatin-specific			
Hepatobiliary disorders	764	–	–
Endocrine disorders	552	–	–
Immune system disorders	347	–	–
Cisplatin-specific			
Gastrointestinal disorders	–	692	–
Nervous system disorders	–	372	–
Vascular disorders	–	251	–
Metabolism and nutrition disorders	–	250	–
Cardiac disorders	–	206	–
Ear and labyrinth disorders	–	54	–

ROR values represent the direct comparison of reporting odds ratios between carboplatin and cisplatin; ROR < 1 indicates a lower reporting odds ratio for carboplatin relative to cisplatin; ROR > 1 indicates a higher reporting odds ratio for carboplatin relative to cisplatin. The 95% confidence intervals that do not cross 1.0 indicate statistically significant differences.

Overall, carboplatin and cisplatin showed different safety characteristics in their SOC distributions. The adverse events associated with cisplatin involved a wider range of organ systems, especially prominent gastrointestinal, neurological, and ototoxicity-related signals. In contrast, reports of nephrotoxicity associated with carboplatin were relatively low, whereas its specific signals in the liver, endocrine, and immune systems warrant attention. These findings provide a basis for individualized safety monitoring and clinical risk awareness for the two platinum agents in lung cancer treatment.

### 3.3 Top 20 preferred terms analysis and comparative analysis of adverse events

Analysis of the 20 most frequently reported PTs identified distinct safety profiles for each platinum agent (Tables 3, 4). Carboplatin demonstrated a predominant signal profile characterized by bone marrow suppression. Significant disproportionality signals were detected for anaemia (ROR = 2.43, 95% CI: 2.23–2.65; IC = 1.14), neutropenia (ROR = 3.10, 95% CI: 2.83–3.40; IC = 1.44), and pancytopenia (ROR = 6.42, 95% CI: 5.80–7.11; IC = 2.25). A particularly notable signal was observed for neutropenic sepsis (ROR = 7.22, 95% CI: 5.93–8.80; IC = 2.37), underscoring the severity of infectious complications associated with myelosuppression. Beyond hematologic toxicities, hepatocellular injury emerged as a significant concern, with hepatic cytolysis (ROR = 3.40, 95% CI: 2.69–4.31; IC = 1.56), hypertransaminasaemia (ROR = 5.22, 95% CI: 3.92–6.96; IC = 2.04), and hepatocellular injury (ROR = 3.43, 95% CI: 2.57–4.58; IC = 1.57) demonstrating substantial disproportionality. Additionally, hypersensitivity reactions, particularly anaphylactic shock (ROR = 4.71, 95% CI: 3.67–6.05; IC = 1.63), constituted significant safety signals.

**Table 3:** Top 20 most frequently reported preferred terms associated with carboplatin in the FAERS database.

Preferred term (PT)	*N*	ROR (95% CI)	PRR (*χ*^2^)	EBGM (EBGM05)	IC (IC025)
Anaemia	627	2.43 (2.23–2.65)	2.40 (445.50)	2.21 (2.03)	1.14 (1.01)
Neutropenia	556	3.10 (2.83–3.40)	3.06 (644.81)	2.71 (2.47)	1.44 (1.30)
Pancytopenia	529	6.42 (5.80–7.11)	6.33 (1674.58)	4.75 (4.28)	2.25 (2.09)
Thrombocytopenia	508	3.12 (2.83–3.44)	3.09 (597.41)	2.73 (2.48)	1.45 (1.30)
Febrile neutropenia	470	2.96 (2.68–3.28)	2.93 (504.14)	2.62 (2.37)	1.39 (1.24)
Acute kidney injury	395	2.89 (2.59–3.22)	2.86 (403.86)	2.56 (2.30)	1.36 (1.19)
Leukopenia	349	4.85 (4.30–5.48)	4.81 (799.36)	3.88 (3.44)	1.96 (1.77)
Septic shock	167	3.67 (3.09–4.34)	3.65 (258.97)	3.13 (2.64)	1.65 (1.38)
Mucosal inflammation	157	2.96 (2.50–3.52)	2.95 (169.85)	2.63 (2.22)	1.40 (1.13)
Neutropenic sepsis	146	7.22 (5.93–8.80)	7.19 (526.43)	5.18 (4.25)	2.37 (2.06)
Polyneuropathy	110	8.89 (7.02–11.25)	8.86 (482.13)	5.94 (4.69)	2.57 (2.18)
Hyperthyroidism	93	2.91 (2.33–3.63)	2.90 (97.23)	2.59 (2.08)	1.37 (1.03)
Hepatic cytolysis	85	3.40 (2.69–4.31)	3.39 (117.19)	2.95 (2.33)	1.56 (1.19)
Rash maculo-papular	85	3.58 (2.83–4.54)	3.58 (127.46)	3.08 (2.43)	1.62 (1.24)
Agranulocytosis	83	4.70 (3.67–6.02)	4.69 (183.70)	3.81 (2.98)	1.93 (1.53)
Anaphylactic shock	81	4.71 (3.67–6.05)	4.70 (179.83)	3.82 (2.97)	1.93 (1.52)
Hypomagnesaemia	80	3.59 (2.82–4.59)	3.59 (120.55)	3.09 (2.42)	1.63 (1.24)
Febrile bone marrow aplasia	66	6.52 (4.88–8.70)	6.50 (214.42)	4.84 (3.62)	2.27 (1.78)
Hypertransaminasaemia	63	5.22 (3.92–6.96)	5.21 (159.21)	4.13 (3.10)	2.04 (1.56)
Hepatocellular injury	57	3.43 (2.57–4.58)	3.43 (79.84)	2.98 (2.23)	1.57 (1.11)

**Table 4:** Top 20 most frequently reported preferred terms associated with cisplatin in the FAERS database.

Preferred term (PT)	*N*	ROR (95% CI)	PRR (*χ*^2^)	EBGM (EBGM05)	IC (IC025)
Neutropenia	144	4.25 (3.59–5.03)	4.16 (332.53)	4.02 (3.39)	2.01 (1.73)
Febrile neutropenia	106	3.52 (2.89–4.28)	3.47 (180.65)	3.38 (2.78)	1.76 (1.44)
Acute kidney injury	98	3.80 (3.10–4.66)	3.75 (190.80)	3.64 (2.97)	1.86 (1.53)
Thrombocytopenia	83	2.62 (2.11–3.27)	2.60 (79.77)	2.55 (2.05)	1.35 (1.00)
Pancytopenia	73	3.91 (3.09–4.95)	3.87 (149.36)	3.75 (2.96)	1.91 (1.51)
Pulmonary embolism	65	3.08 (2.40–3.95)	3.06 (87.46)	2.99 (2.33)	1.58 (1.17)
Leukopenia	63	4.19 (3.25–5.40)	4.15 (144.54)	4.01 (3.11)	2.00 (1.57)
Renal failure	46	2.99 (2.23–4.02)	2.97 (58.57)	2.91 (2.17)	1.54 (1.05)
Septic shock	38	4.25 (3.06–5.89)	4.22 (89.49)	4.08 (2.94)	2.03 (1.44)
Neutropenic sepsis	33	7.20 (5.05–10.27)	7.16 (162.22)	6.71 (4.70)	2.75 (2.00)
Aortic thrombosis	29	75.37 (46.04–123.39)	74.97 (1157.58)	41.45 (25.32)	5.37 (3.49)
Oesophagitis	29	5.20 (3.57–7.56)	5.18 (92.50)	4.95 (3.40)	2.31 (1.58)
Nephropathy toxic	25	8.54 (5.66–12.88)	8.50 (151.40)	7.86 (5.21)	2.97 (2.04)
Acute myocardial infarction	24	7.05 (4.65–10.70)	7.03 (115.21)	6.59 (4.35)	2.72 (1.83)
Bone marrow failure	23	3.27 (2.15–4.96)	3.26 (34.76)	3.18 (2.09)	1.67 (0.94)
Multiple organ dysfunction syndrome	20	3.98 (2.54–6.23)	3.97 (42.58)	3.84 (2.45)	1.94 (1.11)
Renal tubular necrosis	19	9.48 (5.90–15.21)	9.45 (129.97)	8.65 (5.39)	3.11 (1.96)
Hypomagnesaemia	17	3.88 (2.39–6.32)	3.87 (34.79)	3.76 (2.31)	1.91 (1.00)
Renal salt-wasting syndrome	16	242.00 (94.66–618.69)	241.27 (1044.17)	66.53 (26.02)	6.06 (2.84)
Gastrointestinal toxicity	16	6.81 (4.10–11.33)	6.80 (73.61)	6.39 (3.84)	2.68 (1.55)

Cisplatin showed a different PT-level signal profile. Nephrotoxicity represented a principal safety concern, with acute kidney injury (ROR = 3.80, 95% CI: 3.10–4.66; IC = 1.86) and renal failure (ROR = 2.99, 95% CI: 2.23–4.02; IC = 1.54) demonstrating significant disproportionality. Unlike carboplatin, cisplatin showed significant cardiovascular-related reporting signals, including pulmonary embolism (ROR = 3.08, 95% CI: 2.40–3.95; IC = 1.58), aortic thrombosis (ROR = 75.37, 95% CI: 46.04–123.39; IC = 5.37), and myocardial infarction (ROR = 7.05, 95% CI: 4.65–10.70; IC = 2.72). Furthermore, several rare yet highly specific adverse events, notably renal salt-wasting syndrome (ROR = 242.00, 95% CI: 94.66–618.69; IC = 6.06), appeared to be uniquely reported with cisplatin exposure. Detailed results are presented in Tables 3, 4.

Although both agents showed hematologic toxicity-related reporting signals, carboplatin demonstrated a stronger association with bone marrow failure patterns. Conversely, cisplatin displayed a multi-organ reporting profile predominantly involving renal and cardiovascular events. Comprehensive results for all signals meeting the predefined significance criteria are provided in Supplementary Table S5 (All qualifying Preferred Terms associated with carboplatin, *n* = 30,361) and Supplementary Table S6 (All qualifying Preferred Terms associated with cisplatin, *n* = 5,303).

### 3.4 Subgroup analysis

To further explore the demographic heterogeneity in the reporting patterns of adverse events (AEs) related to carboplatin and cisplatin, this study stratified the data by age (<65 years vs. ≥65 years) and sex (male vs. female), and conducted subgroup comparative analyses of the reporting ratios of the two drugs at the PT level. Additionally, to assess whether the main PT-level signals might be driven by the imbalanced distribution of age or sex, this study performed age- and sex-restricted sensitivity analyses within specific demographic strata.

#### 3.4.1 Age subgroup analysis of carboplatin

The age-stratified disproportionality analysis results for carboplatin are shown in Table 5. Among the reported PTs in the two age groups (<65 years and ≥65 years), neutropenic sepsis (ROR = 0.60, 95% CI: 0.40–0.90) and polyneuropathy (ROR = 0.65, 95% CI: 0.42–0.99) had significantly lower reporting ratios in patients <65 years compared to those ≥65 years, suggesting that elderly patients have more prominent reports of carboplatin-related hematological toxicity and neurotoxicity. It is notable that the reporting ratio of reversible posterior encephalopathy syndrome (PRES) was significantly higher in patients <65 years (ROR = 2.32, 95% CI: 1.27–4.24), indicating that this neurological adverse event may be more likely to be reported or occur in younger patients.

**Table 5:** Age-stratified disproportionality analysis of adverse events associated with carboplatin at the preferred term level.

PT	Age < 65 (*n*)	Age ≥ 65 (*n*)	ROR (95% CI)
Both age groups			
Pancytopenia	195	298	0.92 (0.77–1.10)
Leukopenia	129	172	1.05 (0.84–1.33)
Septic shock	58	96	0.85 (0.61–1.18)
Mucosal inflammation	62	81	1.08 (0.77–1.50)
Neutropenic sepsis	34	79	0.60 (0.40–0.90)
Polyneuropathy	31	67	0.65 (0.42–0.99)
Febrile bone marrow aplasia	22	44	0.70 (0.42–1.17)
Posterior reversible encephalopathy syndrome	28	17	2.32 (1.27–4.24)
Immune-mediated hypothyroidism	18	24	1.05 (0.57–1.94)
Bicytopenia	13	19	0.96 (0.47–1.95)
Congenital aplasia	14	16	1.23 (0.60–2.52)
Immune-mediated hyperthyroidism	6	17	0.50 (0.20–1.26)
Endocarditis	12	8	2.11 (0.86–5.16)

For age-stratified analyses, RORs compare patients aged <65 years with those aged ≥65 years; ROR >1 indicates higher reporting odds in patients aged <65 years.

#### 3.4.2 Sex subgroup analysis of carboplatin

The results of the sex-stratified analysis of carboplatin are presented in Table 6. Among the various bone marrow suppression-related adverse events reported in both male and female patients, the reporting ratios of multiple events were higher in male patients: pancytopenia (ROR = 1.31, 95% CI: 1.09–1.57), neutropenia (ROR = 1.34, 95% CI: 1.11–1.62), thrombocytopenia (ROR = 1.42, 95% CI: 1.17–1.73), and leukopenia (ROR = 1.49, 95% CI: 1.17–1.89). Additionally, the reporting ratios of septic shock (ROR = 2.50, 95% CI: 1.70–3.67) and agranulocytosis (ROR = 1.85, 95% CI: 1.14–3.02) were significantly higher in males. Conversely, the reporting ratios of PRES in female patients were significantly higher than those in males (ROR = 0.44, 95% CI: 0.24–0.82), and anaphylactic shock (ROR = 0.62, 95% CI: 0.40–0.98) showed a relatively enriched trend in females.

**Table 6:** Sex-stratified disproportionality analysis of adverse events associated with carboplatin at the preferred term level.

PT	Male (*n*)	Female (*n*)	ROR (95% CI)
Both sexes			
Pancytopenia	334	174	1.31 (1.09–1.57)
Neutropenia	320	163	1.34 (1.11–1.62)
Thrombocytopenia	313	150	1.42 (1.17–1.73)
Leukopenia	214	98	1.49 (1.17–1.89)
Septic shock	121	33	2.50 (1.70–3.67)
Mucosal inflammation	94	57	1.12 (0.80–1.56)
Neutropenic sepsis	72	49	1.00 (0.69–1.43)
Therapy partial responder	73	46	1.08 (0.74–1.56)
Polyneuropathy	70	38	1.25 (0.84–1.86)
Hepatic cytolysis	52	33	1.07 (0.69–1.65)
Agranulocytosis	60	22	1.85 (1.14–3.02)
Rash maculo-papular	50	32	1.06 (0.68–1.65)
Hypomagnesaemia	39	39	0.68 (0.43–1.06)
Anaphylactic shock	36	39	0.62 (0.40–0.98)
Therapeutic product effect incomplete	33	34	0.66 (0.41–1.06)
Febrile bone marrow aplasia	41	25	1.11 (0.68–1.83)
Hypertransaminasaemia	35	23	1.03 (0.61–1.75)
Immune-mediated hypothyroidism	26	17	1.04 (0.56–1.91)
Posterior reversible encephalopathy syndrome	17	26	0.44 (0.24–0.82)
Acute myeloid leukaemia	27	13	1.41 (0.73–2.73)
Bicytopenia	24	7	2.33 (1.00–5.40)
Congenital aplasia	20	10	1.36 (0.63–2.90)
Female specific			
Anaemia	–	258	–
Febrile neutropenia	–	145	–
Acute kidney injury	–	129	–
Hypokalaemia	–	74	–
Hypothyroidism	–	68	–
Colitis	–	65	–
Condition aggravated	–	60	–
Hepatitis	–	48	–
Hyperthyroidism	–	41	–
Immune-mediated enterocolitis	–	39	–
Male specific			
Hepatocellular injury	37	–	–
Toxic epidermal necrolysis	35	–	–
Encephalitis autoimmune	18	–	–
Aphthous ulcer	16	–	–
Acute generalised exanthematous pustulosis	16	–	–
Faecaloma	11	–	–
Peripheral sensorimotor neuropathy	11	–	–
Demyelinating polyneuropathy	11	–	–
Lip oedema	10	–	–
Progressive multifocal leukoencephalopathy	10	–	–

For sex-stratified analyses, RORs compare male patients with female patients; ROR >1 indicates higher reporting odds in male patients.

In terms of sex-specific signals, the main signals unique to female patients include anaemia (*n* = 258), febrile neutropenia (*n* = 145), acute kidney injury (*n* = 129), hypokalaemia (*n* = 74), hypothyroidism (*n* = 68), colitis (*n* = 65), condition aggravated (*n* = 60), hepatitis (*n* = 48), and hyperthyroidism (*n* = 41), suggesting that these events were more frequently reported in female patients, including endocrine dysfunction, renal impairment, and inflammatory gastrointestinal reactions. The signals specific to male patients include toxic epidermal necrolysis (*n* = 35), autoimmune encephalitis (*n* = 18), and demyelinating polyneuropathy (*n* = 11), indicating that male patients had more severe skin adverse reactions and demyelinating neurological lesions associated with carboplatin.

#### 3.4.3 Age subgroup analysis of cisplatin

The results of the age-stratified analysis of cisplatin are shown in Table 7. Among the PTs reported in both age groups, the reporting frequency of neutropenic sepsis in patients aged <65 years was significantly lower than that in patients aged ≥65 years (ROR = 0.35, 95% CI: 0.17–0.74), which was consistent with the age pattern of carboplatin. Renal salt-wasting syndrome was relatively enriched in patients aged <65 years (ROR = 2.81, 95% CI: 0.80–9.87). Although the number of events was small and the association did not reach statistical significance, this finding still suggested that this electrolyte disorder adverse event might be more prominent in young cisplatin users.

**Table 7:** Age-stratified disproportionality analysis of adverse events associated with cisplatin at the preferred term level.

PT	Age < 65 (*n*)	Age ≥ 65 (*n*)	ROR (95% CI)
Both age groups			
Neutropenia	69	55	0.81 (0.56–1.15)
Acute kidney injury	48	37	0.84 (0.54–1.29)
Pancytopenia	37	32	0.74 (0.46–1.20)
Septic shock	17	18	0.61 (0.31–1.18)
Renal salt-wasting syndrome	13	3	2.81 (0.80–9.87)
Nephropathy toxic	12	8	0.97 (0.40–2.38)
Acute myocardial infarction	12	10	0.77 (0.33–1.80)
Neutropenic sepsis	11	20	0.35 (0.17–0.74)
Gastrointestinal toxicity	11	5	1.42 (0.49–4.10)
Renal tubular necrosis	9	10	0.58 (0.24–1.43)
Peripheral artery thrombosis	5	4	0.81 (0.22–3.01)
Arterial thrombosis	4	6	0.43 (0.12–1.53)
Cerebral venous thrombosis	4	3	0.86 (0.19–3.85)

For age-stratified analyses, RORs compare patients aged <65 years with those aged ≥65 years; ROR >1 indicates higher reporting odds in patients aged <65 years.

#### 3.4.4 Sex subgroup analysis of cisplatin

The results of the stratified analysis of cisplatin are presented in Table 8. In the PTs reported by both men and women, the reporting odds of acute kidney injury were numerically higher in men (ROR = 1.39, 95% CI: 0.90–2.14), and febrile neutropenia (ROR = 1.32, 95% CI: 0.85–2.05), pancytopenia (ROR = 1.68, 95% CI: 0.97–2.90), and leukopenia (ROR = 1.61, 95% CI: 0.90–2.88) were also relatively enriched in men. Renal tubular necrosis showed a statistically significant sex-related reporting difference (ROR = 0.36, 95% CI: 0.14–0.93). Because the ROR was parameterized as male versus female, this estimate indicates relative enrichment in female reports rather than male reports.

**Table 8:** Sex-stratified disproportionality analysis of adverse events associated with cisplatin at the preferred term level.

PT	Male (*n*)	Female (*n*)	ROR (95% CI)
Both sexes			
Neutropenia	76	57	0.83 (0.59–1.18)
Acute kidney injury	66	30	1.39 (0.90–2.14)
Febrile neutropenia	63	30	1.32 (0.85–2.05)
Pancytopenia	48	18	1.68 (0.97–2.90)
Leukopenia	41	16	1.61 (0.90–2.88)
Neutropenic sepsis	22	7	1.97 (0.84–4.63)
Aortic thrombosis	16	13	0.77 (0.37–1.60)
Acute myocardial infarction	13	9	0.90 (0.39–2.12)
Renal salt-wasting syndrome	9	7	0.80 (0.30–2.16)
Tinnitus	8	6	0.83 (0.29–2.41)
Renal infarct	7	4	1.10 (0.32–3.75)
Renal tubular necrosis	7	12	0.36 (0.14–0.93)
Cytomegalovirus oesophagitis	6	3	1.25 (0.31–5.01)
Arterial occlusive disease	5	4	0.78 (0.21–2.92)
Cerebral venous thrombosis	4	3	0.83 (0.19–3.73)
Melanoderma	3	5	0.38 (0.09–1.57)
Ototoxicity	3	3	0.63 (0.13–3.10)
Peripheral artery thrombosis	3	7	0.27 (0.07–1.04)
Male specific			
Pulmonary embolism	39	–	–
Renal failure	35	–	–
Septic shock	33	–	–
Nephropathy toxic	20	–	–
Multiple organ dysfunction syndrome	18	–	–
Hypomagnesaemia	12	–	–
Orthostatic hypotension	11	–	–
Inappropriate antidiuretic hormone secretion	10	–	–
Enteritis	9	–	–
Hypocalcaemia	9	–	–
Female specific			
Sepsis	–	19	–
Gastrointestinal toxicity	–	13	–
Lung adenocarcinoma	–	11	–
Oesophagitis	–	9	–
Swelling face	–	9	–
Bone marrow failure	–	9	–
Capillary leak syndrome	–	9	–
Posterior reversible encephalopathy syndrome	–	9	–
Embolism	–	8	–
Ischaemic stroke	–	7	–

For sex-stratified analyses, RORs compare male patients with female patients; ROR >1 indicates higher reporting odds in male patients.

In terms of sex-specific signals, the main signals identified only in male reports included pulmonary embolism (*n* = 39), renal failure (*n* = 35), septic shock (*n* = 33), toxic nephropathy (*n* = 20), multiple organ dysfunction syndrome (*n* = 18), hypomagnesaemia (*n* = 12), and inappropriate antidiuretic hormone secretion syndrome (*n* = 10). Overall, these findings indicate that severe renal toxicity, infectious complications, and thromboembolic events were more frequently reported among male cisplatin-associated reports, whereas gastrointestinal, hematologic, and neurological events were relatively more prominent in female cisplatin-associated reports. The signals identified only in female reports included sepsis (*n* = 19), gastrointestinal toxicity (*n* = 13), oesophagitis (*n* = 9), bone marrow failure (*n* = 9), capillary leak syndrome (*n* = 9), and PRES (*n* = 9), suggesting relatively more frequent reporting of gastrointestinal, hematologic, and neurological events among female cisplatin-associated reports.

#### 3.4.5 Age- and sex-stratified sensitivity analyses

To further evaluate whether the positive signals of the above PT levels were driven by the imbalance in age or sex distribution between the two drugs, this study conducted repeated tests by restricting the disproportionality analysis to four independent demographic strata: patients aged <65 years, patients aged ≥65 years, male patients, and female patients (Supplementary Tables S11A–D, S12A–D).

The age-restricted analysis of carboplatin showed that the core signals persisted in both age subgroups: in patients aged <65 years, pancytopenia (ROR = 5.69, 95% CI: 4.80–6.73), leukopenia (ROR = 4.70, 95% CI: 3.84–5.76), septic shock (ROR = 3.43, 95% CI: 2.57–4.58), polyneuropathy (ROR = 7.03, 95% CI: 4.54–10.90), and PRES (ROR = 3.93, 95% CI: 2.58–6.01) were all positive signals; in the subgroup of patients aged ≥65 years, thrombocytopenia (ROR = 3.65, 95% CI: 3.21–4.15), pancytopenia (ROR = 5.83, 95% CI: 5.08–6.70), leukopenia (ROR = 5.05, 95% CI: 4.23–6.03), polyneuropathy (ROR = 8.36, 95% CI: 6.13–11.41), and PRES (ROR = 5.66, 95% CI: 3.19–10.05) also remained significantly positive.

The sex-restricted analysis of carboplatin showed that in the male subgroup, pancytopenia (ROR = 5.77, 95% CI: 5.07–6.58), neutropenia (ROR = 2.95, 95% CI: 2.61–3.33), thrombocytopenia (ROR = 3.08, 95% CI: 2.72–3.49), and leukopenia (ROR = 5.15, 95% CI: 4.39–6.04) were all positive signals; in the female subgroup, these signals also persisted, including pancytopenia (ROR = 7.18, 95% CI: 6.02–8.56), neutropenia (ROR = 3.61, 95% CI: 3.05–4.27), thrombocytopenia (ROR = 3.64, 95% CI: 3.05–4.34), and leukopenia (ROR = 5.56, 95% CI: 4.43–6.97), as well as anaemia (ROR = 3.20, 95% CI: 2.80–3.65), which was also a significant positive signal.

The age-restricted analysis of cisplatin showed that in patients aged <65 years, neutropenia (ROR = 3.79, 95% CI: 2.96–4.86), acute kidney injury (ROR = 3.41, 95% CI: 2.54–4.57), and aortic thrombosis (ROR = 72.41, 95% CI: 41.07–127.67) remained positive disproportionality signals in patients aged <65 years. In patients aged ≥65 years, neutropenia (ROR = 4.74, 95% CI: 3.61–6.23), acute kidney injury (ROR = 3.21, 95% CI: 2.31–4.46), tubular necrosis (ROR = 17.75, 95% CI: 9.10–34.62), and febrile neutropenia (ROR = 4.36, 95% CI: 3.30–5.75) were also persistent core signals. The sex-restricted analysis of cisplatin revealed that in the male subgroup, neutropenia (ROR = 3.50, 95% CI: 2.78–4.42), acute kidney injury (ROR = 3.64, 95% CI: 2.84–4.67), and aortic thrombosis (ROR = 118.89, 95% CI: 53.91–262.20) were all significantly positive; in the female subgroup, core signals such as neutropenia (ROR = 7.08, 95% CI: 5.40–9.28), acute kidney injury (ROR = 4.10, 95% CI: 2.84–5.92), and renal tubular necrosis (ROR = 17.90, 95% CI: 9.73–32.95) remained significantly positive as well.

### 3.5 Time-to-onset analysis

Among all eligible reports, valid time-to-onset (TTO) information was available for 4,487 of 10,159 carboplatin-associated reports (44.2%) and 823 of 1,886 cisplatin-associated reports (43.6%), as both the therapy start date and the adverse event onset date were available and chronologically valid. Reports with missing, incomplete, or chronologically invalid date information were excluded from the TTO analysis.

Table 9 summarizes the overall TTO characteristics and Weibull distribution analysis among reports with valid TTO information. The median TTO was 25 days for carboplatin (IQR, 8–67 days), with an observed TTO range of 1 to 1,625 days. For cisplatin, the median TTO was 21 days (IQR, 8–62 days), with an observed TTO range of 1 to 2,757 days. In the Weibull distribution analysis, both agents showed shape parameters below 1. The *β* value was 0.73 for carboplatin (95% CI, 0.72–0.75) and 0.73 for cisplatin (95% CI, 0.69–0.76), with the upper bounds of both 95% CIs remaining below 1. The corresponding scale parameters were 48.42 for carboplatin (95% CI, 46.41–50.52) and 41.93 for cisplatin (95% CI, 37.94–46.33). These findings indicate an early-failure pattern for both carboplatin- and cisplatin-associated adverse event reports Table 10.

**Table 9:** Overall time-to-onset characteristics and Weibull distribution analysis of adverse events associated with carboplatin and cisplatin in the FAERS database.

Drug	Overall TTO description			Weibull distribution analysis				Failure type
	*n*	Median (IQR)	Min–Max	*α*	95% CI	*β*	95% CI	
Carboplatin	4,487	25 (8–67)	1–1,625	48.42	46.41–50.52	0.73	0.72–0.75	Early failure
Cisplatin	823	21 (8–62)	1–2,757	41.93	37.94–46.33	0.73	0.69–0.76	Early failure

TTO, time-to-onset; IQR, interquartile range; CI, confidence interval. TTO was calculated only for reports with available and chronologically valid therapy start and adverse event onset dates. The Weibull scale parameter (*α*) and shape parameter (*β*) are reported with 95% CIs. A *β*-value <1 indicates an early-failure pattern.

**Table 10:** Time-to-onset of adverse events associated with carboplatin and cisplatin by system organ class in the FAERS database.

System organ class	Carboplatin		Cisplatin	
	*n*	Median (IQR)	*n*	Median (IQR)
Blood and lymphatic system disorders	1,453	14 (7–40)	262	41 (10–66)
Renal and urinary disorders	462	22 (8–69)	111	13 (8–41)
Hepatobiliary disorders	373	26 (11–62)	–	–
Ear and labyrinth disorders	–	–	19	41 (10–66)

TTO, time-to-onset; SOC, System Organ Class; IQR, interquartile range. Values are presented as the number of reports with valid TTO information and median TTO in days (IQR). TTO was defined as the interval between the reported therapy start date and the adverse event onset date. Only reports with available and chronologically valid start and onset dates were included. A dash indicates that the corresponding SOC was not included in the selected SOC-specific TTO summary for that platinum agent.

**Figure 4:**
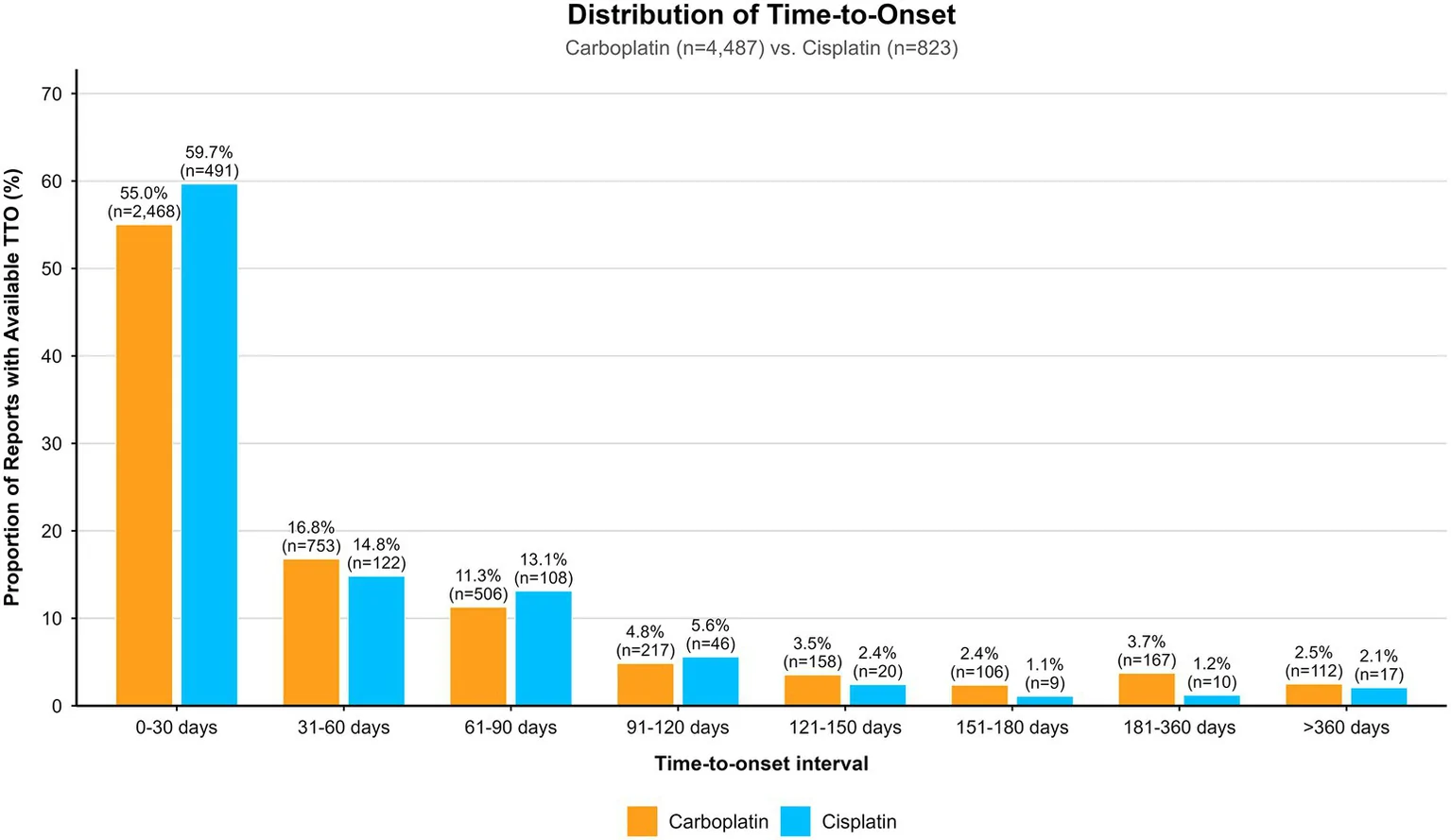
Time-to-onset distribution of adverse events associated with carboplatin and cisplatin in the FAERS database.

The interval-based TTO distribution is shown in Figure 4. Among reports with valid TTO information, the largest proportion of reports fell within the 0–30-day interval for both agents. Specifically, 55.0% of carboplatin-associated reports were recorded within 0–30 days (*n* = 2,468/4,487), compared with 59.7% of cisplatin-associated reports (*n* = 491/823). The proportion of reports declined across subsequent time intervals, although reports occurring beyond 180 days were still observed for both agents.

Table 10 presents TTO characteristics for selected SOCs. For blood and lymphatic system disorders, the median TTO was 14 days for carboplatin (IQR, 7–40 days) and 41 days for cisplatin (IQR, 10–66 days). For renal and urinary disorders, the median TTO was 22 days for carboplatin (IQR, 8–69 days) and 13 days for cisplatin (IQR, 8–41 days). In the selected SOC summary, hepatobiliary disorders were included only for carboplatin, with a median TTO of 26 days (IQR, 11–62 days), whereas ear and labyrinth disorders were included only for cisplatin, with a median TTO of 41 days (IQR, 10–66 days).

### 3.6 Sensitivity analysis

Sensitivity analyses were performed to evaluate the robustness of the primary PT-level disproportionality findings after separately excluding reports with selected co-reported chemotherapy agents. Three separate exclusion analyses were conducted for each platinum agent: exclusion of pemetrexed co-reports, exclusion of paclitaxel co-reports, and exclusion of albumin-bound paclitaxel co-reports. For carboplatin, the results of these three analyses are presented in Supplementary Tables S9A–C, respectively. For cisplatin, the corresponding analyses are presented in Supplementary Tables S10A–C.

Across these separate exclusion analyses, the principal signal profiles remained broadly consistent with the primary analysis. For carboplatin, hematologic signals remained prominent in Supplementary Tables S9A–C, including anaemia, neutropenia, pancytopenia, thrombocytopenia, febrile neutropenia, leukopenia, neutropenic sepsis, and agranulocytosis. Additional clinically interpretable signals involving renal injury, electrolyte disturbances, hypersensitivity reactions, hepatobiliary injury, mucosal inflammation, and neurologic events were also retained.

For cisplatin, the main PT-level reporting pattern was similarly preserved in Supplementary Tables S10A–C. Persistent signals included hematologic events, renal and urinary events, vascular thrombotic events, gastrointestinal toxicity, and auditory/vestibular events. Representative retained PTs included neutropenia, febrile neutropenia, acute kidney injury, pancytopenia, pulmonary embolism, leukopenia, renal failure, septic shock, neutropenic sepsis, renal tubular necrosis, renal salt-wasting syndrome, tinnitus, and gastrointestinal toxicity.

Overall, the persistence of the main PT-level signals after separately excluding selected co-reported chemotherapy agents suggests that the principal disproportionality patterns were not solely driven by pemetrexed, paclitaxel, or albumin-bound paclitaxel co-reporting. However, these analyses should be interpreted as robustness checks rather than complete adjustment for confounding from combination regimens, cancer stage, treatment line, cumulative dose, or underlying clinical factors.

## 4 Discussion

This pharmacovigilance analysis, based on over 12,000 FAERS reports involving patients with lung cancer, describes and compares the reported adverse event patterns associated with carboplatin and cisplatin in a real-world spontaneous reporting setting. The findings indicate potential differences in the adverse-event reporting profiles of these two platinum agents and may help inform clinical vigilance and safety monitoring considerations. However, these results should be interpreted cautiously, as FAERS data cannot establish incidence rates, causality, or direct comparative risk (17).

The demographic and temporal distributions observed in this study further suggest that the reported safety profiles of carboplatin and cisplatin may be influenced by differences in patient selection, clinical use patterns, and reporting behaviors. As shown in Table 10, carboplatin-related reports involved a larger proportion of older patients: patients aged ≥65 years accounted for 4,868 cases (47.9%) in the carboplatin group, compared with 622 cases (33.0%) in the cisplatin group. Conversely, patients aged 18–64 years were more frequently represented in the cisplatin group than in the carboplatin group (954 cases, 50.6% vs. 3,555 cases, 35.0%). The sex distribution was broadly similar between the two groups, with males accounting for 5,440 cases (53.5%) in the carboplatin group and 1,041 cases (55.2%) in the cisplatin group. Notably, body weight information was missing in more than 60% of reports for both agents (6,295 cases, 62.0% for carboplatin, 1,187 cases, 62.9% for cisplatin), which limits further adjustment for body size-related factors. This demographic pattern is clinically plausible, as carboplatin is often preferred in older or less fit patients because of its generally more favorable tolerability profile and lower requirements for hydration and renal function monitoring compared with cisplatin (31). Therefore, the observed differences in adverse event reporting should be interpreted considering potential confounding by age, comorbidity burden, body size, and treatment selection rather than as a direct comparison of intrinsic drug toxicity.

The temporal trends also showed a marked divergence between the two platinum agents (Figure 2). Carboplatin-related AE reports increased substantially after 2017, rising from 419 reports in 2017 to 856 reports in 2018, and continued to increase thereafter, reaching 1,356 reports in 2024, which accounted for the highest annual proportion of carboplatin-related reports (13.3%). A similarly high number of carboplatin-related reports was observed in 2025, with 1,299 reports (12.8%). In contrast, cisplatin-related reports peaked in 2019, with 204 reports accounting for 10.8% of all cisplatin-related reports, and subsequently declined to 61 reports (3.2%) by 2025. The lower number of reports in 2026 should be interpreted cautiously because only Q1 data were included.

This opposite temporal pattern may partly reflect the evolving treatment landscape of lung cancer, including the increasing adoption of carboplatin-containing combination regimens in advanced lung cancer (32), as well as a relative shift away from cisplatin in patients who are elderly, frail, or have renal vulnerability. In addition, changes in post-marketing surveillance intensity, reporting practices, drug utilization, and country-specific reporting patterns may have contributed to the observed annual fluctuations. Importantly, because FAERS is a spontaneous reporting database, these temporal changes cannot be used to estimate the true incidence of adverse events or to infer a causal change in comparative safety risk over time. Therefore, the apparent decline in cisplatin reports after 2019 and the increase in carboplatin reports after 2017 should be regarded as changes in reporting patterns rather than definitive evidence of decreasing cisplatin toxicity or increasing carboplatin toxicity.

At both the SOC and PT levels, carboplatin and cisplatin demonstrated overlapping but distinguishable patterns of reported adverse events. As shown in Table 1 and Figure 3, after excluding SOC categories less directly related to adverse drug reactions, both platinum agents showed signals across a broad range of organ systems, suggesting that platinum-based chemotherapy in lung cancer is associated with complex multi-system safety concerns in real-world spontaneous reports. However, the distribution of algorithm-positive SOCs and high-frequency PTs differed between the two agents, indicating that carboplatin and cisplatin may have different reporting profiles rather than identical toxicity patterns.

For carboplatin, the most prominent PT-level signals were concentrated in blood and lymphatic system disorders. Significant disproportionality signals were observed for anaemia (ROR = 2.43, 95% CI: 2.23–2.65; IC = 1.14), neutropenia (ROR = 3.10, 95% CI: 2.83–3.40; IC = 1.44), and pancytopenia (ROR = 6.42, 95% CI: 5.80–7.11; IC = 2.25), supporting the well-recognized myelosuppressive profile of carboplatin (33). Notably, neutropenic sepsis also showed a strong reporting signal (ROR = 7.22, 95% CI: 5.93–8.80; IC = 2.37), suggesting that infection-related complications secondary to bone marrow suppression should be closely monitored, particularly in vulnerable patients or those receiving combination regimens.

In addition to hematologic toxicity, carboplatin-related reports also showed signals involving hepatic injury and hypersensitivity reactions. Hepatic cytolysis (ROR = 3.40, 95% CI: 2.69–4.31; IC = 1.56), hypertransaminasaemia (ROR = 5.22, 95% CI: 3.92–6.96; IC = 2.04), and hepatocellular injury (ROR = 3.43, 95% CI: 2.57–4.58; IC = 1.57) all met the predefined signal criteria (34), suggesting that liver-related adverse event reports deserve attention in patients receiving carboplatin-containing regimens. Anaphylactic shock was also identified as a notable signal (ROR = 4.71, 95% CI: 3.67–6.05; IC = 1.63), which is consistent with the clinical concern regarding platinum-associated hypersensitivity reactions (35). Nevertheless, these findings should not be interpreted as definitive evidence of causality, as concomitant anticancer therapies, immune checkpoint inhibitors, underlying disease progression, and incomplete clinical information may have contributed to these signals.

In contrast, cisplatin exhibited a broader multi-organ signal profile, with prominent involvement of renal, vascular, cardiovascular, neurological, gastrointestinal, and ear/labyrinth system disorders. At the PT level, acute kidney injury (ROR = 3.80, 95% CI: 3.10–4.66; IC = 1.86) and renal failure (ROR = 2.99, 95% CI: 2.23–4.02; IC = 1.54) were important signals, supporting the known renal vulnerability associated with cisplatin exposure (36). The lower reporting signal for renal and urinary disorders in the carboplatin group compared with cisplatin (ROR = 0.66, 95% CI: 0.58–0.76) is consistent with the clinical perception that carboplatin is generally less nephrotoxic than cisplatin.

Cisplatin also showed notable signals related to vascular and cardiovascular events, including pulmonary embolism (ROR = 3.08, 95% CI: 2.40–3.95; IC = 1.58), aortic thrombosis (ROR = 75.37, 95% CI: 46.04–123.39; IC = 5.37), and myocardial infarction (ROR = 7.05, 95% CI: 4.65–10.70; IC = 2.72). In addition, renal salt-wasting syndrome showed an especially strong disproportionality signal (ROR = 242.00, 95% CI: 94.66–618.69; IC = 6.06) (37), although this finding should be interpreted cautiously because very high ROR values may be influenced by small case numbers and reporting selectivity. These signals may reflect both potential drug-related renal and vascular toxicity and the high baseline thrombotic risk of patients with lung cancer. Therefore, they support the need for careful renal, electrolyte, cardiovascular, and thrombotic risk assessment in patients receiving cisplatin-based therapy.

At the SOC level, blood and lymphatic system disorders and infection-related disorders were commonly reported for both agents, and direct comparison did not show a clear difference between carboplatin and cisplatin for these two SOCs (blood and lymphatic system disorders: ROR = 0.91, 95% CI: 0.84–1.00; infections and infestations: ROR = 1.01, 95% CI: 0.90–1.13). However, cisplatin-specific SOC signals were more widely distributed, involving gastrointestinal disorders, nervous system disorders, vascular disorders, metabolism and nutrition disorders, cardiac disorders, and ear and labyrinth disorders. The identification of ear and labyrinth disorders is consistent with the established ototoxic potential of cisplatin (34) and highlights the importance of monitoring clinically important but less frequent toxicities that may not always appear among the most frequently reported PTs.

Overall, the present findings suggest that carboplatin-related reports were mainly characterized by myelosuppression, infection-related complications, hypersensitivity reactions, and hepatic injury signals, whereas cisplatin-related reports showed a more extensive multi-organ reporting pattern, especially involving renal, vascular, cardiovascular, neurological, and ototoxicity-related events. These differences may provide useful clues for risk awareness and individualized monitoring in lung cancer treatment. Nevertheless, these results should be interpreted within the limitations of FAERS. Disproportionality analysis identifies reporting signals rather than true incidence rates or causal associations. Differences in report counts may be influenced by drug utilization, treatment selection, concomitant therapies, cancer stage, reporting behavior, regional reporting practices, and missing clinical information. Therefore, the observed SOC- and PT-level differences should be regarded as comparative reporting patterns rather than direct evidence of comparative toxicity risk between carboplatin and cisplatin.

The age- and sex-stratified analyses suggested that the reporting patterns of carboplatin- and cisplatin-related adverse events were not completely homogeneous across demographic subgroups. For carboplatin, age-stratified comparisons were focused on PTs that were concurrently identified as positive signals by all four disproportionality algorithms, with the main comparative results summarized in Table 4 and the complete age-related signal list provided in Supplementary Table S7A. Neutropenic sepsis was reported less frequently in patients aged <65 years than in those aged ≥65 years (ROR = 0.60, 95% CI: 0.40–0.90), and a similar pattern was observed for polyneuropathy (ROR = 0.65, 95% CI: 0.42–0.99). These findings suggest relatively greater reporting of infection-related hematologic complications and neurotoxicity among older patients receiving carboplatin. This is clinically plausible, as older patients may have poorer bone marrow reserve, more comorbidities, and greater vulnerability to treatment-related complications (38). In contrast, posterior reversible encephalopathy syndrome (PRES) (39) showed a higher reporting ratio in patients aged <65 years (ROR = 2.32, 95% CI: 1.27–4.24), suggesting a potentially different age-related reporting pattern for this rare neurologic event. However, given the rarity of PRES and the limited clinical information available in FAERS, this finding should be considered hypothesis-generating rather than confirmatory.

For carboplatin, sex-stratified comparisons were similarly restricted to PTs detected as positive signals by all four algorithms, with the main results and sex-exclusive PTs presented in Table 5 and the full signal list shown in Supplementary Table S7B. Several myelosuppression-related PTs were more frequently reported in male patients, including pancytopenia (ROR = 1.31, 95% CI: 1.09–1.57), neutropenia (ROR = 1.34, 95% CI: 1.11–1.62), thrombocytopenia (ROR = 1.42, 95% CI: 1.17–1.73), and leukopenia (ROR = 1.49, 95% CI: 1.17–1.89). Septic shock (ROR = 2.50, 95% CI: 1.70–3.67) and agranulocytosis (ROR = 1.85, 95% CI: 1.14–3.02) were also more prominently reported in males. Conversely, PRES showed a female-enriched reporting pattern, as reflected by a lower ROR in males versus females (ROR = 0.44, 95% CI: 0.24–0.82), and anaphylactic shock also appeared relatively enriched in females (ROR = 0.62, 95% CI: 0.40–0.98). From the perspective of sex-specific pharmacology, these findings may partly reflect sex-related differences in body composition, renal clearance, drug distribution, immune response (40), inflammatory reactivity, and susceptibility to hypersensitivity reactions. Although carboplatin dosing is generally adjusted according to renal function and target area under the curve, residual differences in renal reserve, marrow reserve, treatment tolerance, and concomitant therapies may still contribute to sex-related reporting heterogeneity. Nevertheless, these mechanistic explanations remain speculative because FAERS lacks detailed information on dose intensity, renal function, treatment regimen, comorbidities, and supportive care.

For cisplatin, age-stratified comparisons of PTs meeting the four-algorithm positivity criteria are summarized in Table 6, with complete results provided in Supplementary Table S8A. Neutropenic sepsis was reported less frequently in patients aged <65 years than in those aged ≥65 years (ROR = 0.35, 95% CI: 0.17–0.74), which was consistent with the age-related pattern observed for carboplatin. Renal salt-wasting syndrome showed a numerically higher reporting ratio in younger cisplatin users (ROR = 2.81, 95% CI: 0.80–9.87), although this association did not reach statistical significance and was based on limited reports. These findings suggest that age may influence the reporting pattern of selected cisplatin-related hematologic and renal-electrolyte adverse events (38), but the limited number of reports for rare PTs requires cautious interpretation.

For cisplatin, sex-stratified comparisons and sex-exclusive PTs are presented in Table 7, with the complete sex-related signal list provided in Supplementary Table S8B. Acute kidney injury (ROR = 1.39, 95% CI: 0.90–2.14), febrile neutropenia (ROR = 1.32, 95% CI: 0.85–2.05), pancytopenia (ROR = 1.68, 95% CI: 0.97–2.90), and leukopenia (ROR = 1.61, 95% CI: 0.90–2.88) showed numerically higher reporting ratios in males, although these findings were not statistically significant. Renal tubular necrosis showed a statistically significant sex-related reporting difference (ROR = 0.36, 95% CI: 0.14–0.93). Because the sex-stratified ROR was parameterized as male versus female, the ROR below 1.0 for renal tubular necrosis indicates relative enrichment in female reports rather than male reports. This female-enriched reporting pattern could be pharmacologically plausible, because cisplatin-associated nephrotoxicity is closely related to renal tubular accumulation, tubular epithelial injury, electrolyte disturbance, vascular injury, oxidative stress, and inflammatory responses. Potential contributors may include sex-related differences in renal transporter expression, glomerular filtration, tubular handling, body composition (41), hydration tolerance, baseline cardiovascular risk, and exposure to concomitant nephrotoxic agents. Conversely, signals detected in female reports, such as posterior reversible encephalopathy syndrome, gastrointestinal toxicity, oesophagitis, bone marrow failure, and capillary leak syndrome, may reflect differences in immune-endothelial responses, inflammatory susceptibility, treatment context, or reporting behavior. However, these mechanistic explanations remain speculative and cannot be verified in FAERS, because the database lacks detailed information on cumulative cisplatin exposure, renal function, hydration protocols, performance status, comorbidities, supportive care, and concomitant treatment regimens.

The sex-exclusive PTs identified in this analysis should also be interpreted cautiously. For carboplatin, female-exclusive signals included anaemia, febrile neutropenia, acute kidney injury, hypokalaemia, thyroid dysfunction, colitis, hepatitis, and other inflammatory or endocrine-related events, whereas male-exclusive signals included toxic epidermal necrolysis, autoimmune encephalitis, and demyelinating polyneuropathy (Table 5; Supplementary Table S7B). For cisplatin, male-exclusive signals included pulmonary embolism, renal failure, septic shock, toxic nephropathy, multiple organ dysfunction syndrome, hypomagnesaemia, and inappropriate antidiuretic hormone secretion (42), whereas female-exclusive signals included sepsis, gastrointestinal toxicity, oesophagitis, bone marrow failure, capillary leak syndrome, and PRES (Table 7; Supplementary Table S8B). These findings may help generate hypotheses regarding sex-related differences in toxicity reporting. However, “sex-exclusive” in this analysis only means that the PT was detected in one sex subgroup within the FAERS dataset and should not be interpreted as evidence that the event occurs exclusively in one sex in clinical practice.

A key issue in interpreting these subgroup findings is confounding by indication, or channeling bias. In real-world lung cancer treatment, cisplatin and carboplatin are not randomly assigned. Cisplatin is generally selected for younger and fitter patients with preserved renal function, whereas carboplatin is often used in older, frailer patients or in those with renal impairment and a higher comorbidity burden. Therefore, the higher reporting of neutropenic sepsis and polyneuropathy among older carboplatin-treated patients may reflect both carboplatin-associated myelosuppressive potential and the vulnerable baseline profile of patients clinically channeled toward carboplatin, rather than an intrinsic age-specific toxicity of carboplatin itself. Similarly, observed sex-related differences may be influenced by differences in body composition, renal clearance, baseline comorbidities, treatment regimen, dose intensity, and supportive care, rather than biological sex alone.

The stratum-restricted sensitivity analyses further evaluated whether the main PT-level signals were driven by imbalances in age or sex distribution, with detailed results presented in Supplementary Tables S11A–D, S12A–D. After restricting the analysis to specific age and sex strata, major carboplatin-related signals, including myelosuppression-related PTs, polyneuropathy, and PRES, remained detectable across multiple subgroups. Similarly, key cisplatin-related signals, including neutropenia, acute kidney injury, renal tubular necrosis, febrile neutropenia, and aortic thrombosis, persisted in age- or sex-restricted analyses. These findings suggest that the principal PT-level signals were not solely explained by demographic imbalance and had a degree of relative robustness. However, the simultaneous evaluation of multiple PTs across several demographic strata may still increase the possibility of chance findings, even though concordance across four disproportionality algorithms improves signal reproducibility. In addition, FAERS lacks detailed information on performance status, baseline organ function, creatinine clearance, marrow reserve, cumulative dose, treatment duration, combination regimens, supportive care, cancer stage, and comorbidities. Therefore, the subgroup results should be regarded as exploratory and hypothesis-generating descriptions of differential reporting patterns, rather than causal estimates of age- or sex-specific susceptibility to carboplatin or cisplatin toxicity.

The time-to-onset analysis further demonstrated that adverse event reports associated with both platinum agents were concentrated in the early treatment period. As shown in Figure 4 and Table 8, valid TTO information was available for 4,487 carboplatin-associated reports and 823 cisplatin-associated reports. The median TTO was broadly comparable between the two agents, with 25 days for carboplatin (IQR, 8–67 days) and 21 days for cisplatin (IQR, 8–62 days). More than half of the reports with available TTO occurred within the first 30 days after treatment initiation, including 55.0% of carboplatin-associated reports (2,468/4,487) and 59.7% of cisplatin-associated reports (491/823). These findings suggest that the early phase after platinum administration represents a critical window for safety monitoring in patients with lung cancer.

The Weibull distribution analysis also supported an early-failure pattern for both agents. As summarized in Table 8, the *β*-values were below 1 for both carboplatin (*β* = 0.73, 95% CI: 0.72–0.75) and cisplatin (*β* = 0.73, 95% CI: 0.69–0.76), with the upper limits of both 95% confidence intervals remaining below 1. In pharmacovigilance studies, a *β*-value below 1 is generally interpreted as indicating that the reporting hazard is higher shortly after treatment initiation and decreases over time (43). This pattern is consistent with the interval-based distribution shown in Figure 4, where the highest proportion of reports occurred within 0–30 days and progressively declined across later time intervals. Nevertheless, delayed reports beyond 180 days and even beyond 360 days were still observed for both agents, suggesting that late-onset or long-latency adverse events should not be entirely overlooked, especially in patients receiving prolonged or repeated platinum-based treatment.

The SOC-specific TTO results provided further clinical insight into the temporal characteristics of major toxicity categories (Table 9). For blood and lymphatic system disorders, carboplatin-associated reports had a shorter median TTO than cisplatin-associated reports (14 days, IQR 7–40 days vs. 41 days, IQR 10–66 days), which is consistent with the prominent early myelosuppressive reporting profile observed for carboplatin. This finding supports the importance of early complete blood count monitoring, particularly within the first treatment cycle. In contrast, renal and urinary disorders appeared earlier in cisplatin-associated reports than in carboplatin-associated reports, with a median TTO of 13 days for cisplatin (IQR, 8–41 days) compared with 22 days for carboplatin (IQR, 8–69 days). This temporal pattern is clinically plausible given the known renal vulnerability associated with cisplatin and supports early monitoring of renal function, hydration status, and electrolyte abnormalities after cisplatin administration.

Selected organ-specific patterns were also observed. Hepatobiliary disorders were summarized for carboplatin, with a median TTO of 26 days (IQR, 11–62 days), suggesting that liver-related laboratory abnormalities or hepatic injury reports may emerge relatively early during carboplatin-containing regimens. Ear and labyrinth disorders were summarized for cisplatin, with a median TTO of 41 days (IQR, 10–66 days), which is consistent with the recognized ototoxic potential of cisplatin and highlights the need for hearing-related assessment in susceptible patients, particularly when cisplatin is used repeatedly or at higher cumulative exposure.

However, these TTO findings should be interpreted cautiously. Valid TTO information was available in fewer than half of all included reports for both carboplatin and cisplatin, and reports with missing, incomplete, or chronologically invalid dates were excluded. Therefore, the TTO analysis may be affected by selection bias and incomplete reporting. In addition, FAERS does not provide complete information on treatment cycles, cumulative dose, dose interruptions, rechallenge, supportive care, comorbidities, or concomitant anticancer therapies, all of which may influence the apparent onset time of adverse events. Accordingly, the observed TTO distributions and Weibull early-failure patterns should be regarded as descriptive reporting patterns rather than precise estimates of the true onset risk. Despite these limitations, the findings suggest that early clinical surveillance after platinum initiation is particularly important, with attention to hematologic toxicity for carboplatin and renal, electrolyte, and ototoxicity-related events for cisplatin.

The exclusion-based sensitivity analyses further supported the robustness of the principal PT-level signal profiles observed in the primary analysis. After separately excluding reports co-reported with pemetrexed, paclitaxel, or albumin-bound paclitaxel (44), the major disproportionality patterns for both platinum agents remained broadly consistent, as shown in Supplementary Tables S9A–C, S10A–C. This finding suggests that the main PT-level signals were not solely driven by co-reporting with these selected chemotherapy agents.

For carboplatin, the hematologic signal profile remained prominent across the three sensitivity analyses. Core PT-level signals, including anaemia, neutropenia, pancytopenia, thrombocytopenia, febrile neutropenia, leukopenia, neutropenic sepsis, and agranulocytosis, were retained after the exclusion of each selected co-reported chemotherapy agent. This consistency strengthens the interpretation that myelosuppression and its infectious complications represent a central pharmacovigilance signal pattern for carboplatin in lung cancer reports. Other retained clinically interpretable signals involving renal injury, electrolyte disturbances, hypersensitivity reactions, hepatobiliary injury, mucosal inflammation, and neurologic events further indicate that the carboplatin-associated signal profile was not materially altered by these exclusion criteria.

For cisplatin, the principal PT-level reporting pattern was also preserved in the sensitivity analyses. Persistent signals included hematologic events, renal and urinary events, vascular thrombotic events, gastrointestinal toxicity, and auditory or vestibular events. Representative retained PTs included neutropenia, febrile neutropenia, acute kidney injury, pancytopenia, pulmonary embolism, leukopenia, renal failure, septic shock, neutropenic sepsis, renal tubular necrosis, renal salt-wasting syndrome, tinnitus, and gastrointestinal toxicity. The persistence of renal salt-wasting syndrome across the sensitivity analyses is particularly notable because this PT is clinically specific and biologically plausible in the context of cisplatin-associated renal tubular dysfunction and electrolyte wasting. Although the number of reports was limited, the consistency of this signal after exclusion of selected co-reported chemotherapy agents supports its relevance as a rare but important pharmacovigilance finding.

These sensitivity analyses provide reassurance that the major carboplatin- and cisplatin-associated PT-level signals were not fully explained by pemetrexed, paclitaxel, or albumin-bound paclitaxel co-reporting. However, these analyses should be interpreted as robustness checks rather than definitive adjustment for confounding. FAERS reports do not reliably capture treatment line, dose intensity, cumulative exposure, renal function, performance status, comorbidity burden, radiotherapy, surgery, immunotherapy, targeted therapy, or supportive medications. Therefore, residual confounding from combination regimens and underlying clinical factors remains possible. The persistence of the main signal patterns across the exclusion-based sensitivity analyses and the age- and sex-restricted analyses strengthens the internal consistency of the findings, but it does not establish causality or quantify absolute clinical risk.

### 4.1 Limitations

This study has several limitations. First, FAERS is a spontaneous reporting system and is subject to underreporting, reporting bias, duplicate or incomplete reports, and missing clinical information. Because the total number of exposed patients is unknown, disproportionality analyses cannot estimate incidence, absolute risk, or causal effects. Therefore, our findings should be interpreted as pharmacovigilance safety signals and reporting patterns rather than definitive evidence of drug-specific toxicity.

Second, confounding by indication and channeling bias (17) should be considered when comparing carboplatin and cisplatin. In real-world thoracic oncology practice, cisplatin is more often selected for younger and fitter patients with preserved renal function, whereas carboplatin is commonly used in older, frailer patients or those with impaired renal reserve or comorbidities. Although we performed age- and sex-restricted sensitivity analyses to assess whether selected PT-level signals persisted within demographic strata, these analyses cannot fully adjust for unmeasured clinical factors such as performance status, renal function, comorbidity burden, baseline marrow reserve, dose intensity, treatment line, or supportive care. Accordingly, differences in reporting patterns between the two agents may partly reflect baseline patient selection rather than intrinsic drug toxicity alone.

Third, concomitant anticancer therapies may have contributed to the observed signals. We performed exclusion-based sensitivity analyses after separately excluding reports co-reported with pemetrexed, paclitaxel, or albumin-bound paclitaxel, and the main signal patterns remained broadly consistent. However, these analyses cannot completely account for the influence of other combination regimens, radiotherapy, surgery, targeted therapy, immunotherapy, or supportive medications, particularly because co-medication information in FAERS may be incomplete or inconsistently reported.

Fourth, no formal multiplicity adjustment, such as Bonferroni or false discovery rate correction, was applied across the large number of SOC- and PT-level analyses or subgroup comparisons. To reduce false-positive findings, PT-level signals were required to meet the predefined criteria of all four disproportionality algorithms and were further screened for clinical relevance. Nevertheless, residual false-positive findings cannot be excluded. Conversely, this conservative signal definition may have reduced sensitivity and may have missed rare but clinically relevant adverse event signals.

Finally, the TTO analysis was limited to reports with available and chronologically valid therapy start dates and adverse event onset dates. Valid TTO could be calculated for 4,487 of 10,159 carboplatin-associated reports and 823 of 1,886 cisplatin-associated reports. Excluding reports with missing or incomplete date information may have introduced selection bias, and severe or early-onset events may have been more likely to have complete date documentation. Therefore, the TTO findings, including the Weibull early-failure patterns, should be interpreted as descriptive temporal reporting patterns among reports with valid date information. Further validation using prospective cohorts, electronic health record-based studies, or clinical trial datasets with more complete clinical covariates is warranted.

## 5 Conclusion

In this lung cancer–specific FAERS pharmacovigilance analysis, carboplatin and cisplatin showed distinct but partially overlapping adverse-event reporting profiles. Carboplatin-related reports were mainly characterized by hematologic and infection-related signals, including myelosuppression-associated complications, together with hepatobiliary and hypersensitivity-related signals. In contrast, cisplatin-related reports showed a broader multi-organ signal pattern, particularly involving renal and urinary events, vascular thrombotic events, auditory or vestibular events, gastrointestinal toxicity, and the rare but clinically specific signal of renal salt-wasting syndrome.

The time-to-onset analysis further indicated that adverse-event reports for both platinum agents were concentrated early after treatment initiation. Among reports with valid TTO information, 55.0% of carboplatin-associated reports and 59.7% of cisplatin-associated reports occurred within 0–30 days, and Weibull modeling supported an early-failure reporting pattern for both agents. These findings suggest that the first treatment cycle and early treatment period may represent a particularly important window for pharmacovigilance-oriented monitoring in patients receiving platinum-based therapy for lung cancer.

From a clinical translation perspective, these findings may help refine organ-specific safety surveillance rather than directly determine platinum selection. For carboplatin-containing regimens, early attention to complete blood counts, infection symptoms, bone marrow reserve, hypersensitivity reactions, and liver-related laboratory abnormalities may be warranted. For cisplatin-containing regimens, closer monitoring of renal function, electrolytes, hydration status, thrombotic or cardiovascular symptoms, and auditory or vestibular complaints may be particularly relevant. These signal patterns could also inform the development of electronic health record–based alerts, structured adverse-event monitoring checklists, and prospective risk-stratified surveillance protocols in thoracic oncology practice.

Nevertheless, the findings should be interpreted as pharmacovigilance safety signals and reporting patterns rather than estimates of incidence, absolute risk, or causal toxicity. Confounding by indication, channeling bias, concomitant therapies, missing clinical covariates, and incomplete reporting remain important limitations. Therefore, the proposed monitoring implications should be regarded as hypothesis-generating and should be validated in prospective cohorts, electronic health record–based studies, or clinical trial datasets with detailed information on treatment regimen, cumulative dose, renal function, performance status, comorbidities, and supportive care.

## Data Availability

The datasets analyzed during the current study are available from the corresponding authors upon reasonable request. The raw FAERS data are publicly available from the FDA website (http://www.fda.gov).

## Glossary

FAERS: FDA Adverse Event Reporting System
ROR: Reporting odds ratio
PRR: Proportional reporting ratio
SOC: System Organ Class
CI: Confidence interval
START_DT: Therapy start date
EVENT_DT: Adverse event onset date
DEMO: Demographic file
DRUG: Drug file
REAC: Reaction file
PT: Preferred Term
PS: Primary suspect
AE: Adverse event
BCPNN: Bayesian Confidence Propagation Neural Network
MGPS: Multi-item Gamma Poisson Shrinker
CASEID: Case identifier
FDA: Food and Drug Administration
FDA_DT: FDA receipt date
PRES: posterior reversible encephalopathy syndrome
PRIMARYID: Primary report identifier
MedDRA: Medical Dictionary for Regulatory Activities
NSCLC: Non-small cell lung cancer
SCLC: Small cell lung cancer
IC: Information component
IC025: 95% CI lower bound of IC
EBGM: Empirical bayes geometric mean
EBGM05: 95% CI lower bound of EBGM
IQR: Interquartile range
TTO: Time-to-onset

## References

[1] Sung H Ferlay J Siegel RL Laversanne M Soerjomataram I Jemal A . Global Cancer statistics 2020: GLOBOCAN estimates of incidence and mortality worldwide for 36 cancers in 185 countries. CA Cancer J Clin. (2021) 71:209–49. doi: 10.3322/caac.21660, 33538338

[2] Riely GJ Wood DE Ettinger DS Aisner DL Akerley W Bauman JR . Non-small cell lung Cancer, version 4.2024, NCCN clinical practice guidelines in oncology. J Natl Compr Cancer Netw. (2024) 22:249–74. doi: 10.6004/jnccn.2204.0023, 38754467

[3] Zugazagoitia J Paz-Ares L. Extensive-stage small-cell lung cancer: first-line and second-line treatment options. J Clin Oncol. (2022) 40:671–80. doi: 10.1200/jco.21.01881, 34985925

[4] Wu Y Arroyo-Currás N. Nucleic acid-based electrochemical sensors facilitate the study of DNA binding by platinum (II)-based Antineoplastics. Angew Chem Int Ed Engl. (2024) 63:e202312402. doi: 10.1002/anie.202312402, 38227790 PMC10939885

[5] Duan M Leng S Mao P. Cisplatin in the era of PARP inhibitors and immunotherapy. Pharmacol Ther. (2024) 258:108642. doi: 10.1016/j.pharmthera.2024.108642, 38614254 PMC12231147

[6] Schoch S Gajewski S Rothfuß J Hartwig A Köberle B. Comparative study of the mode of action of clinically approved platinum-based chemotherapeutics. Int J Mol Sci. (2020) 21:6928. doi: 10.3390/ijms21186928, 32967255 PMC7555145

[7] Hah SS Stivers KM de Vere White RW Henderson PT. Kinetics of carboplatin-DNA binding in genomic DNA and bladder cancer cells as determined by accelerator mass spectrometry. Chem Res Toxicol. (2006) 19:622–6. doi: 10.1021/tx060058c, 16696564

[8] Lyrio R Rocha BRA Corrêa A Mascarenhas MGS Santos FL Maia RDH . Chemotherapy-induced acute kidney injury: epidemiology, pathophysiology, and therapeutic approaches. Front Nephrol. (2024) 4:1436896. doi: 10.3389/fneph.2024.1436896, 39185276 PMC11341478

[9] Bin Sharif MR Alam A Iqbal HMN Ayub SBA Habibur Rahaman M. Comparative efficacy and cycle-wise toxicity of paclitaxel-carboplatin versus gemcitabine-cisplatin in stage IV non-small cell lung cancer (NSCLC): a quasi-experimental study in Bangladesh. Cureus. (2025) 17:e93093. doi: 10.7759/cureus.93093, 41141108 PMC12550877

[10] Mell LK Torres-Saavedra PA Wong SJ Kish JA Chang SS Jordan RC . Radiotherapy with cetuximab or durvalumab for locoregionally advanced head and neck cancer in patients with a contraindication to cisplatin (NRG-HN004): an open-label, multicentre, parallel-group, randomised, phase 2/3 trial. Lancet Oncol. (2024) 25:1576–88. doi: 10.1016/s1470-2045(24)00507-2, 39551064 PMC11726348

[11] Furubayashi N Tsujita J Takayama A Nakashima S Nakamura M Negishi T. Clinical outcomes of Avelumab maintenance following full-dose, reduced-dose cisplatin, or carboplatin-based chemotherapy in advanced urothelial carcinoma. Res Rep Urol. (2025) 17:341–51. doi: 10.2147/rru.S554029, 41030367 PMC12478201

[12] Kadambi A Krekels EHJ Martinecz A Fatzinger G Jones HM Khachatryan A . Critical roles for modeling and simulation and real-world evidence to inform challenges in clinical trial diversity planning. Clin Transl Sci. (2025) 18:e70276. doi: 10.1111/cts.70276, 40525392 PMC12171783

[13] Wilson BE Booth CM. Real-world data: bridging the gap between clinical trials and practice. EClinicalMedicine. (2024) 78:102915. doi: 10.1016/j.eclinm.2024.102915, 39588211 PMC11585814

[14] Feng G Zhou X Chen J Li D Chen L. Platinum drugs-related safety profile: the latest five-year analysis from FDA adverse event reporting system data. Front Oncol. (2022) 12:1012093. doi: 10.3389/fonc.2022.1012093, 36713566 PMC9875054

[15] Song B Zhang Y Wang L Hu J. Comprehensive pharmacovigilance of phytoalkaloid chemotherapeutics: signal detection and time-to-onset analysis based on FAERS. Medicine (Baltimore). (2025) 104:e46044. doi: 10.1097/md.0000000000046044, 41305724 PMC12643699

[16] Wang L Yang K Zhao H Lv P Cai C Wang Z . Real-world safety of carboplatin in non-small cell lung cancer: a retrospective signal detection and subgroup analysis based on the FAERS database. Front Med (Lausanne). (2025) 12:1590738. doi: 10.3389/fmed.2025.1590738, 40589971 PMC12206876

[17] Potter E Reyes M Naples J Dal Pan G. FDA adverse event reporting system (FAERS) essentials: a guide to understanding, applying, and interpreting adverse event data reported to FAERS. Clin Pharmacol Ther. (2025) 118:567–82. doi: 10.1002/cpt.3701, 40384638 PMC12393772

[18] Chen JH Chen JC Mei YJ Zhu XC Luo QM Tu YY. Risk factors for drug-related impaired gastric emptying: a pharmacovigilance analysis of the FDA adverse event reporting system. Front Pharmacol. (2026) 17:1844057. doi: 10.3389/fphar.2026.1844057, 42232176 PMC13223089

[19] Yang F Xu L Lang X Feng X. A real-world safety signal detection study of ondansetron based on FAERS reports from 2014 to 2024. Sci Rep. (2025) 15:31339. doi: 10.1038/s41598-025-16384-9, 40858694 PMC12381205

[20] Wen H Dong Y Wang R. Spectrum and signals of medication-associated cognitive disorder: a comprehensive disproportionality analysis with cross-database validation. Front Pharmacol. (2026) 17:1762761. doi: 10.3389/fphar.2026.1762761, 42038310 PMC13106381

[21] Wang H Li X Shangguan L. Disproportionality analysis of drug-associated progressive multifocal leukoencephalopathy using spontaneous reports: a 20-year signal detection study based on the FAERS database. PLoS One. (2026) 21:e0341855. doi: 10.1371/journal.pone.0341855, 41632714 PMC12867222

[22] Yu X Zhong J Lin Z Fu H Zhang Y. Post-marketing safety concerns with trofinetide: a disproportionality analysis of the first therapeutic agent for Rett syndrome based on the FDA adverse event reporting system (FAERS). Front Pharmacol. (2026) 17:1643906. doi: 10.3389/fphar.2026.1643906, 41613782 PMC12847357

[23] Liu Q Ma W Hu C Fang C Zhong Y Wen J. Data mining and analysis of adverse drug events of propofol in the general population and the elderly based on the US Food and Drug Administration adverse event reporting system. Medicine (Baltimore). (2026) 105:e47947. doi: 10.1097/md.0000000000047947, 41790687 PMC12975220

[24] Shi Y Guan S Wang S Li M Yu Y Liu J . A disproportionality analysis of FDA adverse event reporting system (FAERS) events for filgotinib. PLoS One. (2025) 20:e0338188. doi: 10.1371/journal.pone.0338188, 41396990 PMC12704897

[25] Cerbito E Diab MI Alhoshani A Maayah ZH. Signal mining and safety profile analysis of lapatinib: a pharmacovigilance analysis of the FDA adverse event reporting system (FAERS) database. J Pharm Policy Pract. (2026) 19:2611182. doi: 10.1080/20523211.2025.2611182, 41541222 PMC12802520

[26] Sakaeda T Kadoyama K Okuno Y. Adverse event profiles of platinum agents: data mining of the public version of the FDA adverse event reporting system, AERS, and reproducibility of clinical observations. Int J Med Sci. (2011) 8:487–91. doi: 10.7150/ijms.8.487, 21897761 PMC3167097

[27] Yu Y Hou X Xu K. A multi-database pharmacovigilance study reveals distinctive immunosuppressive and opportunistic infection disproportionality signals with bevacizumab and temozolomide combination therapy in glioblastoma. Front Med (Lausanne). (2026) 13:1773599. doi: 10.3389/fmed.2026.1773599, 41889512 PMC13012968

[28] Bai Y Liu H Meng H. Sex-specific reporting patterns and onset timing of immune-related adverse events associated with nivolumab and pembrolizumab: a dual-database pharmacovigilance analysis. Front Immunol. (2026) 17:1837640. doi: 10.3389/fimmu.2026.1837640, 42212151 PMC13212199

[29] Tang F He Y Ou W Yang N Bai X. A disproportionality analysis of adverse events associated with ertapenem using the FAERS database from 2004 to 2024. Sci Rep. (2025) 15:17301. doi: 10.1038/s41598-025-02359-3, 40389541 PMC12089491

[30] Lv L Wu X Ren Y Guo Y Wang H Li X. Postmarketing safety evaluation of pemetrexed using FAERS and JADER databases. Sci Rep. (2025) 15:18738. doi: 10.1038/s41598-025-02426-9, 40436917 PMC12119796

[31] Chen Q Ying S Qin J Zhang L. Optimization of treatment strategies for elderly patients with advanced non-small cell lung cancer. Front Oncol. (2024) 14:1384906. doi: 10.3389/fonc.2024.1384906, 39081714 PMC11286424

[32] Han J Yang Z Zhao H. Lung cancer immunotherapy in 2025: where we stand and what comes next? Front Immunol. (2025) 16:1728163. doi: 10.3389/fimmu.2025.1728163, 41624891 PMC12855455

[33] Crawford J Herndon D Gmitter K Weiss J. The impact of myelosuppression on quality of life of patients treated with chemotherapy. Future Oncol. (2024) 20:1515–30. doi: 10.2217/fon-2023-0513, 38587388 PMC11441072

[34] Nwadibia JA Fasogbon IV Musyoka AM Ekpono EU Ibiam UA Orji OU . Protective effect of Ficus capensis lyophilized extract against carboplatin-induced liver injury via inhibition of oxidative stress and inflammation in rats. Toxicol Rep. (2024) 13:101734. doi: 10.1016/j.toxrep.2024.101734, 39328341 PMC11426155

[35] Raza M Manje Gowda A. "Platinum hypersensitivity and toxicity". In: StatPearls. Treasure Island (FL): StatPearls Publishing (2026). 40811600

[36] Russo C Ma L Johnson WR Singaraju R. Cisplatin-induced acute kidney injury and renal salt wasting syndrome. Fed Pract. (2021) 38:606–11a. doi: 10.12788/fp.0198, 35177891 PMC8843008

[37] Latcha S Chughtai S. Renal salt wasting following cisplatin. Kidney360. (2024) 5:604–6. doi: 10.34067/kid.0000000000000394, 38353669 PMC11093533

[38] Zhu YY Li Y Zeng L Li N. The risk factors for chemotherapy myelosuppression in breast cancer: a systematic review and meta-analysis. Front Genet. (2025) 16:1704489. doi: 10.3389/fgene.2025.1704489, 41438123 PMC12719511

[39] Triplett JD Kutlubaev MA Kermode AG Hardy T. Posterior reversible encephalopathy syndrome (PRES): diagnosis and management. Pract Neurol. (2022) 22:183–9. doi: 10.1136/practneurol-2021-003194, 35046115

[40] Marcu LG. Gender and sex-related differences in Normal tissue effects induced by platinum compounds. Pharmaceuticals (Basel). (2022) 15:255. doi: 10.3390/ph15020255, 35215367 PMC8876358

[41] Eshraghi-Jazi F Nematbakhsh M. Sex difference in cisplatin-induced nephrotoxicity: laboratory and clinical findings. J Toxicol. (2022) 2022:3507721. doi: 10.1155/2022/3507721, 36263084 PMC9576433

[42] Tolu-Akinnawo O Ogunniyi KE. Accelerated coronary artery disease in a patient with advanced lung cancer: interplay of cancer, inflammation, and treatment effects. Cureus. (2025) 17:e77769. doi: 10.7759/cureus.77769, 39981449 PMC11841481

[43] Zhao D Zhang W Liu Y Yan Z. Post-marketing safety concerns with lumateperone: a pharmacovigilance analysis based on the FDA adverse event reporting system (FAERS) database. Front Pharmacol. (2024) 15:1389814. doi: 10.3389/fphar.2024.1389814, 38783948 PMC11111848

[44] Huang LT Cao R Wang YR Sun L Zhang XY Guo YJ . Clinical option of pemetrexed-based versus paclitaxel-based first-line chemotherapeutic regimens in combination with bevacizumab for advanced non-squamous non-small-cell lung cancer and optimal maintenance therapy: evidence from a meta-analysis of randomized control trials. BMC Cancer. (2021) 21:426. doi: 10.1186/s12885-021-08136-5, 33865364 PMC8052669

