# Supplementary material for: Comparative safety signals of carboplatin and cisplatin in lung cancer: a pharmacovigilance study based on FAERS
Source: Front Med (Lausanne). 2026 Jul 22;13:1846025. doi: 10.3389/fmed.2026.1846025 (PMC13438454; doi:10.3389/fmed.2026.1846025)
Supplement: Supplementary file 1 [file Table_1.DOCX]

Supplementary Table S1

2×2 Contingency Table for Disproportionality Analysis

|  | **Target AEs** | **Other AEs** | **Total** |
| --- | --- | --- | --- |
| Target population | a | b | a+b |
| Other Populations | c | d | c+d |
| Total | a+c | b+d | a+b+c+d |

Supplementary Table S2

2×2 Contingency Table for Disproportionality Analysis and Signal Criteria

| **Methods** | **Formulas** | **Criteria** |
| --- | --- | --- |
| ROR | $ROR=\frac{ad}{bc}$ | *lower limit of 95% CI > 1, N ≥ 3* |
|  | $95\% CI=exp[\ln(ROR)\pm1.96\sqrt{\frac{1}{a}+\frac{1}{b}+\frac{1}{c}+\frac{1}{d}}]$ |  |
| PRR | $PRR=\frac{a/(a+b)}{c/(c+d)}$ | *PRR≥2,* $\chi^{2}$*≥ 4, N ≥ 3* |
|  | $\chi^{2}=\frac{(ad-bc)^{2}(a+b+c+d)}{(a+b)(c+d)(a+c)(b+d)}$ |  |
| BCPNN | $IC=\log_{2} [\frac{a(a+b+c+d)}{(a+c)(a+b)}]$ | *IC025 > 0* |
|  | $95\%CI=E(IC)\pm2\sqrt{V(IC)}$ |  |
| MGPS | $EBGM=\frac{a(a+b+c+d)}{(a+c)(a+b)}$ | *EBGM05 > 2* |
|  | $95\%CI=exp[\ln(EBGM)\pm1.96\sqrt{\frac{1}{a}+\frac{1}{b}+\frac{1}{c}+\frac{1}{d}}]$ |  |

Supplementary Table S3

Disproportionality analysis of System Organ Classes (SOCs) associated with carboplatin in the FAERS database

| Carboplatin SOCs | | | | | |
| --- | --- | --- | --- | --- | --- |
| SOCs | N | ROR (95%CI) | PRR(χ2) | EBGM(EBGM05) | IC(IC025) |
| General disorders and administration site conditions | 3986 | 0.79 (0.76–0.81) | 0.81 (192.27) | 0.82 (0.8) | -0.28 (-0.33) |
| Blood and lymphatic system disorders | 3736 | 2.88 (2.78–2.99) | 2.65 (3438.97) | 2.4 (2.32) | 1.26 (1.21) |
| Gastrointestinal disorders | 3145 | 0.92 (0.89–0.96) | 0.93 (18.73) | 0.93 (0.9) | -0.1 (-0.16) |
| Respiratory, thoracic and mediastinal disorders | 2484 | 0.9 (0.86–0.94) | 0.91 (24.31) | 0.91 (0.87) | -0.13 (-0.19) |
| Infections and infestations | 2270 | 1.38 (1.32–1.44) | 1.35 (203.23) | 1.32 (1.27) | 0.4 (0.34) |
| Skin and subcutaneous tissue disorders | 1725 | 0.8 (0.76–0.84) | 0.81 (74.02) | 0.82 (0.78) | -0.28 (-0.35) |
| Investigations | 1661 | 0.76 (0.72–0.8) | 0.77 (117.54) | 0.78 (0.74) | -0.36 (-0.43) |
| Nervous system disorders | 1574 | 0.9 (0.85–0.95) | 0.91 (15.49) | 0.91 (0.86) | -0.13 (-0.21) |
| Neoplasms benign, malignant and unspecified (incl cysts and polyps) | 1464 | 0.67 (0.63–0.7) | 0.68 (221.62) | 0.7 (0.66) | -0.52 (-0.6) |
| Injury, poisoning and procedural complications | 1326 | 0.96 (0.91–1.02) | 0.96 (2.06) | 0.96 (0.91) | -0.05 (-0.14) |
| Metabolism and nutrition disorders | 1150 | 1.04 (0.98–1.11) | 1.04 (1.96) | 1.04 (0.98) | 0.06 (-0.03) |
| Renal and urinary disorders | 1032 | 1.56 (1.46–1.66) | 1.54 (179.69) | 1.49 (1.39) | 0.57 (0.48) |
| Cardiac disorders | 870 | 1.03 (0.96–1.11) | 1.03 (0.84) | 1.03 (0.96) | 0.04 (-0.06) |
| Hepatobiliary disorders | 764 | 1.22 (1.13–1.31) | 1.21 (27.32) | 1.2 (1.11) | 0.26 (0.15) |
| Vascular disorders | 716 | 1.13 (1.04–1.22) | 1.12 (9.34) | 1.12 (1.03) | 0.16 (0.04) |
| Musculoskeletal and connective tissue disorders | 656 | 0.69 (0.64–0.75) | 0.7 (84.65) | 0.71 (0.66) | -0.49 (-0.61) |
| Endocrine disorders | 552 | 1.95 (1.78–2.13) | 1.93 (222.41) | 1.83 (1.67) | 0.87 (0.74) |
| Immune system disorders | 347 | 1.64 (1.46–1.83) | 1.63 (76.81) | 1.57 (1.4) | 0.65 (0.48) |
| Psychiatric disorders | 329 | 0.66 (0.59–0.74) | 0.67 (53.95) | 0.68 (0.61) | -0.56 (-0.72) |
| Eye disorders | 221 | 0.5 (0.43–0.57) | 0.5 (108) | 0.52 (0.45) | -0.95 (-1.15) |
| Congenital, familial and genetic disorders | 115 | 1.72 (1.42–2.08) | 1.72 (30.87) | 1.64 (1.35) | 0.72 (0.42) |
| Ear and labyrinth disorders | 94 | 1.05 (0.85–1.29) | 1.05 (0.19) | 1.04 (0.85) | 0.06 (-0.24) |
| Surgical and medical procedures | 81 | 0.54 (0.43–0.67) | 0.54 (30.84) | 0.56 (0.45) | -0.85 (-1.16) |
| Reproductive system and breast disorders | 22 | 0.4 (0.26–0.6) | 0.4 (19.72) | 0.41 (0.27) | -1.28 (-1.85) |
| Product issues | 19 | 0.44 (0.28–0.69) | 0.44 (13.4) | 0.45 (0.29) | -1.14 (-1.76) |
| Pregnancy, puerperium and perinatal conditions | 13 | 3.67 (2–6.74) | 3.67 (20.32) | 3.15 (1.72) | 1.65 (0.58) |
| Social circumstances | 9 | 0.22 (0.11–0.42) | 0.22 (25.15) | 0.23 (0.12) | -2.13 (-2.94) |

Supplementary Table S4

Disproportionality analysis of System Organ Classes (SOCs) associated with cisplatin in the FAERS database

| Cisplatin SOCs | | | | | |
| --- | --- | --- | --- | --- | --- |
| SOCs | N | ROR (95%CI) | PRR (χ2) | EBGM(EBGM05) | IC(IC025) |
| Blood and lymphatic system disorders | 706 | 2.9 (2.68–3.14) | 2.65 (740.27) | 2.6 (2.4) | 1.38 (1.26) |
| Gastrointestinal disorders | 692 | 1.2 (1.11–1.3) | 1.18 (20.32) | 1.17 (1.08) | 0.23 (0.11) |
| General disorders and administration site conditions | 564 | 0.62 (0.57–0.68) | 0.66 (112.95) | 0.67 (0.61) | -0.58 (-0.71) |
| Respiratory, thoracic and mediastinal disorders | 400 | 0.83 (0.75–0.92) | 0.84 (13.27) | 0.84 (0.76) | -0.25 (-0.4) |
| Infections and infestations | 393 | 1.34 (1.21–1.49) | 1.32 (31.27) | 1.31 (1.18) | 0.39 (0.24) |
| Nervous system disorders | 372 | 1.25 (1.13–1.39) | 1.24 (17.51) | 1.23 (1.11) | 0.3 (0.15) |
| Renal and urinary disorders | 267 | 2.3 (2.03–2.6) | 2.23 (181.4) | 2.2 (1.94) | 1.14 (0.95) |
| Vascular disorders | 251 | 2.33 (2.05–2.65) | 2.27 (177.75) | 2.24 (1.97) | 1.16 (0.97) |
| Metabolism and nutrition disorders | 250 | 1.31 (1.16–1.49) | 1.3 (17.56) | 1.29 (1.14) | 0.37 (0.18) |
| Investigations | 231 | 0.6 (0.53–0.69) | 0.62 (57.89) | 0.62 (0.54) | -0.69 (-0.88) |
| Neoplasms benign, malignant and unspecified (incl cysts and polyps) | 209 | 0.55 (0.48–0.63) | 0.57 (73.93) | 0.57 (0.5) | -0.81 (-1.01) |
| Cardiac disorders | 206 | 1.42 (1.23–1.63) | 1.4 (24.11) | 1.4 (1.21) | 0.48 (0.27) |
| Injury, poisoning and procedural complications | 203 | 0.84 (0.73–0.96) | 0.84 (6.16) | 0.84 (0.73) | -0.24 (-0.45) |
| Skin and subcutaneous tissue disorders | 170 | 0.44 (0.38–0.52) | 0.46 (113.57) | 0.47 (0.4) | -1.1 (-1.32) |
| Musculoskeletal and connective tissue disorders | 85 | 0.52 (0.42–0.64) | 0.52 (37.49) | 0.53 (0.43) | -0.92 (-1.23) |
| Psychiatric disorders | 74 | 0.87 (0.69–1.1) | 0.87 (1.36) | 0.87 (0.69) | -0.19 (-0.53) |
| Ear and labyrinth disorders | 54 | 3.56 (2.71–4.67) | 3.53 (94.53) | 3.44 (2.61) | 1.78 (1.32) |
| Hepatobiliary disorders | 46 | 0.41 (0.3–0.54) | 0.41 (39.62) | 0.41 (0.31) | -1.28 (-1.68) |
| Eye disorders | 45 | 0.6 (0.44–0.8) | 0.6 (12.1) | 0.6 (0.45) | -0.73 (-1.15) |
| Immune system disorders | 34 | 0.88 (0.63–1.23) | 0.88 (0.57) | 0.88 (0.63) | -0.18 (-0.67) |
| Endocrine disorders | 26 | 0.49 (0.33–0.72) | 0.49 (13.88) | 0.49 (0.33) | -1.02 (-1.55) |
| Congenital, familial and genetic disorders | 8 | 0.65 (0.32–1.31) | 0.65 (1.48) | 0.65 (0.33) | -0.61 (-1.52) |
| Social circumstances | 6 | 0.87 (0.39–1.94) | 0.87 (0.12) | 0.87 (0.39) | -0.2 (-1.27) |
| Reproductive system and breast disorders | 6 | 0.64 (0.29–1.43) | 0.64 (1.2) | 0.64 (0.29) | -0.64 (-1.66) |
| Surgical and medical procedures | 3 | 0.12 (0.04–0.36) | 0.12 (20.13) | 0.12 (0.04) | -3.09 (-4.17) |
| Product issues | 1 | 0.14 (0.02–0.96) | 0.14 (5.53) | 0.14 (0.02) | -2.87 (-4.1) |
| Pregnancy, puerperium and perinatal conditions | 1 | 1.39 (0.19–10.03) | 1.39 (0.11) | 1.39 (0.19) | 0.47 (-1.86) |

Supplementary Table S5

All algorithmically detected positive carboplatin-associated Preferred Terms prior to clinical relevance filtering in the FAERS database

| All algorithmically detected positive carboplatin-associated Preferred Terms prior to clinical relevance filtering in the FAERS database (n=30,361) | | | | | |
| --- | --- | --- | --- | --- | --- |
| PT | N | ROR (95%CI) | PRR(χ^2^) | EBGM(EBGM05) | IC(IC025) |
| Anaemia | 627 | 2.43 (2.23–2.65) | 2.4 (445.5) | 2.21 (2.03) | 1.14 (1.01) |
| Neutropenia | 556 | 3.1 (2.83–3.4) | 3.06 (644.81) | 2.71 (2.47) | 1.44 (1.3) |
| Pancytopenia | 529 | 6.42 (5.8–7.11) | 6.33 (1674.58) | 4.75 (4.28) | 2.25 (2.09) |
| Thrombocytopenia | 508 | 3.12 (2.83–3.44) | 3.09 (597.41) | 2.73 (2.48) | 1.45 (1.3) |
| Febrile neutropenia | 470 | 2.96 (2.68–3.28) | 2.93 (504.14) | 2.62 (2.37) | 1.39 (1.24) |
| Acute kidney injury | 395 | 2.89 (2.59–3.22) | 2.86 (403.86) | 2.56 (2.3) | 1.36 (1.19) |
| Leukopenia | 349 | 4.85 (4.3–5.48) | 4.81 (799.36) | 3.88 (3.44) | 1.96 (1.77) |
| Septic shock | 167 | 3.67 (3.09–4.34) | 3.65 (258.97) | 3.13 (2.64) | 1.65 (1.38) |
| Condition aggravated | 165 | 2.88 (2.44–3.4) | 2.87 (169.11) | 2.57 (2.17) | 1.36 (1.1) |
| Mucosal inflammation | 157 | 2.96 (2.5–3.52) | 2.95 (169.85) | 2.63 (2.22) | 1.4 (1.13) |
| Neutropenic sepsis | 146 | 7.22 (5.93–8.8) | 7.19 (526.43) | 5.18 (4.25) | 2.37 (2.06) |
| Therapy partial responder | 127 | 3.87 (3.18–4.71) | 3.86 (214.12) | 3.27 (2.69) | 1.71 (1.4) |
| Polyneuropathy | 110 | 8.89 (7.02–11.25) | 8.86 (482.13) | 5.94 (4.69) | 2.57 (2.18) |
| Hyperthyroidism | 93 | 2.91 (2.33–3.63) | 2.9 (97.23) | 2.59 (2.08) | 1.37 (1.03) |
| Rash maculo-papular | 85 | 3.58 (2.83–4.54) | 3.58 (127.46) | 3.08 (2.43) | 1.62 (1.24) |
| Hepatic cytolysis | 85 | 3.4 (2.69–4.31) | 3.39 (117.19) | 2.95 (2.33) | 1.56 (1.19) |
| Agranulocytosis | 83 | 4.7 (3.67–6.02) | 4.69 (183.7) | 3.81 (2.98) | 1.93 (1.53) |
| Anaphylactic shock | 81 | 4.71 (3.67–6.05) | 4.7 (179.83) | 3.82 (2.97) | 1.93 (1.52) |
| Hypomagnesaemia | 80 | 3.59 (2.82–4.59) | 3.59 (120.55) | 3.09 (2.42) | 1.63 (1.24) |
| Disease recurrence | 74 | 9.83 (7.33–13.18) | 9.81 (353.87) | 6.32 (4.72) | 2.66 (2.16) |
| Therapeutic product effect incomplete | 70 | 5.2 (3.96–6.83) | 5.19 (175.97) | 4.11 (3.13) | 2.04 (1.59) |
| Febrile bone marrow aplasia | 66 | 6.52 (4.88–8.7) | 6.5 (214.42) | 4.84 (3.62) | 2.27 (1.78) |
| Hypertransaminasaemia | 63 | 5.22 (3.92–6.96) | 5.21 (159.21) | 4.13 (3.1) | 2.04 (1.56) |
| Hepatocellular injury | 57 | 3.43 (2.57–4.58) | 3.43 (79.84) | 2.98 (2.23) | 1.57 (1.11) |
| Therapy non-responder | 56 | 3.67 (2.74–4.91) | 3.66 (87.15) | 3.14 (2.34) | 1.65 (1.17) |
| Toxic epidermal necrolysis | 50 | 3.62 (2.66–4.93) | 3.62 (76.33) | 3.11 (2.28) | 1.64 (1.13) |
| Posterior reversible encephalopathy syndrome | 47 | 4.55 (3.28–6.3) | 4.54 (99.65) | 3.72 (2.68) | 1.89 (1.34) |
| Acute myeloid leukaemia | 46 | 5.23 (3.74–7.31) | 5.22 (116.41) | 4.13 (2.95) | 2.05 (1.47) |
| Immune-mediated hypothyroidism | 43 | 5.81 (4.08–8.26) | 5.8 (123.23) | 4.46 (3.14) | 2.16 (1.55) |
| Lymphopenia | 42 | 3.82 (2.72–5.36) | 3.81 (69.49) | 3.24 (2.31) | 1.7 (1.13) |
| Toxic skin eruption | 39 | 3.61 (2.54–5.12) | 3.61 (59.22) | 3.1 (2.19) | 1.63 (1.05) |
| Autoimmune hepatitis | 37 | 3.45 (2.41–4.93) | 3.44 (52.15) | 2.99 (2.09) | 1.58 (0.99) |
| Bicytopenia | 35 | 8.6 (5.68–13.04) | 8.59 (149.26) | 5.83 (3.84) | 2.54 (1.78) |
| Myelodysplastic syndrome | 34 | 4.85 (3.3–7.15) | 4.85 (78.52) | 3.91 (2.65) | 1.97 (1.3) |
| Tumour lysis syndrome | 30 | 3.84 (2.57–5.74) | 3.84 (50.18) | 3.26 (2.18) | 1.71 (1.02) |
| Prescribed overdose | 30 | 4.24 (2.83–6.36) | 4.24 (57.89) | 3.52 (2.35) | 1.82 (1.12) |
| Congenital aplasia | 30 | 10.71 (6.7–17.11) | 10.7 (153.88) | 6.66 (4.17) | 2.73 (1.86) |
| Therapeutic response decreased | 30 | 3.6 (2.41–5.36) | 3.59 (45.33) | 3.09 (2.08) | 1.63 (0.96) |
| Angioedema | 27 | 4.22 (2.75–6.46) | 4.21 (51.64) | 3.51 (2.29) | 1.81 (1.07) |
| Acute coronary syndrome | 27 | 3.85 (2.52–5.88) | 3.85 (45.35) | 3.27 (2.14) | 1.71 (0.99) |
| Dermatitis exfoliative generalised | 27 | 7.23 (4.57–11.44) | 7.22 (97.66) | 5.2 (3.28) | 2.38 (1.53) |
| Renal tubular disorder | 26 | 6 (3.8–9.45) | 5.99 (77.24) | 4.57 (2.9) | 2.19 (1.37) |
| Dermatitis bullous | 26 | 3.78 (2.46–5.82) | 3.78 (42.47) | 3.22 (2.09) | 1.69 (0.95) |
| Neutropenic colitis | 26 | 6.72 (4.23–10.67) | 6.71 (87.32) | 4.95 (3.11) | 2.31 (1.46) |
| Immune-mediated hyperthyroidism | 23 | 15.67 (8.73–28.12) | 15.66 (154.32) | 8.17 (4.55) | 3.03 (1.9) |
| Squamous cell carcinoma of lung | 23 | 5.22 (3.25–8.4) | 5.22 (58.19) | 4.13 (2.57) | 2.05 (1.19) |
| Autoimmune haemolytic anaemia | 22 | 5 (3.08–8.09) | 4.99 (52.69) | 3.99 (2.47) | 2 (1.13) |
| Endocarditis | 20 | 7.14 (4.19–12.16) | 7.13 (71.44) | 5.15 (3.03) | 2.37 (1.36) |
| Autoimmune colitis | 20 | 4 (2.44–6.55) | 3.99 (35.45) | 3.36 (2.05) | 1.75 (0.89) |
| Pulmonary sepsis | 19 | 5.37 (3.18–9.07) | 5.37 (49.74) | 4.22 (2.5) | 2.08 (1.12) |
| Contraindicated product administered | 18 | 14.2 (7.45–27.05) | 14.19 (113.33) | 7.77 (4.08) | 2.96 (1.68) |
| Acute generalised exanthematous pustulosis | 18 | 4.82 (2.83–8.19) | 4.81 (41.17) | 3.89 (2.28) | 1.96 (1) |
| Lip oedema | 16 | 11.99 (6.21–23.14) | 11.98 (89.48) | 7.1 (3.68) | 2.83 (1.51) |
| Extremity necrosis | 16 | 4.36 (2.5–7.61) | 4.36 (32.07) | 3.6 (2.06) | 1.85 (0.85) |
| Immune-mediated hypophysitis | 15 | 7.02 (3.8–12.97) | 7.02 (52.74) | 5.1 (2.76) | 2.35 (1.17) |
| Raynaud's phenomenon | 14 | 6.77 (3.6–12.72) | 6.76 (47.38) | 4.97 (2.64) | 2.31 (1.09) |
| Febrile infection | 14 | 5.83 (3.14–10.8) | 5.82 (40.29) | 4.47 (2.41) | 2.16 (0.99) |
| Myelitis transverse | 13 | 7.79 (3.99–15.23) | 7.79 (50.61) | 5.47 (2.8) | 2.45 (1.13) |
| Jugular vein thrombosis | 13 | 5.13 (2.73–9.62) | 5.12 (32.15) | 4.07 (2.17) | 2.03 (0.85) |
| Papilloedema | 12 | 5.45 (2.81–10.55) | 5.45 (31.95) | 4.26 (2.2) | 2.09 (0.84) |
| Demyelinating polyneuropathy | 12 | 7.82 (3.89–15.71) | 7.81 (46.86) | 5.48 (2.73) | 2.45 (1.06) |
| Progressive multifocal leukoencephalopathy | 12 | 5.8 (2.98–11.29) | 5.8 (34.35) | 4.46 (2.29) | 2.16 (0.88) |
| Cutaneous lupus erythematosus | 11 | 6.59 (3.24–13.4) | 6.59 (36.23) | 4.88 (2.4) | 2.29 (0.9) |
| Peripheral sensorimotor neuropathy | 11 | 7.17 (3.49–14.7) | 7.16 (39.46) | 5.17 (2.52) | 2.37 (0.95) |
| Autoimmune hypothyroidism | 11 | 8.67 (4.13–18.23) | 8.67 (47.28) | 5.86 (2.79) | 2.55 (1.05) |
| Tracheo-oesophageal fistula | 11 | 5.32 (2.67–10.58) | 5.31 (28.44) | 4.18 (2.1) | 2.07 (0.76) |
| Troponin t increased | 10 | 5.99 (2.88–12.48) | 5.99 (29.7) | 4.57 (2.19) | 2.19 (0.76) |
| Optic ischaemic neuropathy | 10 | 8.81 (4.04–19.25) | 8.81 (43.6) | 5.92 (2.71) | 2.57 (0.97) |
| Large intestine infection | 10 | 8.81 (4.04–19.25) | 8.81 (43.6) | 5.92 (2.71) | 2.57 (0.97) |
| Vascular purpura | 9 | 8.99 (3.93–20.54) | 8.99 (39.93) | 5.99 (2.62) | 2.58 (0.88) |
| Palmar erythema | 9 | 6.13 (2.82–13.31) | 6.13 (27.41) | 4.64 (2.14) | 2.21 (0.69) |
| Infusion site erythema | 9 | 9.63 (4.17–22.25) | 9.63 (42.36) | 6.25 (2.71) | 2.64 (0.91) |
| Necrotising myositis | 9 | 10.37 (4.43–24.27) | 10.37 (45.02) | 6.54 (2.79) | 2.71 (0.94) |
| Anaemia of malignant disease | 9 | 11.24 (4.73–26.67) | 11.23 (47.95) | 6.85 (2.89) | 2.78 (0.97) |
| Magnetic resonance imaging abnormal | 9 | 13.48 (5.48–33.19) | 13.48 (54.73) | 7.57 (3.08) | 2.92 (1.03) |
| Vascular device infection | 9 | 8.99 (3.93–20.54) | 8.99 (39.93) | 5.99 (2.62) | 2.58 (0.88) |
| Oesophagitis ulcerative | 8 | 7.49 (3.21–17.5) | 7.49 (29.99) | 5.33 (2.28) | 2.41 (0.69) |
| Intestinal pseudo-obstruction | 8 | 9.99 (4.08–24.44) | 9.99 (38.81) | 6.39 (2.61) | 2.68 (0.81) |
| Exposure during pregnancy | 8 | 10.9 (4.38–27.09) | 10.89 (41.62) | 6.73 (2.71) | 2.75 (0.84) |
| Glucocorticoid deficiency | 8 | 7.05 (3.04–16.34) | 7.05 (28.24) | 5.11 (2.21) | 2.35 (0.66) |
| Herpes simplex reactivation | 8 | 19.98 (6.93–57.58) | 19.97 (61.79) | 9.13 (3.17) | 3.19 (1) |
| Clonus | 7 | 14.98 (5.25–42.71) | 14.98 (45.66) | 7.99 (2.8) | 3 (0.78) |
| Endophthalmitis | 7 | 26.22 (7.67–89.57) | 26.21 (61.73) | 10.17 (2.98) | 3.35 (0.87) |
| Actinic keratosis | 7 | 10.49 (3.99–27.55) | 10.48 (35.33) | 6.58 (2.5) | 2.72 (0.69) |
| Terminal agitation | 7 | 52.43 (10.89–252.42) | 52.42 (78.47) | 12.43 (2.58) | 3.64 (0.93) |
| Myeloproliferative neoplasm | 7 | 10.49 (3.99–27.55) | 10.48 (35.33) | 6.58 (2.5) | 2.72 (0.69) |
| Gastroenteritis norovirus | 7 | 52.43 (10.89–252.42) | 52.42 (78.47) | 12.43 (2.58) | 3.64 (0.93) |
| Chronic myeloid leukaemia | 7 | 8.07 (3.22–20.22) | 8.07 (28.16) | 5.59 (2.23) | 2.48 (0.59) |
| Vitritis | 6 | 29.96 (7.49–119.81) | 29.96 (55.98) | 10.65 (2.66) | 3.41 (0.68) |
| Shock symptom | 6 | 9.99 (3.55–28.06) | 9.99 (29.11) | 6.39 (2.27) | 2.68 (0.5) |
| Fanconi syndrome | 6 | 14.98 (4.83–46.45) | 14.98 (39.14) | 7.99 (2.58) | 3 (0.6) |
| Hyperphosphaturia | 6 | 22.47 (6.34–79.64) | 22.47 (49.23) | 9.59 (2.71) | 3.26 (0.66) |
| Pseudomonal sepsis | 6 | 8.99 (3.27–24.73) | 8.99 (26.62) | 5.99 (2.18) | 2.58 (0.47) |
| Haemorrhagic disorder | 6 | 8.99 (3.27–24.73) | 8.99 (26.62) | 5.99 (2.18) | 2.58 (0.47) |
| Paraneoplastic rash | 6 | 29.96 (7.49–119.81) | 29.96 (55.98) | 10.65 (2.66) | 3.41 (0.68) |
| Antinuclear antibody | 6 | 29.96 (7.49–119.81) | 29.96 (55.98) | 10.65 (2.66) | 3.41 (0.68) |
| Genital tract inflammation | 6 | 44.94 (9.07–222.69) | 44.93 (64.44) | 11.98 (2.42) | 3.58 (0.71) |
| Lung carcinoma cell type unspecified stage iii | 6 | 29.96 (7.49–119.81) | 29.96 (55.98) | 10.65 (2.66) | 3.41 (0.68) |
| Infusion related hypersensitivity reaction | 6 | 9.99 (3.55–28.06) | 9.99 (29.11) | 6.39 (2.27) | 2.68 (0.5) |
| Idiopathic intracranial hypertension | 6 | 14.98 (4.83–46.45) | 14.98 (39.14) | 7.99 (2.58) | 3 (0.6) |
| Blood phosphorus abnormal | 6 | 12.84 (4.32–38.21) | 12.84 (35.27) | 7.37 (2.48) | 2.88 (0.56) |
| Intensive care | 5 | 9.36 (3.06–28.62) | 9.36 (22.98) | 6.15 (2.01) | 2.62 (0.27) |
| Punctate keratitis | 5 | 24.97 (5.97–104.48) | 24.96 (43.13) | 9.99 (2.39) | 3.32 (0.42) |
| Fournier's gangrene | 5 | 18.73 (5.03–69.74) | 18.72 (37.28) | 8.88 (2.38) | 3.15 (0.39) |
| Occipital neuralgia | 5 | 18.73 (5.03–69.74) | 18.72 (37.28) | 8.88 (2.38) | 3.15 (0.39) |
| Hyperphosphataemia | 5 | 10.7 (3.4–33.72) | 10.7 (25.64) | 6.66 (2.11) | 2.73 (0.3) |
| Epidermolysis bullosa | 5 | 9.36 (3.06–28.62) | 9.36 (22.98) | 6.15 (2.01) | 2.62 (0.27) |
| Epiglottic oedema | 5 | 37.45 (7.27–193.05) | 37.44 (50.67) | 11.41 (2.21) | 3.51 (0.44) |
| Escherichia test positive | 5 | 9.36 (3.06–28.62) | 9.36 (22.98) | 6.15 (2.01) | 2.62 (0.27) |
| Subclavian artery occlusion | 5 | 24.97 (5.97–104.48) | 24.96 (43.13) | 9.99 (2.39) | 3.32 (0.42) |
| Chronic myelomonocytic leukaemia | 5 | 10.7 (3.4–33.72) | 10.7 (25.64) | 6.66 (2.11) | 2.73 (0.3) |
| Oligoastrocytoma | 4 | 19.97 (4.47–89.25) | 19.97 (30.89) | 9.13 (2.04) | 3.19 (0.09) |
| Myopathy toxic | 4 | 19.97 (4.47–89.25) | 19.97 (30.89) | 9.13 (2.04) | 3.19 (0.09) |
| Fusarium infection | 4 | 19.97 (4.47–89.25) | 19.97 (30.89) | 9.13 (2.04) | 3.19 (0.09) |

Supplementary Table S6

All algorithmically detected positive cisplatin-associated Preferred Terms prior to clinical relevance filtering in the FAERS database

| All algorithmically detected positive cisplatin-associated Preferred Terms prior to clinical relevance filtering in the FAERS database(n=5,303) | | | | | |
| --- | --- | --- | --- | --- | --- |
| PT | N | ROR (95%CI) | PRR(χ^2^) | EBGM(EBGM05) | IC(IC025) |
| Neutropenia | 144 | 4.25 (3.59–5.03) | 4.16 (332.53) | 4.02 (3.39) | 2.01 (1.73) |
| Febrile neutropenia | 106 | 3.52 (2.89–4.28) | 3.47 (180.65) | 3.38 (2.78) | 1.76 (1.44) |
| Acute kidney injury | 98 | 3.8 (3.1–4.66) | 3.75 (190.8) | 3.64 (2.97) | 1.86 (1.53) |
| Thrombocytopenia | 83 | 2.62 (2.11–3.27) | 2.6 (79.77) | 2.55 (2.05) | 1.35 (1) |
| Pancytopenia | 73 | 3.91 (3.09–4.95) | 3.87 (149.36) | 3.75 (2.96) | 1.91 (1.51) |
| Pulmonary embolism | 65 | 3.08 (2.4–3.95) | 3.06 (87.46) | 2.99 (2.33) | 1.58 (1.17) |
| Leukopenia | 63 | 4.19 (3.25–5.4) | 4.15 (144.54) | 4.01 (3.11) | 2 (1.57) |
| Renal failure | 46 | 2.99 (2.23–4.02) | 2.97 (58.57) | 2.91 (2.17) | 1.54 (1.05) |
| Septic shock | 38 | 4.25 (3.06–5.89) | 4.22 (89.49) | 4.08 (2.94) | 2.03 (1.44) |
| Neutropenic sepsis | 33 | 7.2 (5.05–10.27) | 7.16 (162.22) | 6.71 (4.7) | 2.75 (2) |
| Toxicity to various agents | 30 | 3.62 (2.51–5.23) | 3.61 (54.51) | 3.51 (2.43) | 1.81 (1.17) |
| Oesophagitis | 29 | 5.2 (3.57–7.56) | 5.18 (92.5) | 4.95 (3.4) | 2.31 (1.58) |
| Aortic thrombosis | 29 | 75.37 (46.04–123.39) | 74.97 (1157.58) | 41.45 (25.32) | 5.37 (3.49) |
| Nephropathy toxic | 25 | 8.54 (5.66–12.88) | 8.5 (151.4) | 7.86 (5.21) | 2.97 (2.04) |
| Acute myocardial infarction | 24 | 7.05 (4.65–10.7) | 7.03 (115.21) | 6.59 (4.35) | 2.72 (1.83) |
| Bone marrow failure | 23 | 3.27 (2.15–4.96) | 3.26 (34.76) | 3.18 (2.09) | 1.67 (0.94) |
| Multiple organ dysfunction syndrome | 20 | 3.98 (2.54–6.23) | 3.97 (42.58) | 3.84 (2.45) | 1.94 (1.11) |
| Renal tubular necrosis | 19 | 9.48 (5.9–15.21) | 9.45 (129.97) | 8.65 (5.39) | 3.11 (1.96) |
| Hypomagnesaemia | 17 | 3.88 (2.39–6.32) | 3.87 (34.79) | 3.76 (2.31) | 1.91 (1) |
| Renal salt-wasting syndrome | 16 | 242 (94.66–618.69) | 241.27 (1044.17) | 66.53 (26.02) | 6.06 (2.84) |
| Gastrointestinal toxicity | 16 | 6.81 (4.1–11.33) | 6.8 (73.61) | 6.39 (3.84) | 2.68 (1.55) |
| Tinnitus | 15 | 6.39 (3.78–10.79) | 6.37 (63.49) | 6.02 (3.56) | 2.59 (1.44) |
| Cytomegalovirus oesophagitis | 15 | 170.12 (72.1–401.43) | 169.65 (874.74) | 59.66 (25.28) | 5.9 (2.73) |
| Inappropriate antidiuretic hormone secretion | 13 | 4.87 (2.79–8.51) | 4.86 (37.85) | 4.66 (2.67) | 2.22 (1.09) |
| Posterior reversible encephalopathy syndrome | 13 | 6.24 (3.55–10.95) | 6.22 (53.35) | 5.89 (3.35) | 2.56 (1.32) |
| Orthostatic hypotension | 12 | 8.18 (4.53–14.78) | 8.16 (69.21) | 7.57 (4.19) | 2.92 (1.49) |
| Aphasia | 11 | 3.91 (2.14–7.15) | 3.9 (22.78) | 3.78 (2.07) | 1.92 (0.76) |
| Hypoacusis | 11 | 3.88 (2.12–7.1) | 3.87 (22.49) | 3.75 (2.05) | 1.91 (0.75) |
| Renal infarct | 11 | 35.62 (17.72–71.58) | 35.54 (265.16) | 25.8 (12.84) | 4.69 (2.11) |
| Deafness | 10 | 3.96 (2.1–7.46) | 3.95 (21.13) | 3.83 (2.03) | 1.94 (0.71) |
| Arterial thrombosis | 10 | 19.29 (9.74–38.18) | 19.25 (142.69) | 16.05 (8.11) | 4 (1.8) |
| Capillary leak syndrome | 10 | 14.16 (7.27–27.59) | 14.14 (105.6) | 12.36 (6.34) | 3.63 (1.66) |
| Peripheral artery thrombosis | 10 | 25.18 (12.49–50.76) | 25.13 (181.37) | 19.89 (9.86) | 4.31 (1.89) |
| Febrile bone marrow aplasia | 10 | 4.36 (2.31–8.22) | 4.35 (24.63) | 4.2 (2.22) | 2.07 (0.8) |
| Cyanosis | 9 | 4.88 (2.5–9.55) | 4.88 (26.32) | 4.68 (2.39) | 2.23 (0.83) |
| Neurotoxicity | 9 | 4.5 (2.31–8.8) | 4.5 (23.34) | 4.33 (2.22) | 2.12 (0.76) |
| Presyncope | 9 | 9.27 (4.66–18.41) | 9.25 (60.12) | 8.49 (4.27) | 3.09 (1.31) |
| Enteritis | 9 | 4.41 (2.26–8.61) | 4.4 (22.57) | 4.24 (2.17) | 2.09 (0.74) |
| Melanoderma | 9 | 74.15 (30.71–179.02) | 74.03 (356.62) | 41.16 (17.05) | 5.36 (1.91) |
| Bicytopenia | 9 | 9.37 (4.72–18.63) | 9.36 (60.91) | 8.58 (4.32) | 3.1 (1.32) |
| Peripheral ischaemia | 9 | 7.03 (3.57–13.86) | 7.02 (43.13) | 6.59 (3.34) | 2.72 (1.12) |
| Arterial occlusive disease | 9 | 27.19 (12.9–57.29) | 27.14 (174.34) | 21.11 (10.02) | 4.4 (1.78) |
| Splenic infarction | 8 | 23.38 (10.74–50.89) | 23.35 (136.04) | 18.76 (8.62) | 4.23 (1.58) |
| Cerebral venous thrombosis | 8 | 65.9 (26.5–163.9) | 65.8 (295.59) | 38.52 (15.49) | 5.27 (1.73) |
| Metabolic acidosis | 7 | 5.2 (2.42–11.14) | 5.19 (22.41) | 4.96 (2.32) | 2.31 (0.67) |
| Empyema | 6 | 8.36 (3.62–19.3) | 8.35 (35.55) | 7.73 (3.35) | 2.95 (0.83) |
| Ototoxicity | 6 | 16.98 (7.1–40.63) | 16.96 (75.91) | 14.44 (6.04) | 3.85 (1.11) |
| Hepatitis b | 6 | 10.87 (4.66–25.36) | 10.86 (47.95) | 9.8 (4.2) | 3.29 (0.95) |
| Hypovolaemia | 6 | 5.97 (2.61–13.65) | 5.97 (23.27) | 5.66 (2.48) | 2.5 (0.63) |
| Ileus paralytic | 6 | 6.32 (2.76–14.46) | 6.31 (25.08) | 5.97 (2.61) | 2.58 (0.66) |
| Radiation oesophagitis | 6 | 6.88 (3–15.78) | 6.87 (27.98) | 6.46 (2.81) | 2.69 (0.72) |
| Type i hypersensitivity | 6 | 20.9 (8.6–50.8) | 20.88 (92.28) | 17.15 (7.06) | 4.1 (1.16) |
| Arteriospasm coronary | 6 | 12.94 (5.5–30.45) | 12.93 (57.77) | 11.43 (4.86) | 3.52 (1.02) |
| Neutropenic colitis | 6 | 6.97 (3.04–15.99) | 6.96 (28.44) | 6.53 (2.85) | 2.71 (0.72) |
| Splenic rupture | 5 | 45.28 (15.47–132.52) | 45.24 (144.21) | 30.49 (10.42) | 4.93 (0.97) |
| Intestinal ischaemia | 5 | 6.47 (2.61–16.03) | 6.46 (21.55) | 6.1 (2.46) | 2.61 (0.49) |
| Pulmonary nocardiosis | 5 | 64.69 (20.52–203.88) | 64.63 (182.71) | 38.12 (12.09) | 5.25 (0.97) |
| Hypoparathyroidism | 5 | 32.34 (11.65–89.83) | 32.31 (111.8) | 24.07 (8.67) | 4.59 (0.96) |
| Dilated cardiomyopathy | 5 | 10.53 (4.17–26.59) | 10.52 (38.6) | 9.53 (3.77) | 3.25 (0.72) |
| Hypercreatininaemia | 5 | 10.06 (3.99–25.36) | 10.05 (36.69) | 9.15 (3.63) | 3.19 (0.7) |
| Idiopathic pulmonary fibrosis | 5 | 9.43 (3.75–23.7) | 9.42 (34.11) | 8.63 (3.43) | 3.11 (0.67) |
| Tonsillitis | 4 | 20.12 (6.81–59.47) | 20.11 (59.42) | 16.63 (5.63) | 4.06 (0.57) |
| Kaposi's sarcoma | 4 | 90.55 (22.64–362.14) | 90.48 (176.98) | 45.74 (11.44) | 5.52 (0.58) |
| Diastolic dysfunction | 4 | 17.25 (5.92–50.26) | 17.23 (51.38) | 14.64 (5.02) | 3.87 (0.55) |
| Necrosis ischaemic | 4 | 362.18 (40.47–3241.09) | 361.91 (287.93) | 73.18 (8.18) | 6.19 (0.48) |
| Skin hyperpigmentation | 4 | 6.04 (2.19–16.61) | 6.03 (15.74) | 5.72 (2.08) | 2.52 (0.21) |
| Ulcer haemorrhage | 4 | 10.06 (3.58–28.27) | 10.05 (29.35) | 9.15 (3.25) | 3.19 (0.42) |
| Recall phenomenon | 4 | 9.05 (3.24–25.31) | 9.05 (26.03) | 8.32 (2.97) | 3.06 (0.38) |
| Embolism arterial | 4 | 6.96 (2.52–19.26) | 6.96 (18.96) | 6.53 (2.36) | 2.71 (0.28) |
| Subclavian vein thrombosis | 4 | 12.49 (4.39–35.54) | 12.48 (37.12) | 11.09 (3.9) | 3.47 (0.48) |
| Coronary artery thrombosis | 4 | 25.87 (8.51–78.62) | 25.85 (74.33) | 20.33 (6.69) | 4.35 (0.6) |
| Diabetes mellitus inadequate control | 4 | 6.47 (2.34–17.84) | 6.46 (17.24) | 6.1 (2.21) | 2.61 (0.24) |
| Drug monitoring procedure incorrectly performed | 4 | 362.18 (40.47–3241.09) | 361.91 (287.93) | 73.18 (8.18) | 6.19 (0.48) |
| Contraindicated product administered | 4 | 10.97 (3.89–30.99) | 10.97 (32.32) | 9.89 (3.5) | 3.31 (0.45) |
| Fibrosis | 3 | 12.93 (3.86–43.37) | 12.93 (28.88) | 11.43 (3.41) | 3.52 (0.1) |
| Salmonellosis | 3 | 67.9 (15.19–303.44) | 67.86 (112.93) | 39.2 (8.77) | 5.29 (0.11) |
| Myopericarditis | 3 | 12.34 (3.69–41.26) | 12.34 (27.51) | 10.98 (3.28) | 3.46 (0.09) |
| Formication | 3 | 16.97 (4.94–58.27) | 16.96 (37.96) | 14.44 (4.21) | 3.85 (0.13) |
| Haemoperitoneum | 3 | 20.89 (5.95–73.33) | 20.88 (46.14) | 17.15 (4.89) | 4.1 (0.15) |
| Tearfulness | 3 | 22.63 (6.38–80.22) | 22.62 (49.59) | 18.3 (5.16) | 4.19 (0.15) |
| Ophthalmoplegia | 3 | 13.58 (4.03–45.71) | 13.57 (30.38) | 11.93 (3.54) | 3.58 (0.11) |
| Ventricular dysfunction | 3 | 11.81 (3.54–39.34) | 11.8 (26.24) | 10.56 (3.17) | 3.4 (0.08) |
| Portal vein thrombosis | 3 | 9.05 (2.76–29.67) | 9.05 (19.52) | 8.32 (2.54) | 3.06 (0.02) |
| Hepatic infarction | 3 | 90.53 (18.27–448.64) | 90.48 (132.73) | 45.74 (9.23) | 5.52 (0.08) |
| Mallory-weiss syndrome | 3 | 15.09 (4.44–51.24) | 15.08 (33.81) | 13.07 (3.85) | 3.71 (0.12) |
| Klebsiella infection | 3 | 10.06 (3.05–33.17) | 10.05 (22.01) | 9.15 (2.77) | 3.19 (0.05) |
| Systemic scleroderma | 3 | 20.89 (5.95–73.33) | 20.88 (46.14) | 17.15 (4.89) | 4.1 (0.15) |
| Ischaemic cardiomyopathy | 3 | 38.8 (10.03–150.08) | 38.78 (77.29) | 27.44 (7.09) | 4.78 (0.15) |
| Retinal artery occlusion | 3 | 20.89 (5.95–73.33) | 20.88 (46.14) | 17.15 (4.89) | 4.1 (0.15) |
| Cerebellar ataxia | 3 | 15.09 (4.44–51.24) | 15.08 (33.81) | 13.07 (3.85) | 3.71 (0.12) |
| Coronary artery occlusion | 3 | 11.81 (3.54–39.34) | 11.8 (26.24) | 10.56 (3.17) | 3.4 (0.08) |
| Psychomotor skills impaired | 3 | 24.69 (6.89–88.52) | 24.68 (53.55) | 19.6 (5.47) | 4.29 (0.16) |
| Cd4 lymphocytes decreased | 3 | 54.32 (12.98–227.35) | 54.29 (98.07) | 34.3 (8.2) | 5.1 (0.13) |
| Carotid artery thrombosis | 3 | 67.9 (15.19–303.44) | 67.86 (112.93) | 39.2 (8.77) | 5.29 (0.11) |
| Drug monitoring procedure not performed | 3 | 54.32 (12.98–227.35) | 54.29 (98.07) | 34.3 (8.2) | 5.1 (0.13) |
| Intra-abdominal haemorrhage | 3 | 24.69 (6.89–88.52) | 24.68 (53.55) | 19.6 (5.47) | 4.29 (0.16) |

Supplementary Table S7A

Age-stratified disproportionality analysis of adverse events associated with carboplatin at the Preferred Term level

| Age-stratified disproportionality analysis of adverse events associated with carboplatin at the Preferred Term level. | | | |
| --- | --- | --- | --- |
| PT | Age<65 (n) | Age≥65 (n) | ROR (95%CI) |
| Both age groups |  |  |  |
| Pancytopenia | 195 | 298 | 0.92 (0.77-1.10) |
| Leukopenia | 129 | 172 | 1.05 (0.84-1.33) |
| Mucosal inflammation | 62 | 81 | 1.08 (0.77-1.50) |
| Therapy partial responder | 60 | 57 | 1.48 (1.03-2.13) |
| Septic shock | 58 | 96 | 0.85 (0.61-1.18) |
| Neutropenic sepsis | 34 | 79 | 0.60 (0.40-0.90) |
| Polyneuropathy | 31 | 67 | 0.65 (0.42-0.99) |
| Therapy non-responder | 28 | 25 | 1.58 (0.92-2.70) |
| Posterior reversible encephalopathy syndrome | 28 | 17 | 2.32 (1.27-4.24) |
| Febrile bone marrow aplasia | 22 | 44 | 0.70 (0.42-1.17) |
| Immune-mediated hypothyroidism | 18 | 24 | 1.05 (0.57-1.94) |
| Congenital aplasia | 14 | 16 | 1.23 (0.60-2.52) |
| Bicytopenia | 13 | 19 | 0.96 (0.47-1.95) |
| Endocarditis | 12 | 8 | 2.11 (0.86-5.16) |
| Immune-mediated hyperthyroidism | 6 | 17 | 0.50 (0.20-1.26) |
| Age<65 specific |  |  |  |
| Hepatic cytolysis | 56 | - | - |
| Therapeutic product effect incomplete | 44 | - | - |
| Anaphylactic shock | 43 | - | - |
| Therapeutic response decreased | 20 | - | - |
| Restlessness | 16 | - | - |
| Angioedema | 15 | - | - |
| Myelodysplastic syndrome | 14 | - | - |
| Renal tubular disorder | 14 | - | - |
| Autoimmune haemolytic anaemia | 14 | - | - |
| Neutropenic colitis | 13 | - | - |
| Lip oedema | 11 | - | - |
| Jugular vein thrombosis | 11 | - | - |
| Rash vesicular | 10 | - | - |
| Raynaud's phenomenon | 10 | - | - |
| Faecaloma | 9 | - | - |
| Contraindicated product administered | 9 | - | - |
| Herpes simplex reactivation | 8 | - | - |
| Cutaneous lupus erythematosus | 8 | - | - |
| Exposure during pregnancy | 7 | - | - |
| Fanconi syndrome | 6 | - | - |
| Hyperphosphaturia | 6 | - | - |
| Antinuclear antibody | 6 | - | - |
| Aplasia pure red cell | 6 | - | - |
| Optic ischaemic neuropathy | 6 | - | - |
| Magnetic resonance imaging abnormal | 6 | - | - |
| Blood phosphorus abnormal | 6 | - | - |
| Stenosis | 5 | - | - |
| Paraneoplastic rash | 5 | - | - |
| Normocytic anaemia | 5 | - | - |
| Age≥65 specific |  |  |  |
| Thrombocytopenia | - | 304 | - |
| Neutropenia | - | 296 | - |
| Agranulocytosis | - | 56 | - |
| Hypomagnesaemia | - | 51 | - |
| Rash maculo-papular | - | 49 | - |
| Hypertransaminasaemia | - | 40 | - |
| Hepatocellular injury | - | 31 | - |
| Toxic skin eruption | - | 30 | - |
| Acute myeloid leukaemia | - | 22 | - |
| Lymphopenia | - | 21 | - |
| Encephalitis autoimmune | - | 21 | - |
| Clostridium difficile colitis | - | 21 | - |
| Prescribed overdose | - | 20 | - |
| Dermatitis bullous | - | 20 | - |
| Dermatitis exfoliative generalised | - | 18 | - |
| Extremity necrosis | - | 13 | - |
| Myelitis transverse | - | 12 | - |
| Meningitis aseptic | - | 12 | - |
| Peripheral sensorimotor neuropathy | - | 11 | - |
| Troponin t increased | - | 10 | - |
| Jaundice cholestatic | - | 10 | - |
| Muscle fatigue | - | 9 | - |
| Pancreatic failure | - | 8 | - |
| Systemic lupus erythematosus | - | 8 | - |
| Autoimmune hypothyroidism | - | 8 | - |
| Clonus | - | 7 | - |
| Oesophageal disorder | - | 7 | - |
| Oesophagitis ulcerative | - | 7 | - |
| Febrile infection | - | 7 | - |
| Intestinal pseudo-obstruction | - | 7 | - |
| Glucocorticoid deficiency | - | 7 | - |
| Large intestine infection | - | 7 | - |
| Shock symptom | - | 6 | - |
| Infusion site erythema | - | 6 | - |
| Blood creatine phosphokinase abnormal | - | 6 | - |
| Infusion related hypersensitivity reaction | - | 6 | - |
| Immune-mediated encephalopathy | - | 6 | - |

*For age-stratified analyses, RORs compare patients aged <65 years with those aged ≥65 years; ROR >1 indicates higher reporting odds in patients aged <65 years*.

Supplementary Table S7B

Sex-stratified disproportionality analysis of adverse events associated with carboplatin at the Preferred Term level

| Sex-stratified disproportionality analysis of adverse events associated with carboplatin at the Preferred Term level | | | |
| --- | --- | --- | --- |
| PT | Male | Female | ROR (95%CI) |
| Both sexes |  |  |  |
| Pancytopenia | 334 | 174 | 1.31 (1.09-1.57) |
| Neutropenia | 320 | 163 | 1.34 (1.11-1.62) |
| Thrombocytopenia | 313 | 150 | 1.42 (1.17-1.73) |
| Leukopenia | 214 | 98 | 1.49 (1.17-1.89) |
| Septic shock | 121 | 33 | 2.50 (1.70-3.67) |
| Mucosal inflammation | 94 | 57 | 1.12 (0.80-1.56) |
| Therapy partial responder | 73 | 46 | 1.08 (0.74-1.56) |
| Neutropenic sepsis | 72 | 49 | 1.00 (0.69-1.43) |
| Polyneuropathy | 70 | 38 | 1.25 (0.84-1.86) |
| Agranulocytosis | 60 | 22 | 1.85 (1.14-3.02) |
| Hepatic cytolysis | 52 | 33 | 1.07 (0.69-1.65) |
| Rash maculo-papular | 50 | 32 | 1.06 (0.68-1.65) |
| Febrile bone marrow aplasia | 41 | 25 | 1.11 (0.68-1.83) |
| Hypomagnesaemia | 39 | 39 | 0.68 (0.43-1.06) |
| Anaphylactic shock | 36 | 39 | 0.62 (0.40-0.98) |
| Hypertransaminasaemia | 35 | 23 | 1.03 (0.61-1.75) |
| Therapeutic product effect incomplete | 33 | 34 | 0.66 (0.41-1.06) |
| Acute myeloid leukaemia | 27 | 13 | 1.41 (0.73-2.73) |
| Immune-mediated hypothyroidism | 26 | 17 | 1.04 (0.56-1.91) |
| Bicytopenia | 24 | 7 | 2.33 (1.00-5.40) |
| Congenital aplasia | 20 | 10 | 1.36 (0.63-2.90) |
| Posterior reversible encephalopathy syndrome | 17 | 26 | 0.44 (0.24-0.82) |
| Dermatitis exfoliative generalised | 17 | 10 | 1.15 (0.53-2.52) |
| Immune-mediated hyperthyroidism | 14 | 9 | 1.05 (0.46-2.44) |
| Contraindicated product administered | 9 | 4 | 1.52 (0.47-4.95) |
| Male specific |  |  |  |
| Therapy non-responder | 41 | - | - |
| Hepatocellular injury | 37 | - | - |
| Toxic epidermal necrolysis | 35 | - | - |
| Therapeutic response decreased | 21 | - | - |
| Encephalitis autoimmune | 18 | - | - |
| Aphthous ulcer | 16 | - | - |
| Acute generalised exanthematous pustulosis | 16 | - | - |
| Faecaloma | 11 | - | - |
| Peripheral sensorimotor neuropathy | 11 | - | - |
| Demyelinating polyneuropathy | 11 | - | - |
| Lip oedema | 10 | - | - |
| Progressive multifocal leukoencephalopathy | 10 | - | - |
| Jugular vein thrombosis | 9 | - | - |
| Necrotising myositis | 9 | - | - |
| Magnetic resonance imaging abnormal | 9 | - | - |
| Sarcoidosis | 8 | - | - |
| Acute promyelocytic leukaemia | 8 | - | - |
| Intestinal pseudo-obstruction | 8 | - | - |
| Cutaneous lupus erythematosus | 8 | - | - |
| Herpes simplex reactivation | 8 | - | - |
| Clonus | 7 | - | - |
| Oesophagitis ulcerative | 7 | - | - |
| Large intestine infection | 7 | - | - |
| Vitritis | 6 | - | - |
| Cytomegalovirus colitis | 6 | - | - |
| Blood creatine phosphokinase abnormal | 6 | - | - |
| Anaemia of malignant disease | 6 | - | - |
| Acute interstitial pneumonitis | 6 | - | - |
| Idiopathic intracranial hypertension | 6 | - | - |
| Immune-mediated encephalopathy | 6 | - | - |
| Fournier's gangrene | 5 | - | - |
| Paraneoplastic rash | 5 | - | - |
| Female specific |  |  |  |
| Anaemia | - | 258 | - |
| Febrile neutropenia | - | 145 | - |
| Acute kidney injury | - | 129 | - |
| Hypokalaemia | - | 74 | - |
| Hypothyroidism | - | 68 | - |
| Colitis | - | 65 | - |
| Condition aggravated | - | 60 | - |
| Hepatitis | - | 48 | - |
| Hyperthyroidism | - | 41 | - |
| Immune-mediated enterocolitis | - | 39 | - |
| Toxic skin eruption | - | 29 | - |
| Hepatic failure | - | 25 | - |
| Renal tubular disorder | - | 22 | - |
| Immune-mediated hepatitis | - | 22 | - |
| Renal tubular necrosis | - | 21 | - |
| Dermatitis bullous | - | 20 | - |
| Purpura | - | 19 | - |
| Restlessness | - | 19 | - |
| Autoimmune hepatitis | - | 18 | - |
| Acute myocardial infarction | - | 18 | - |
| Disease recurrence | - | 15 | - |
| Nephritis | - | 14 | - |
| Prescribed overdose | - | 13 | - |
| Hypovolaemia | - | 12 | - |
| Pyelonephritis | - | 12 | - |
| Myelodysplastic syndrome | - | 12 | - |
| Raynaud's phenomenon | - | 12 | - |
| Neutropenic colitis | - | 11 | - |
| Endocarditis | - | 10 | - |
| Autoimmune haemolytic anaemia | - | 10 | - |
| Subacute cutaneous lupus erythematosus | - | 10 | - |
| Macular oedema | - | 9 | - |
| Hepatitis acute | - | 9 | - |
| Immune-mediated nephritis | - | 9 | - |
| Immune-mediated hypophysitis | - | 9 | - |
| Myelitis transverse | - | 8 | - |
| Rash morbilliform | - | 8 | - |
| Autoimmune colitis | - | 8 | - |
| Escherichia sepsis | - | 8 | - |
| Immune-mediated pancreatitis | - | 8 | - |
| Bronchopulmonary aspergillosis | - | 8 | - |
| Thrombocytosis | - | 7 | - |
| Oesophageal disorder | - | 7 | - |
| Autoimmune nephritis | - | 7 | - |
| Acute pulmonary oedema | - | 7 | - |
| Pancreatic failure | - | 7 | - |
| Sputum discoloured | - | 7 | - |
| Escherichia infection | - | 7 | - |
| Febrile infection | - | 7 | - |
| Exposure during pregnancy | - | 7 | - |
| Squamous cell carcinoma of lung | - | 7 | - |
| Rales | - | 6 | - |
| Shock symptom | - | 6 | - |
| Pneumonia pneumococcal | - | 6 | - |
| Troponin t increased | - | 6 | - |
| Hyperphosphaturia | - | 6 | - |
| Infusion site erythema | - | 6 | - |
| Antinuclear antibody | - | 6 | - |
| Meningitis aseptic | - | 6 | - |
| Blood corticotrophin decreased | - | 6 | - |
| Immune-mediated thyroiditis | - | 6 | - |
| Optic ischaemic neuropathy | - | 6 | - |
| Superficial vein thrombosis | - | 6 | - |
| Autoimmune hypothyroidism | - | 6 | - |
| Blood lactic acid increased | - | 6 | - |
| Blood phosphorus abnormal | - | 6 | - |
| Stenosis | - | 5 | - |
| Vascular purpura | - | 5 | - |
| Pemphigus | - | 5 | - |
| Terminal agitation | - | 5 | - |
| Punctate keratitis | - | 5 | - |
| Type i hypersensitivity | - | 5 | - |
| Blindness transient | - | 5 | - |
| Tumour hyperprogression | - | 5 | - |
| Cholestatic liver injury | - | 5 | - |
| Escherichia test positive | - | 5 | - |
| Tracheo-oesophageal fistula | - | 5 | - |
| Immune-mediated arthritis | - | 5 | - |
| Wound necrosis | - | 4 | - |
| Scleroedema | - | 4 | - |
| Fungaemia | - | 4 | - |
| Hyperplasia | - | 4 | - |
| Abdominal mass | - | 4 | - |
| Oligoastrocytoma | - | 4 | - |
| Vestibular disorder | - | 4 | - |
| Escherichia bacteraemia | - | 4 | - |
| Fusarium infection | - | 4 | - |
| Spinal cord infarction | - | 4 | - |
| Lung carcinoma cell type unspecified stage iii | - | 4 | - |
| Cardiovascular insufficiency | - | 4 | - |
| Nephrogenic diabetes insipidus | - | 4 | - |
| Vanishing bile duct syndrome | - | 4 | - |

*For sex-stratified analyses, RORs compare male patients with female patients; ROR >1 indicates higher reporting odds in male patients.*

Supplementary Table S8A

Age-stratified disproportionality analysis of adverse events associated with cisplatin at the Preferred Term level

| Age-stratified disproportionality analysis of adverse events associated with cisplatin at the Preferred Term level. | | | |
| --- | --- | --- | --- |
| PT | Age<65 (n) | Age≥65 (n) | ROR (95%CI) |
| Both age groups |  |  |  |
| Neutropenia | 69 | 55 | 0.81 (0.56-1.15) |
| Acute kidney injury | 48 | 37 | 0.84 (0.54-1.29) |
| Pancytopenia | 37 | 32 | 0.74 (0.46-1.20) |
| Septic shock | 17 | 18 | 0.61 (0.31-1.18) |
| Renal salt-wasting syndrome | 13 | 3 | 2.81 (0.80-9.87) |
| Nephropathy toxic | 12 | 8 | 0.97 (0.40-2.38) |
| Acute myocardial infarction | 12 | 10 | 0.77 (0.33-1.80) |
| Neutropenic sepsis | 11 | 20 | 0.35 (0.17-0.74) |
| Gastrointestinal toxicity | 11 | 5 | 1.42 (0.49-4.10) |
| Renal tubular necrosis | 9 | 10 | 0.58 (0.24-1.43) |
| Peripheral artery thrombosis | 5 | 4 | 0.81 (0.22-3.01) |
| Arterial thrombosis | 4 | 6 | 0.43 (0.12-1.53) |
| Cerebral venous thrombosis | 4 | 3 | 0.86 (0.19-3.85) |
| Age<65 specific |  |  |  |
| Aortic thrombosis | 28 | - | - |
| Oesophagitis | 16 | - | - |
| Posterior reversible encephalopathy syndrome | 13 | - | - |
| Tinnitus | 10 | - | - |
| Renal infarct | 10 | - | - |
| Hypomagnesaemia | 10 | - | - |
| Hyperkalaemia | 10 | - | - |
| Swelling face | 9 | - | - |
| Orthostatic hypotension | 9 | - | - |
| Capillary leak syndrome | 9 | - | - |
| Febrile bone marrow aplasia | 9 | - | - |
| Presyncope | 8 | - | - |
| Inappropriate antidiuretic hormone secretion | 8 | - | - |
| Hypoacusis | 7 | - | - |
| Peripheral ischaemia | 7 | - | - |
| Bicytopenia | 6 | - | - |
| Enteritis | 6 | - | - |
| Melanoderma | 6 | - | - |
| Ototoxicity | 6 | - | - |
| Polyarthritis | 5 | - | - |
| Hypovolaemia | 5 | - | - |
| Pulmonary nocardiosis | 5 | - | - |
| Dilated cardiomyopathy | 5 | - | - |
| Guillain-barre syndrome | 5 | - | - |
| Splenic infarction | 5 | - | - |
| Neutropenic colitis | 5 | - | - |
| Hypercreatininaemia | 5 | - | - |
| Hepatitis b | 4 | - | - |
| Tonsillitis | 4 | - | - |
| Skin hyperpigmentation | 4 | - | - |
| Type i hypersensitivity | 4 | - | - |
| Recall phenomenon | 4 | - | - |
| Small intestinal obstruction | 4 | - | - |
| Arterial occlusive disease | 4 | - | - |
| Salmonellosis | 3 | - | - |
| Pulse absent | 3 | - | - |
| Myopericarditis | 3 | - | - |
| Haemoperitoneum | 3 | - | - |
| Tearfulness | 3 | - | - |
| Splenic rupture | 3 | - | - |
| Ophthalmoplegia | 3 | - | - |
| Oesophageal fistula | 3 | - | - |
| Ventricular dysfunction | 3 | - | - |
| Mallory-weiss syndrome | 3 | - | - |
| Klebsiella infection | 3 | - | - |
| Cerebellar ataxia | 3 | - | - |
| Coronary artery thrombosis | 3 | - | - |
| Psychomotor skills impaired | 3 | - | - |
| Cd4 lymphocytes decreased | 3 | - | - |
| Age≥65 specific |  |  |  |
| Febrile neutropenia | - | 53 | - |
| Anaemia | - | 51 | - |
| Thrombocytopenia | - | 38 | - |
| Leukopenia | - | 28 | - |
| Renal failure | - | 24 | - |
| Hyponatraemia | - | 24 | - |
| Multiple organ dysfunction syndrome | - | 11 | - |
| Bone marrow failure | - | 10 | - |
| Embolism | - | 7 | - |
| Hypocalcaemia | - | 7 | - |
| Large intestine perforation | - | 7 | - |
| Cytomegalovirus oesophagitis | - | 7 | - |
| Coma | - | 5 | - |
| Peritonitis | - | 5 | - |
| Hypoparathyroidism | - | 5 | - |
| Metabolic acidosis | - | 5 | - |
| Gastroenteritis | - | 4 | - |
| Hemiparesis | - | 4 | - |
| Ileus paralytic | - | 4 | - |
| Intestinal ischaemia | - | 4 | - |
| Circulatory collapse | - | 4 | - |
| Empyema | - | 3 | - |
| Fibrosis | - | 3 | - |
| Kaposi's sarcoma | - | 3 | - |
| Ischaemic cardiomyopathy | - | 3 | - |
| Coronary artery occlusion | - | 3 | - |
| Carotid artery thrombosis | - | 3 | - |

*For age-stratified analyses, RORs compare patients aged <65 years with those aged ≥65 years; ROR >1 indicates higher reporting odds in patients aged <65 years*.

Supplementary Table S8B

Sex-stratified disproportionality analysis of adverse events associated with cisplatin at the Preferred Term level

| Sex-stratified disproportionality analysis of adverse events associated with cisplatin at the Preferred Term level | | | |
| --- | --- | --- | --- |
| PT | Male | Female | ROR (95%CI) |
| Both sexes |  |  |  |
| Neutropenia | 76 | 57 | 0.83 (0.59-1.18) |
| Acute kidney injury | 66 | 30 | 1.39 (0.90-2.14) |
| Febrile neutropenia | 63 | 30 | 1.32 (0.85-2.05) |
| Pancytopenia | 48 | 18 | 1.68 (0.97-2.90) |
| Leukopenia | 41 | 16 | 1.61 (0.90-2.88) |
| Neutropenic sepsis | 22 | 7 | 1.97 (0.84-4.63) |
| Aortic thrombosis | 16 | 13 | 0.77 (0.37-1.60) |
| Acute myocardial infarction | 13 | 9 | 0.90 (0.39-2.12) |
| Renal salt-wasting syndrome | 9 | 7 | 0.80 (0.30-2.16) |
| Tinnitus | 8 | 6 | 0.83 (0.29-2.41) |
| Renal infarct | 7 | 4 | 1.10 (0.32-3.75) |
| Renal tubular necrosis | 7 | 12 | 0.36 (0.14-0.93) |
| Cytomegalovirus oesophagitis | 6 | 3 | 1.25 (0.31-5.01) |
| Arterial occlusive disease | 5 | 4 | 0.78 (0.21-2.92) |
| Cerebral venous thrombosis | 4 | 3 | 0.83 (0.19-3.73) |
| Melanoderma | 3 | 5 | 0.38 (0.09-1.57) |
| Ototoxicity | 3 | 3 | 0.63 (0.13-3.10) |
| Peripheral artery thrombosis | 3 | 7 | 0.27 (0.07-1.04) |
| Male specific |  |  |  |
| Pulmonary embolism | 39 | - | - |
| Renal failure | 35 | - | - |
| Septic shock | 33 | - | - |
| Nephropathy toxic | 20 | - | - |
| Multiple organ dysfunction syndrome | 18 | - | - |
| Hypomagnesaemia | 12 | - | - |
| Orthostatic hypotension | 11 | - | - |
| Inappropriate antidiuretic hormone secretion | 10 | - | - |
| Enteritis | 9 | - | - |
| Hypocalcaemia | 9 | - | - |
| Arterial thrombosis | 9 | - | - |
| Bicytopenia | 8 | - | - |
| Peritonitis | 8 | - | - |
| Febrile bone marrow aplasia | 8 | - | - |
| Deafness | 7 | - | - |
| Splenic infarction | 7 | - | - |
| Metabolic acidosis | 7 | - | - |
| Hepatitis b | 6 | - | - |
| Ileus paralytic | 6 | - | - |
| Intestinal ischaemia | 5 | - | - |
| Hypoparathyroidism | 5 | - | - |
| Type i hypersensitivity | 5 | - | - |
| Neutropenic colitis | 5 | - | - |
| Idiopathic pulmonary fibrosis | 5 | - | - |
| Empyema | 4 | - | - |
| Photophobia | 4 | - | - |
| Hypovolaemia | 4 | - | - |
| Colitis ischaemic | 4 | - | - |
| Diastolic dysfunction | 4 | - | - |
| Coronary artery thrombosis | 4 | - | - |
| Diabetes mellitus inadequate control | 4 | - | - |
| Fibrosis | 3 | - | - |
| Ophthalmoplegia | 3 | - | - |
| Tonsillitis | 3 | - | - |
| Kaposi's sarcoma | 3 | - | - |
| Mallory-weiss syndrome | 3 | - | - |
| Klebsiella infection | 3 | - | - |
| Cerebellar ataxia | 3 | - | - |
| Progressive multifocal leukoencephalopathy | 3 | - | - |
| Psychomotor skills impaired | 3 | - | - |
| Cd4 lymphocytes decreased | 3 | - | - |
| Female specific |  |  |  |
| Sepsis | - | 19 | - |
| Gastrointestinal toxicity | - | 13 | - |
| Lung adenocarcinoma | - | 11 | - |
| Oesophagitis | - | 9 | - |
| Swelling face | - | 9 | - |
| Bone marrow failure | - | 9 | - |
| Capillary leak syndrome | - | 9 | - |
| Posterior reversible encephalopathy syndrome | - | 9 | - |
| Embolism | - | 8 | - |
| Ischaemic stroke | - | 7 | - |
| Haematotoxicity | - | 7 | - |
| Hemiparesis | - | 6 | - |
| Polyarthritis | - | 5 | - |
| Polyneuropathy | - | 5 | - |
| Agitation | - | 5 | - |
| Neurotoxicity | - | 5 | - |
| Depressed mood | - | 5 | - |
| Peripheral ischaemia | - | 5 | - |
| Lower respiratory tract infection | - | 5 | - |
| Cyanosis | - | 4 | - |
| Ataxia | - | 4 | - |
| Presyncope | - | 4 | - |
| Metastases to adrenals | - | 4 | - |
| Recall phenomenon | - | 4 | - |
| Small intestinal obstruction | - | 4 | - |
| Salmonellosis | - | 3 | - |
| Myopericarditis | - | 3 | - |
| Formication | - | 3 | - |
| Haemoperitoneum | - | 3 | - |
| Tearfulness | - | 3 | - |
| Splenic rupture | - | 3 | - |
| Pulmonary nocardiosis | - | 3 | - |
| Dilated cardiomyopathy | - | 3 | - |
| Radiation oesophagitis | - | 3 | - |
| Skin hyperpigmentation | - | 3 | - |
| Systemic scleroderma | - | 3 | - |
| Oesophageal ulcer | - | 3 | - |
| Henoch-schonlein purpura | - | 3 | - |
| Coronary artery occlusion | - | 3 | - |
| Contraindicated product administered | - | 3 | - |
| Carotid artery thrombosis | - | 3 | - |
| Drug monitoring procedure not performed | - | 3 | - |
| Neutrophil count increased | - | 3 | - |
| Renal function test abnormal | - | 3 | - |

*For sex-stratified analyses, RORs compare male patients with female patients; ROR >1 indicates higher reporting odds in male patients.*

Supplementary Table S9A

Disproportionality analysis of carboplatin-associated positive signals after excluding pemetrexed co-reported cases in the FAERS database

| All qualifying carboplatin-associated preferred terms after excluding pemetrexed co-reported cases (n=19,285) | | | | | |
| --- | --- | --- | --- | --- | --- |
| PT | N | ROR (95%CI) | PRR(χ^2^) | EBGM(EBGM05) | IC(IC025) |
| Anaemia | 409 | 2.42 (2.19–2.68) | 2.39 (304.16) | 2.27 (2.05) | 1.18 (1.03) |
| Neutropenia | 354 | 2.96 (2.65–3.31) | 2.93 (402.84) | 2.72 (2.43) | 1.44 (1.27) |
| Thrombocytopenia | 318 | 2.92 (2.6–3.29) | 2.89 (353.7) | 2.69 (2.39) | 1.43 (1.25) |
| Febrile neutropenia | 310 | 2.96 (2.63–3.33) | 2.93 (352.65) | 2.72 (2.41) | 1.44 (1.26) |
| Leukopenia | 274 | 5.76 (5.05–6.58) | 5.7 (860.87) | 4.8 (4.2) | 2.26 (2.05) |
| Pancytopenia | 247 | 3.93 (3.43–4.5) | 3.89 (458.32) | 3.49 (3.05) | 1.8 (1.59) |
| Neutropenic sepsis | 118 | 8.63 (6.99–10.65) | 8.59 (583.96) | 6.6 (5.34) | 2.72 (2.35) |
| Hypokalaemia | 109 | 2.9 (2.37–3.54) | 2.89 (120.33) | 2.69 (2.2) | 1.43 (1.11) |
| Polyneuropathy | 103 | 12.95 (10.19–16.46) | 12.89 (737.07) | 8.75 (6.89) | 3.13 (2.69) |
| Septic shock | 100 | 3.22 (2.62–3.97) | 3.21 (134.69) | 2.95 (2.4) | 1.56 (1.23) |
| Therapy partial responder | 68 | 2.98 (2.32–3.84) | 2.98 (79.51) | 2.76 (2.14) | 1.46 (1.06) |
| Hypomagnesaemia | 66 | 4.59 (3.53–5.98) | 4.58 (155.43) | 4.01 (3.08) | 2 (1.56) |
| Agranulocytosis | 59 | 4.94 (3.73–6.54) | 4.93 (153.67) | 4.26 (3.22) | 2.09 (1.61) |
| Disease recurrence | 53 | 9.58 (6.97–13.17) | 9.55 (290.97) | 7.13 (5.19) | 2.83 (2.23) |
| Anaphylactic shock | 50 | 4.19 (3.1–5.66) | 4.18 (103.15) | 3.71 (2.75) | 1.89 (1.38) |
| Immune-mediated enterocolitis | 49 | 3.33 (2.47–4.49) | 3.32 (70.07) | 3.04 (2.26) | 1.61 (1.11) |
| Therapy non-responder | 46 | 4.66 (3.4–6.39) | 4.65 (110.55) | 4.06 (2.96) | 2.02 (1.47) |
| Immune-mediated hypothyroidism | 37 | 7.65 (5.29–11.08) | 7.64 (162.24) | 6.04 (4.17) | 2.6 (1.88) |
| Lymphopenia | 36 | 5.09 (3.55–7.3) | 5.09 (97.64) | 4.37 (3.05) | 2.13 (1.48) |
| Lower respiratory tract infection | 35 | 3.44 (2.41–4.9) | 3.44 (52.97) | 3.13 (2.2) | 1.65 (1.05) |
| Posterior reversible encephalopathy syndrome | 35 | 5.07 (3.52–7.3) | 5.06 (94.37) | 4.36 (3.03) | 2.12 (1.47) |
| Myelodysplastic syndrome | 34 | 7.83 (5.32–11.54) | 7.82 (152.84) | 6.15 (4.18) | 2.62 (1.87) |
| Inappropriate antidiuretic hormone secretion | 34 | 3.72 (2.59–5.34) | 3.72 (58.53) | 3.35 (2.34) | 1.75 (1.13) |
| Acute myeloid leukaemia | 33 | 5.5 (3.77–8.04) | 5.5 (98.94) | 4.66 (3.19) | 2.22 (1.53) |
| Tumour lysis syndrome | 29 | 5.94 (3.96–8.92) | 5.94 (95.58) | 4.96 (3.3) | 2.31 (1.55) |
| Bronchospasm | 26 | 3.31 (2.2–4.99) | 3.31 (36.79) | 3.03 (2.01) | 1.6 (0.9) |
| Oral candidiasis | 23 | 3.41 (2.2–5.28) | 3.41 (34.31) | 3.11 (2.01) | 1.64 (0.88) |
| Immune-mediated hyperthyroidism | 23 | 25.28 (14.09–45.37) | 25.25 (261.93) | 12.86 (7.16) | 3.68 (2.36) |
| Bicytopenia | 22 | 7.19 (4.46–11.57) | 7.18 (90.24) | 5.76 (3.58) | 2.53 (1.57) |
| Prescribed overdose | 22 | 4.67 (2.96–7.37) | 4.66 (53.05) | 4.07 (2.58) | 2.02 (1.18) |
| Restlessness | 21 | 4.57 (2.87–7.29) | 4.57 (49.26) | 4 (2.51) | 2 (1.14) |
| Squamous cell carcinoma of lung | 19 | 6.56 (3.95–10.9) | 6.56 (70.38) | 5.37 (3.23) | 2.42 (1.41) |
| Pulmonary sepsis | 18 | 8.06 (4.73–13.74) | 8.05 (83.38) | 6.29 (3.69) | 2.65 (1.54) |
| Contraindicated product administered | 18 | 22.9 (12.02–43.65) | 22.88 (193.45) | 12.24 (6.42) | 3.61 (2.11) |
| Encephalitis autoimmune | 17 | 3.99 (2.39–6.67) | 3.99 (32.66) | 3.56 (2.13) | 1.83 (0.9) |
| Urosepsis | 15 | 5.25 (3.01–9.19) | 5.25 (42.41) | 4.49 (2.57) | 2.17 (1.08) |
| Endocarditis | 14 | 7.05 (3.89–12.79) | 7.05 (56.23) | 5.68 (3.13) | 2.51 (1.27) |
| Pyelonephritis | 14 | 4.77 (2.69–8.46) | 4.76 (34.77) | 4.14 (2.34) | 2.05 (0.96) |
| Radiation oesophagitis | 14 | 4.77 (2.69–8.46) | 4.76 (34.77) | 4.14 (2.34) | 2.05 (0.96) |
| Thrombocytosis | 13 | 5.33 (2.92–9.71) | 5.32 (37.4) | 4.54 (2.49) | 2.18 (1) |
| Myelitis transverse | 13 | 12.57 (6.43–24.57) | 12.56 (91.01) | 8.61 (4.4) | 3.11 (1.56) |
| Non-cardiac chest pain | 13 | 4.91 (2.7–8.91) | 4.91 (33.61) | 4.25 (2.34) | 2.09 (0.93) |
| Immune-mediated hypophysitis | 13 | 9.24 (4.88–17.51) | 9.24 (69.07) | 6.96 (3.67) | 2.8 (1.39) |
| Papilloedema | 12 | 8.79 (4.54–17.02) | 8.78 (60.7) | 6.71 (3.46) | 2.75 (1.29) |
| Cutaneous vasculitis | 12 | 5.09 (2.73–9.48) | 5.09 (32.54) | 4.37 (2.35) | 2.13 (0.91) |
| Jugular vein thrombosis | 11 | 6.65 (3.41–12.95) | 6.64 (41.36) | 5.43 (2.78) | 2.44 (1.04) |
| Progressive multifocal leukoencephalopathy | 11 | 8.31 (4.19–16.48) | 8.3 (52.59) | 6.43 (3.24) | 2.69 (1.19) |
| Kounis syndrome | 10 | 60.42 (18.95–192.66) | 60.39 (166.87) | 17.97 (5.63) | 4.17 (1.64) |
| Escherichia infection | 9 | 4.94 (2.41–10.13) | 4.94 (23.49) | 4.27 (2.09) | 2.09 (0.67) |
| Sputum discoloured | 9 | 4.83 (2.36–9.89) | 4.83 (22.79) | 4.19 (2.05) | 2.07 (0.66) |
| Febrile infection | 9 | 5.3 (2.58–10.91) | 5.3 (25.76) | 4.53 (2.2) | 2.18 (0.73) |
| Anaemia of malignant disease | 9 | 18.12 (7.64–43.02) | 18.12 (83.17) | 10.78 (4.54) | 3.43 (1.31) |
| Vascular device infection | 9 | 14.5 (6.34–33.14) | 14.49 (70.66) | 9.43 (4.13) | 3.24 (1.25) |
| Pancreatic failure | 8 | 9.21 (4.08–20.78) | 9.2 (42.35) | 6.94 (3.07) | 2.79 (0.94) |
| Acute promyelocytic leukaemia | 8 | 8.05 (3.62–17.93) | 8.05 (37.06) | 6.29 (2.82) | 2.65 (0.88) |
| Autoimmune hypothyroidism | 8 | 8.79 (3.91–19.74) | 8.78 (40.47) | 6.71 (2.99) | 2.75 (0.92) |
| Rales | 7 | 6.51 (2.82–14.99) | 6.5 (25.69) | 5.34 (2.32) | 2.42 (0.63) |
| Endophthalmitis | 7 | 42.29 (12.38–144.46) | 42.27 (102.57) | 16.01 (4.69) | 4 (1.12) |
| Cytotoxic oedema | 7 | 169.14 (20.81–1374.88) | 169.08 (146.2) | 22.01 (2.71) | 4.46 (1.16) |
| Oesophageal disorder | 7 | 5.83 (2.55–13.32) | 5.83 (22.57) | 4.89 (2.14) | 2.29 (0.57) |
| Terminal agitation | 7 | 84.57 (17.57–407.14) | 84.54 (128.42) | 19.56 (4.06) | 4.29 (1.15) |
| Type i hypersensitivity | 7 | 6.77 (2.93–15.64) | 6.76 (26.86) | 5.5 (2.38) | 2.46 (0.65) |
| Blood lactic acid increased | 7 | 8.46 (3.58–20) | 8.45 (34.08) | 6.52 (2.76) | 2.71 (0.76) |
| Chronic myeloid leukaemia | 7 | 13.01 (5.19–32.61) | 13.01 (50.43) | 8.8 (3.51) | 3.14 (0.93) |
| Vitritis | 6 | 48.32 (12.08–193.24) | 48.31 (92.66) | 16.77 (4.19) | 4.07 (0.9) |
| Shock symptom | 6 | 16.11 (5.73–45.26) | 16.1 (51) | 10.06 (3.58) | 3.33 (0.79) |
| Pseudomonal sepsis | 6 | 14.5 (5.27–39.89) | 14.49 (47.11) | 9.43 (3.43) | 3.24 (0.77) |
| Paraneoplastic rash | 6 | 48.32 (12.08–193.24) | 48.31 (92.66) | 16.77 (4.19) | 4.07 (0.9) |
| Antinuclear antibody | 6 | 48.32 (12.08–193.24) | 48.31 (92.66) | 16.77 (4.19) | 4.07 (0.9) |
| Laryngeal cancer stage iii | 6 | 144.97 (17.45–1204.28) | 144.93 (122.52) | 21.56 (2.6) | 4.43 (0.92) |
| Pulmonary radiation injury | 6 | 10.35 (3.98–26.95) | 10.35 (35.49) | 7.55 (2.9) | 2.92 (0.67) |
| Infective exacerbation of chronic obstructive airways disease | 6 | 9.66 (3.75–24.91) | 9.66 (33.28) | 7.19 (2.79) | 2.85 (0.65) |
| Blood electrolytes abnormal | 6 | 8.05 (3.2–20.29) | 8.05 (27.79) | 6.29 (2.5) | 2.65 (0.58) |
| Vasogenic cerebral oedema | 6 | 6.04 (2.47–14.78) | 6.04 (20.18) | 5.03 (2.06) | 2.33 (0.44) |
| Lung carcinoma cell type unspecified stage iii | 6 | 48.32 (12.08–193.24) | 48.31 (92.66) | 16.77 (4.19) | 4.07 (0.9) |
| Idiopathic intracranial hypertension | 6 | 24.16 (7.79–74.92) | 24.15 (66.59) | 12.58 (4.06) | 3.65 (0.85) |
| Immune-mediated encephalopathy | 6 | 11.15 (4.24–29.34) | 11.15 (37.93) | 7.94 (3.02) | 2.99 (0.7) |
| Stenosis | 5 | 13.42 (4.5–40.06) | 13.42 (36.95) | 8.98 (3.01) | 3.17 (0.52) |
| Skin plaque | 5 | 8.05 (2.93–22.16) | 8.05 (23.16) | 6.29 (2.29) | 2.65 (0.37) |
| Intensive care | 5 | 15.1 (4.94–46.16) | 15.1 (40.5) | 9.67 (3.16) | 3.27 (0.54) |
| Amoebic colitis | 5 | 120.8 (14.11–1034.1) | 120.77 (98.99) | 20.96 (2.45) | 4.39 (0.63) |
| Megacolon | 5 | 8.63 (3.11–23.96) | 8.63 (24.84) | 6.62 (2.38) | 2.73 (0.4) |
| Abdominal mass | 5 | 13.42 (4.5–40.06) | 13.42 (36.95) | 8.98 (3.01) | 3.17 (0.52) |
| Vestibular disorder | 5 | 12.08 (4.13–35.35) | 12.08 (33.87) | 8.38 (2.87) | 3.07 (0.49) |
| Fournier's gangrene | 5 | 30.2 (8.11–112.48) | 30.19 (62.73) | 13.97 (3.75) | 3.8 (0.61) |
| Actinic keratosis | 5 | 10.07 (3.55–28.58) | 10.06 (28.81) | 7.4 (2.61) | 2.89 (0.45) |
| Infusion site erythema | 5 | 6.71 (2.49–18.08) | 6.71 (19.01) | 5.47 (2.03) | 2.45 (0.3) |
| Epidermolysis bullosa | 5 | 15.1 (4.94–46.16) | 15.1 (40.5) | 9.67 (3.16) | 3.27 (0.54) |
| Epiglottic oedema | 5 | 60.4 (11.72–311.36) | 60.39 (83.43) | 17.97 (3.49) | 4.17 (0.63) |
| Escherichia test positive | 5 | 15.1 (4.94–46.16) | 15.1 (40.5) | 9.67 (3.16) | 3.27 (0.54) |
| Subclavian artery occlusion | 5 | 40.27 (9.62–168.51) | 40.26 (71.78) | 15.72 (3.76) | 3.97 (0.62) |
| Exposure during pregnancy | 5 | 8.63 (3.11–23.96) | 8.63 (24.84) | 6.62 (2.38) | 2.73 (0.4) |
| Chronic myelomonocytic leukaemia | 5 | 17.26 (5.48–54.38) | 17.25 (44.66) | 10.48 (3.33) | 3.39 (0.56) |
| Oligoastrocytoma | 4 | 32.21 (7.21–143.94) | 32.21 (51.84) | 14.37 (3.22) | 3.85 (0.28) |
| Wound necrosis | 4 | 24.16 (6.04–96.61) | 24.15 (44.39) | 12.58 (3.15) | 3.65 (0.27) |
| Microangiopathy | 4 | 10.74 (3.31–34.87) | 10.74 (24.45) | 7.74 (2.38) | 2.95 (0.18) |
| Trigger finger | 4 | 19.33 (5.19–71.98) | 19.32 (38.61) | 11.18 (3) | 3.48 (0.26) |
| Pneumobilia | 4 | 24.16 (6.04–96.61) | 24.15 (44.39) | 12.58 (3.15) | 3.65 (0.27) |
| Mucormycosis | 4 | 16.11 (4.54–57.08) | 16.1 (34) | 10.06 (2.84) | 3.33 (0.24) |
| Oesophagopleural fistula | 4 | 96.64 (10.8–864.69) | 96.62 (75.7) | 20.12 (2.25) | 4.33 (0.27) |
| Gastritis bacterial | 4 | 48.32 (8.85–263.83) | 48.31 (61.77) | 16.77 (3.07) | 4.07 (0.28) |
| Enterovesical fistula | 4 | 13.81 (4.04–47.16) | 13.8 (30.23) | 9.15 (2.68) | 3.19 (0.22) |
| Vasculitis necrotising | 4 | 13.81 (4.04–47.16) | 13.8 (30.23) | 9.15 (2.68) | 3.19 (0.22) |
| Spinal cord infarction | 4 | 12.08 (3.64–40.12) | 12.08 (27.09) | 8.38 (2.52) | 3.07 (0.2) |
| Vascular access site swelling | 4 | 96.64 (10.8–864.69) | 96.62 (75.7) | 20.12 (2.25) | 4.33 (0.27) |
| Type iii immune complex mediated reaction | 4 | 48.32 (8.85–263.83) | 48.31 (61.77) | 16.77 (3.07) | 4.07 (0.28) |
| Complex regional pain syndrome | 4 | 16.11 (4.54–57.08) | 16.1 (34) | 10.06 (2.84) | 3.33 (0.24) |
| Intermittent claudication | 4 | 12.08 (3.64–40.12) | 12.08 (27.09) | 8.38 (2.52) | 3.07 (0.2) |
| Body temperature abnormal | 4 | 8.79 (2.8–27.59) | 8.78 (20.23) | 6.71 (2.14) | 2.75 (0.13) |
| Infusion related hypersensitivity reaction | 4 | 8.79 (2.8–27.59) | 8.78 (20.23) | 6.71 (2.14) | 2.75 (0.13) |
| Gastroenteritis norovirus | 4 | 19.33 (5.19–71.98) | 19.32 (38.61) | 11.18 (3) | 3.48 (0.26) |

Supplementary **Table S9B**

**Disproportionality analysis of** carbo**platin-associated positive signals after excluding paclitaxel co-reported cases in the FAERS database**

| All qualifying carboplatin-associated preferred terms after excluding paclitaxel co-reported cases (n=20,266) | | | | | |
| --- | --- | --- | --- | --- | --- |
| PT | N | ROR (95%CI) | PRR(χ^2^) | EBGM(EBGM05) | IC(IC025) |
| Pancytopenia | 452 | 7.96 (7.14–8.86) | 7.8 (2007.2) | 6.07 (5.45) | 2.6 (2.43) |
| Neutropenia | 404 | 3.27 (2.94–3.63) | 3.23 (547.54) | 2.95 (2.66) | 1.56 (1.4) |
| Thrombocytopenia | 392 | 3.53 (3.17–3.93) | 3.48 (605.92) | 3.16 (2.83) | 1.66 (1.49) |
| Febrile neutropenia | 356 | 3.29 (2.94–3.68) | 3.25 (488.43) | 2.97 (2.66) | 1.57 (1.4) |
| Acute kidney injury | 296 | 3.17 (2.8–3.58) | 3.13 (380.45) | 2.88 (2.55) | 1.53 (1.34) |
| Leukopenia | 182 | 3.35 (2.87–3.92) | 3.33 (259.73) | 3.03 (2.6) | 1.6 (1.36) |
| Neutropenic sepsis | 131 | 9.47 (7.73–11.62) | 9.42 (699.49) | 6.97 (5.68) | 2.8 (2.45) |
| Septic shock | 124 | 3.92 (3.24–4.75) | 3.91 (229.56) | 3.48 (2.88) | 1.8 (1.49) |
| Condition aggravated | 107 | 2.68 (2.19–3.27) | 2.67 (100.37) | 2.5 (2.04) | 1.32 (1.01) |
| Therapy partial responder | 107 | 4.8 (3.9–5.92) | 4.78 (265.33) | 4.13 (3.35) | 2.05 (1.7) |
| Mucosal inflammation | 100 | 2.7 (2.19–3.32) | 2.69 (95.14) | 2.51 (2.04) | 1.33 (1) |
| Bone marrow failure | 74 | 2.89 (2.27–3.69) | 2.89 (81.16) | 2.68 (2.1) | 1.42 (1.03) |
| Agranulocytosis | 71 | 5.9 (4.54–7.66) | 5.88 (229) | 4.88 (3.76) | 2.29 (1.83) |
| Disease recurrence | 65 | 12.26 (9.07–16.57) | 12.22 (437.05) | 8.32 (6.16) | 3.06 (2.48) |
| Hepatic cytolysis | 62 | 3.58 (2.74–4.68) | 3.57 (99.49) | 3.23 (2.47) | 1.69 (1.25) |
| Hypertransaminasaemia | 62 | 7.83 (5.87–10.46) | 7.81 (274.92) | 6.08 (4.56) | 2.6 (2.08) |
| Febrile bone marrow aplasia | 57 | 8.14 (6.02–11.02) | 8.12 (262.95) | 6.26 (4.63) | 2.65 (2.09) |
| Anaphylactic shock | 54 | 4.35 (3.25–5.83) | 4.35 (117.03) | 3.81 (2.85) | 1.93 (1.44) |
| Therapeutic product effect incomplete | 53 | 5.56 (4.12–7.51) | 5.55 (159.31) | 4.66 (3.45) | 2.22 (1.69) |
| Tubulointerstitial nephritis | 53 | 2.9 (2.18–3.86) | 2.89 (58.41) | 2.68 (2.01) | 1.42 (0.96) |
| Hepatocellular injury | 49 | 4.38 (3.23–5.95) | 4.37 (107.14) | 3.83 (2.82) | 1.94 (1.41) |
| Hypomagnesaemia | 47 | 2.94 (2.17–3.99) | 2.94 (53.29) | 2.72 (2.01) | 1.44 (0.95) |
| Therapy non-responder | 36 | 3.32 (2.34–4.71) | 3.32 (50.91) | 3.02 (2.13) | 1.6 (1.01) |
| Renal tubular necrosis | 32 | 4.35 (2.98–6.35) | 4.34 (69.27) | 3.81 (2.61) | 1.93 (1.26) |
| Acute myeloid leukaemia | 32 | 5.03 (3.43–7.38) | 5.03 (84.71) | 4.3 (2.93) | 2.11 (1.41) |
| Inappropriate antidiuretic hormone secretion | 32 | 3.3 (2.27–4.77) | 3.29 (44.66) | 3 (2.07) | 1.59 (0.96) |
| Bicytopenia | 31 | 10.95 (7.14–16.81) | 10.94 (189.59) | 7.73 (5.04) | 2.95 (2.07) |
| Lymphopenia | 27 | 3.44 (2.3–5.16) | 3.44 (40.66) | 3.12 (2.08) | 1.64 (0.95) |
| Angioedema | 27 | 6.46 (4.21–9.9) | 6.45 (97.09) | 5.25 (3.43) | 2.39 (1.58) |
| Toxic skin eruption | 27 | 3.56 (2.37–5.34) | 3.56 (43.03) | 3.22 (2.14) | 1.69 (0.99) |
| Dermatitis exfoliative generalised | 27 | 11.07 (6.99–17.53) | 11.06 (166.7) | 7.79 (4.92) | 2.96 (2) |
| Autoimmune hepatitis | 26 | 3.47 (2.3–5.24) | 3.47 (39.67) | 3.14 (2.08) | 1.65 (0.94) |
| Renal tubular disorder | 26 | 9.19 (5.83–14.48) | 9.17 (135.3) | 6.84 (4.34) | 2.77 (1.85) |
| Immune-mediated hypothyroidism | 25 | 4.45 (2.9–6.83) | 4.45 (55.93) | 3.89 (2.53) | 1.96 (1.19) |
| Acute coronary syndrome | 24 | 5.1 (3.28–7.94) | 5.1 (64.69) | 4.35 (2.8) | 2.12 (1.3) |
| Tumour lysis syndrome | 23 | 4.26 (2.73–6.65) | 4.25 (48.31) | 3.75 (2.4) | 1.91 (1.11) |
| Congenital aplasia | 23 | 10.78 (6.57–17.69) | 10.77 (138.69) | 7.65 (4.66) | 2.93 (1.89) |
| Circulatory collapse | 22 | 3.83 (2.44–6.01) | 3.82 (39.32) | 3.42 (2.18) | 1.77 (0.98) |
| Prescribed overdose | 21 | 4.19 (2.63–6.68) | 4.19 (43.11) | 3.7 (2.32) | 1.89 (1.05) |
| Neutropenic colitis | 21 | 7.65 (4.67–12.54) | 7.65 (90.99) | 5.98 (3.65) | 2.58 (1.58) |
| Dermatitis bullous | 19 | 3.96 (2.44–6.45) | 3.96 (35.88) | 3.53 (2.17) | 1.82 (0.94) |
| Aphthous ulcer | 17 | 4.65 (2.76–7.82) | 4.64 (40.41) | 4.03 (2.39) | 2.01 (1.04) |
| Rash vesicular | 16 | 5.17 (3.01–8.9) | 5.17 (43.91) | 4.4 (2.56) | 2.14 (1.1) |
| Lip oedema | 16 | 18.36 (9.51–35.44) | 18.35 (145.82) | 10.64 (5.51) | 3.41 (1.89) |
| Extremity necrosis | 16 | 6.68 (3.83–11.65) | 6.67 (59.78) | 5.39 (3.09) | 2.43 (1.31) |
| Endocarditis | 15 | 7.32 (4.1–13.1) | 7.32 (62.06) | 5.79 (3.24) | 2.53 (1.33) |
| Immune-mediated nephritis | 15 | 5.74 (3.26–10.11) | 5.73 (46.91) | 4.79 (2.72) | 2.26 (1.14) |
| Hepatitis acute | 14 | 4.17 (2.36–7.38) | 4.17 (28.56) | 3.68 (2.08) | 1.88 (0.83) |
| Peripheral vein thrombosis | 14 | 4.23 (2.39–7.48) | 4.23 (29.11) | 3.72 (2.11) | 1.9 (0.84) |
| Subacute cutaneous lupus erythematosus | 14 | 4.66 (2.62–8.27) | 4.65 (33.39) | 4.04 (2.27) | 2.01 (0.92) |
| Pyelonephritis | 13 | 4.14 (2.29–7.48) | 4.14 (26.24) | 3.66 (2.03) | 1.87 (0.78) |
| Meningitis aseptic | 12 | 5.74 (3.05–10.8) | 5.73 (37.53) | 4.79 (2.54) | 2.26 (0.99) |
| Cutaneous vasculitis | 12 | 4.83 (2.59–9) | 4.83 (30.1) | 4.16 (2.23) | 2.06 (0.86) |
| Contraindicated product administered | 12 | 11.02 (5.53–21.93) | 11.01 (73.79) | 7.76 (3.9) | 2.96 (1.4) |
| Acute generalised exanthematous pustulosis | 12 | 4.44 (2.39–8.24) | 4.44 (26.79) | 3.88 (2.09) | 1.96 (0.79) |
| Progressive multifocal leukoencephalopathy | 12 | 8.88 (4.56–17.3) | 8.88 (60.49) | 6.68 (3.43) | 2.74 (1.28) |
| Papilloedema | 11 | 7.42 (3.76–14.65) | 7.42 (46.18) | 5.85 (2.96) | 2.55 (1.1) |
| Raynaud's phenomenon | 11 | 7.42 (3.76–14.65) | 7.42 (46.18) | 5.85 (2.96) | 2.55 (1.1) |
| Peripheral sensorimotor neuropathy | 11 | 10.98 (5.35–22.52) | 10.97 (67.43) | 7.74 (3.77) | 2.95 (1.32) |
| Immune-mediated hyperthyroidism | 11 | 7.42 (3.76–14.65) | 7.42 (46.18) | 5.85 (2.96) | 2.55 (1.1) |
| Hypercreatininaemia | 10 | 5.74 (2.87–11.47) | 5.73 (31.27) | 4.79 (2.39) | 2.26 (0.85) |
| Autoimmune hypothyroidism | 10 | 11.47 (5.37–24.52) | 11.47 (63.71) | 7.98 (3.73) | 3 (1.25) |
| Large intestine infection | 10 | 13.5 (6.18–29.48) | 13.49 (72.83) | 8.87 (4.06) | 3.15 (1.31) |
| Muscle fatigue | 9 | 6.66 (3.17–13.99) | 6.66 (33.55) | 5.39 (2.56) | 2.43 (0.86) |
| Palmar erythema | 9 | 9.39 (4.32–20.39) | 9.38 (47.84) | 6.95 (3.2) | 2.8 (1.05) |
| Dysentery | 9 | 5.9 (2.84–12.28) | 5.9 (29.12) | 4.9 (2.35) | 2.29 (0.79) |
| Macular oedema | 9 | 6.66 (3.17–13.99) | 6.66 (33.55) | 5.39 (2.56) | 2.43 (0.86) |
| Blood culture positive | 9 | 8.26 (3.86–17.7) | 8.26 (42.21) | 6.34 (2.96) | 2.66 (0.99) |
| Myelitis transverse | 9 | 7.12 (3.37–15.05) | 7.12 (36.12) | 5.67 (2.68) | 2.5 (0.9) |
| Aplasia pure red cell | 9 | 5.9 (2.84–12.28) | 5.9 (29.12) | 4.9 (2.35) | 2.29 (0.79) |
| Tumour hyperprogression | 9 | 4.92 (2.39–10.1) | 4.92 (23.12) | 4.22 (2.06) | 2.08 (0.66) |
| Necrotising myositis | 9 | 15.89 (6.79–37.17) | 15.88 (74.15) | 9.79 (4.19) | 3.29 (1.26) |
| Cutaneous lupus erythematosus | 9 | 7.65 (3.6–16.27) | 7.65 (38.99) | 5.98 (2.81) | 2.58 (0.95) |
| Optic ischaemic neuropathy | 9 | 11.47 (5.15–25.54) | 11.47 (57.34) | 7.98 (3.58) | 3 (1.14) |
| Magnetic resonance imaging abnormal | 9 | 20.65 (8.39–50.83) | 20.64 (88.54) | 11.34 (4.61) | 3.5 (1.33) |
| Immune-mediated hypophysitis | 9 | 5.43 (2.63–11.24) | 5.43 (26.32) | 4.58 (2.22) | 2.2 (0.73) |
| Tracheo-oesophageal fistula | 9 | 6.26 (2.99–13.08) | 6.26 (31.23) | 5.13 (2.45) | 2.36 (0.83) |
| Kounis syndrome | 8 | 30.59 (10.61–88.18) | 30.58 (98.11) | 13.68 (4.75) | 3.77 (1.25) |
| Rash morbilliform | 8 | 5.74 (2.64–12.45) | 5.73 (25.02) | 4.79 (2.21) | 2.26 (0.67) |
| Intestinal pseudo-obstruction | 8 | 15.3 (6.25–37.43) | 15.29 (64.11) | 9.57 (3.91) | 3.26 (1.11) |
| Herpes simplex reactivation | 8 | 30.59 (10.61–88.18) | 30.58 (98.11) | 13.68 (4.75) | 3.77 (1.25) |
| Clonus | 7 | 22.94 (8.05–65.42) | 22.94 (73.43) | 11.97 (4.2) | 3.58 (1.03) |
| Graves' disease | 7 | 6.98 (3–16.28) | 6.98 (27.5) | 5.59 (2.4) | 2.48 (0.66) |
| Troponin t increased | 7 | 5.74 (2.51–13.13) | 5.73 (21.89) | 4.79 (2.09) | 2.26 (0.55) |
| Infusion site erythema | 7 | 10.04 (4.13–24.4) | 10.03 (39.61) | 7.29 (3) | 2.86 (0.82) |
| Oesophageal disorder | 7 | 5.54 (2.43–12.64) | 5.54 (20.96) | 4.65 (2.04) | 2.22 (0.53) |
| Terminal agitation | 7 | 80.31 (16.68–386.61) | 80.28 (121.79) | 18.62 (3.87) | 4.22 (1.13) |
| Oesophagitis ulcerative | 7 | 9.45 (3.92–22.78) | 9.44 (37.44) | 6.98 (2.89) | 2.8 (0.8) |
| Lupus-like syndrome | 7 | 9.45 (3.92–22.78) | 9.44 (37.44) | 6.98 (2.89) | 2.8 (0.8) |
| Blood lactic acid increased | 7 | 8.03 (3.4–18.99) | 8.03 (31.9) | 6.21 (2.62) | 2.63 (0.73) |
| Chronic myeloid leukaemia | 7 | 12.35 (4.93–30.97) | 12.35 (47.47) | 8.38 (3.34) | 3.07 (0.89) |
| Vitritis | 6 | 45.89 (11.48–183.49) | 45.87 (87.79) | 15.96 (3.99) | 4 (0.88) |
| Shock symptom | 6 | 15.3 (5.44–42.98) | 15.29 (48.09) | 9.57 (3.41) | 3.26 (0.76) |
| Fanconi syndrome | 6 | 22.94 (7.4–71.15) | 22.94 (62.94) | 11.97 (3.86) | 3.58 (0.83) |
| Hyperphosphaturia | 6 | 34.42 (9.71–121.97) | 34.41 (77.84) | 14.36 (4.05) | 3.84 (0.87) |
| Autoimmune lung disease | 6 | 8.6 (3.37–21.99) | 8.6 (29.31) | 6.53 (2.55) | 2.71 (0.59) |
| Stasis dermatitis | 6 | 6.56 (2.65–16.24) | 6.55 (21.96) | 5.32 (2.15) | 2.41 (0.47) |
| Cytomegalovirus colitis | 6 | 6.26 (2.54–15.43) | 6.26 (20.82) | 5.13 (2.08) | 2.36 (0.45) |
| Haemorrhagic disorder | 6 | 13.77 (5–37.88) | 13.76 (44.38) | 8.98 (3.26) | 3.17 (0.74) |
| Paraneoplastic rash | 6 | 45.89 (11.48–183.49) | 45.87 (87.79) | 15.96 (3.99) | 4 (0.88) |
| Blood creatine phosphokinase abnormal | 6 | 9.18 (3.56–23.66) | 9.17 (31.22) | 6.84 (2.65) | 2.77 (0.61) |
| Genital tract inflammation | 6 | 68.83 (13.89–341.06) | 68.81 (100.24) | 17.95 (3.62) | 4.17 (0.89) |
| Glucocorticoid deficiency | 6 | 7.25 (2.89–18.14) | 7.24 (24.54) | 5.74 (2.29) | 2.52 (0.52) |
| Vascular device infection | 6 | 7.65 (3.04–19.27) | 7.65 (26) | 5.98 (2.38) | 2.58 (0.54) |
| Blood phosphorus abnormal | 6 | 19.67 (6.61–58.52) | 19.66 (57.22) | 11.05 (3.71) | 3.47 (0.81) |
| Stenosis | 5 | 12.75 (4.27–38.04) | 12.74 (34.78) | 8.55 (2.86) | 3.1 (0.49) |
| Amoebic colitis | 5 | 114.71 (13.4–981.96) | 114.68 (93.91) | 19.95 (2.33) | 4.32 (0.61) |
| Abdominal mass | 5 | 12.75 (4.27–38.04) | 12.74 (34.78) | 8.55 (2.86) | 3.1 (0.49) |
| Leg amputation | 5 | 11.47 (3.92–33.56) | 11.47 (31.85) | 7.98 (2.73) | 3 (0.47) |
| Punctate keratitis | 5 | 38.24 (9.14–160.01) | 38.23 (67.98) | 14.96 (3.58) | 3.9 (0.6) |
| Vestibular disorder | 5 | 11.47 (3.92–33.56) | 11.47 (31.85) | 7.98 (2.73) | 3 (0.47) |
| Occipital neuralgia | 5 | 28.68 (7.7–106.81) | 28.67 (59.35) | 13.3 (3.57) | 3.73 (0.59) |
| Hyperphosphataemia | 5 | 16.39 (5.2–51.64) | 16.38 (42.13) | 9.97 (3.17) | 3.32 (0.53) |
| Pseudomonal sepsis | 5 | 10.43 (3.62–30.02) | 10.43 (29.29) | 7.48 (2.6) | 2.9 (0.44) |
| Allergy test positive | 5 | 114.71 (13.4–981.96) | 114.68 (93.91) | 19.95 (2.33) | 4.32 (0.61) |
| Epiglottic oedema | 5 | 57.36 (11.13–295.66) | 57.34 (79.09) | 17.1 (3.32) | 4.1 (0.61) |
| Parvovirus b19 test positive | 5 | 114.71 (13.4–981.96) | 114.68 (93.91) | 19.95 (2.33) | 4.32 (0.61) |
| Myeloproliferative neoplasm | 5 | 9.56 (3.37–27.14) | 9.56 (27.04) | 7.04 (2.48) | 2.82 (0.42) |
| Exposure during pregnancy | 5 | 8.19 (2.95–22.75) | 8.19 (23.26) | 6.3 (2.27) | 2.66 (0.37) |
| Ear swelling | 4 | 10.2 (3.14–33.11) | 10.19 (22.96) | 7.37 (2.27) | 2.88 (0.15) |
| Scleroedema | 4 | 11.47 (3.45–38.1) | 11.47 (25.48) | 7.98 (2.4) | 3 (0.18) |
| Hyperplasia | 4 | 11.47 (3.45–38.1) | 11.47 (25.48) | 7.98 (2.4) | 3 (0.18) |
| Myopathy toxic | 4 | 30.59 (6.85–136.68) | 30.58 (49.06) | 13.68 (3.06) | 3.77 (0.26) |
| Wound necrosis | 4 | 22.94 (5.74–91.74) | 22.94 (41.96) | 11.97 (2.99) | 3.58 (0.25) |
| Trigger finger | 4 | 18.35 (4.93–68.35) | 18.35 (36.45) | 10.64 (2.86) | 3.41 (0.24) |
| Overlap syndrome | 4 | 13.11 (3.84–44.79) | 13.11 (28.47) | 8.7 (2.55) | 3.12 (0.2) |
| Pneumobilia | 4 | 22.94 (5.74–91.74) | 22.94 (41.96) | 11.97 (2.99) | 3.58 (0.25) |
| Endophthalmitis | 4 | 13.11 (3.84–44.79) | 13.11 (28.47) | 8.7 (2.55) | 3.12 (0.2) |
| Meningitis noninfective | 4 | 18.35 (4.93–68.35) | 18.35 (36.45) | 10.64 (2.86) | 3.41 (0.24) |
| Enterovesical fistula | 4 | 13.11 (3.84–44.79) | 13.11 (28.47) | 8.7 (2.55) | 3.12 (0.2) |
| Serratia infection | 4 | 45.88 (8.4–250.52) | 45.87 (58.53) | 15.96 (2.92) | 4 (0.26) |
| Premature delivery | 4 | 8.34 (2.66–26.2) | 8.34 (18.95) | 6.38 (2.03) | 2.67 (0.1) |
| Ocular myasthenia | 4 | 8.34 (2.66–26.2) | 8.34 (18.95) | 6.38 (2.03) | 2.67 (0.1) |
| Decreased eye contact | 4 | 91.77 (10.26–821.09) | 91.75 (71.81) | 19.15 (2.14) | 4.26 (0.25) |
| Fusarium infection | 4 | 30.59 (6.85–136.68) | 30.58 (49.06) | 13.68 (3.06) | 3.77 (0.26) |
| Klebsiella sepsis | 4 | 8.34 (2.66–26.2) | 8.34 (18.95) | 6.38 (2.03) | 2.67 (0.1) |
| Alpha haemolytic streptococcal infection | 4 | 18.35 (4.93–68.35) | 18.35 (36.45) | 10.64 (2.86) | 3.41 (0.24) |
| Vascular access site swelling | 4 | 91.77 (10.26–821.09) | 91.75 (71.81) | 19.15 (2.14) | 4.26 (0.25) |
| Type iii immune complex mediated reaction | 4 | 45.88 (8.4–250.52) | 45.87 (58.53) | 15.96 (2.92) | 4 (0.26) |
| Nephrogenic diabetes insipidus | 4 | 9.18 (2.88–29.26) | 9.17 (20.81) | 6.84 (2.14) | 2.77 (0.13) |
| Infusion related hypersensitivity reaction | 4 | 8.34 (2.66–26.2) | 8.34 (18.95) | 6.38 (2.03) | 2.67 (0.1) |

Supplementary **Table S9C**

**Disproportionality analysis of** carbo**platin-associated positive signals after excluding albumin-bound paclitaxel co-reported cases in the FAERS database**

| All qualifying carboplatin-associated preferred terms after excluding albumin-bound paclitaxel co-reported cases (n=29,021) | | | | | |
| --- | --- | --- | --- | --- | --- |
| PT | N | ROR (95%CI) | PRR(χ^2^) | EBGM(EBGM05) | IC(IC025) |
| Neutropenia | 532 | 3.08 (2.81–3.39) | 3.05 (616.52) | 2.71 (2.47) | 1.44 (1.3) |
| Pancytopenia | 524 | 6.65 (6–7.37) | 6.55 (1746.08) | 4.92 (4.44) | 2.3 (2.14) |
| Thrombocytopenia | 464 | 2.94 (2.66–3.24) | 2.91 (492.3) | 2.61 (2.36) | 1.38 (1.23) |
| Febrile neutropenia | 463 | 3.06 (2.77–3.38) | 3.02 (529.22) | 2.7 (2.44) | 1.43 (1.28) |
| Acute kidney injury | 385 | 2.94 (2.63–3.28) | 2.91 (410.15) | 2.61 (2.34) | 1.39 (1.22) |
| Leukopenia | 258 | 3.46 (3.03–3.97) | 3.44 (367.77) | 3 (2.62) | 1.59 (1.38) |
| Septic shock | 167 | 3.85 (3.25–4.56) | 3.83 (281.44) | 3.28 (2.77) | 1.71 (1.45) |
| Condition aggravated | 165 | 3.02 (2.56–3.57) | 3.01 (186.46) | 2.69 (2.27) | 1.43 (1.17) |
| Neutropenic sepsis | 145 | 7.5 (6.16–9.15) | 7.47 (551.38) | 5.39 (4.42) | 2.43 (2.11) |
| Therapy partial responder | 127 | 4.06 (3.34–4.94) | 4.05 (232.11) | 3.42 (2.82) | 1.78 (1.47) |
| Hyperthyroidism | 93 | 3.05 (2.44–3.81) | 3.04 (107.12) | 2.71 (2.17) | 1.44 (1.09) |
| Rash maculo-papular | 85 | 3.76 (2.97–4.77) | 3.75 (138.65) | 3.22 (2.54) | 1.69 (1.31) |
| Hepatic cytolysis | 85 | 3.57 (2.82–4.52) | 3.56 (127.83) | 3.09 (2.44) | 1.63 (1.25) |
| Agranulocytosis | 83 | 4.93 (3.85–6.31) | 4.92 (197.68) | 3.99 (3.11) | 2 (1.59) |
| Hypomagnesaemia | 80 | 3.77 (2.95–4.82) | 3.76 (131.12) | 3.23 (2.53) | 1.69 (1.3) |
| Anaphylactic shock | 80 | 4.86 (3.79–6.25) | 4.85 (187.18) | 3.94 (3.07) | 1.98 (1.57) |
| Disease recurrence | 74 | 10.32 (7.69–13.83) | 10.29 (375.27) | 6.61 (4.93) | 2.73 (2.22) |
| Therapeutic product effect incomplete | 70 | 5.46 (4.16–7.16) | 5.45 (188.79) | 4.3 (3.28) | 2.1 (1.65) |
| Polyneuropathy | 68 | 4.7 (3.58–6.16) | 4.69 (152.02) | 3.84 (2.93) | 1.94 (1.49) |
| Febrile bone marrow aplasia | 66 | 6.84 (5.12–9.13) | 6.82 (228.84) | 5.06 (3.79) | 2.34 (1.85) |
| Hypertransaminasaemia | 63 | 5.48 (4.11–7.3) | 5.47 (170.8) | 4.32 (3.24) | 2.11 (1.63) |
| Hepatocellular injury | 57 | 3.6 (2.7–4.81) | 3.6 (87.05) | 3.11 (2.33) | 1.64 (1.17) |
| Therapy non-responder | 56 | 3.85 (2.87–5.16) | 3.84 (94.7) | 3.28 (2.45) | 1.72 (1.24) |
| Posterior reversible encephalopathy syndrome | 47 | 4.77 (3.44–6.61) | 4.77 (107.35) | 3.89 (2.81) | 1.96 (1.41) |
| Toxic epidermal necrolysis | 47 | 3.52 (2.57–4.83) | 3.52 (69.23) | 3.06 (2.23) | 1.61 (1.09) |
| Acute myeloid leukaemia | 46 | 5.48 (3.92–7.67) | 5.48 (124.87) | 4.32 (3.09) | 2.11 (1.53) |
| Immune-mediated hypothyroidism | 43 | 6.1 (4.29–8.67) | 6.09 (131.84) | 4.67 (3.28) | 2.22 (1.61) |
| Lymphopenia | 42 | 4 (2.85–5.62) | 4 (75.37) | 3.39 (2.42) | 1.76 (1.19) |
| Toxic skin eruption | 39 | 3.79 (2.67–5.37) | 3.78 (64.39) | 3.24 (2.29) | 1.7 (1.11) |
| Autoimmune hepatitis | 37 | 3.62 (2.53–5.17) | 3.61 (56.84) | 3.12 (2.18) | 1.64 (1.05) |
| Renal tubular necrosis | 36 | 3.43 (2.39–4.92) | 3.43 (50.87) | 2.99 (2.09) | 1.58 (0.98) |
| Bicytopenia | 35 | 9.03 (5.96–13.68) | 9.02 (158.56) | 6.09 (4.02) | 2.61 (1.84) |
| Myelodysplastic syndrome | 34 | 5.09 (3.46–7.5) | 5.09 (84.4) | 4.09 (2.78) | 2.03 (1.35) |
| Prescribed overdose | 30 | 4.45 (2.97–6.68) | 4.45 (62.51) | 3.69 (2.46) | 1.88 (1.18) |
| Lichenoid keratosis | 30 | 3.42 (2.3–5.08) | 3.42 (42.13) | 2.98 (2.01) | 1.58 (0.92) |
| Congenital aplasia | 30 | 11.24 (7.03–17.95) | 11.23 (163.03) | 6.96 (4.36) | 2.8 (1.91) |
| Therapeutic response decreased | 30 | 3.77 (2.53–5.62) | 3.77 (49.3) | 3.24 (2.17) | 1.69 (1.02) |
| Tumour lysis syndrome | 28 | 3.7 (2.45–5.59) | 3.7 (44.63) | 3.18 (2.11) | 1.67 (0.97) |
| Angioedema | 27 | 4.42 (2.89–6.78) | 4.42 (55.78) | 3.67 (2.39) | 1.88 (1.13) |
| Acute coronary syndrome | 27 | 4.04 (2.65–6.17) | 4.04 (49.17) | 3.42 (2.24) | 1.77 (1.04) |
| Dermatitis exfoliative generalised | 27 | 7.58 (4.79–12.01) | 7.58 (104.02) | 5.44 (3.43) | 2.44 (1.59) |
| Renal tubular disorder | 26 | 6.29 (3.99–9.92) | 6.29 (82.57) | 4.78 (3.03) | 2.26 (1.42) |
| Dermatitis bullous | 26 | 3.97 (2.58–6.1) | 3.97 (46.08) | 3.37 (2.19) | 1.75 (1.01) |
| Neutropenic colitis | 26 | 7.05 (4.44–11.2) | 7.04 (93.13) | 5.17 (3.26) | 2.37 (1.51) |
| Immune-mediated hyperthyroidism | 23 | 16.44 (9.16–29.5) | 16.43 (162.96) | 8.54 (4.76) | 3.09 (1.95) |
| Squamous cell carcinoma of lung | 23 | 5.48 (3.41–8.81) | 5.48 (62.42) | 4.32 (2.69) | 2.11 (1.25) |
| Autoimmune haemolytic anaemia | 22 | 5.24 (3.23–8.49) | 5.24 (56.6) | 4.18 (2.58) | 2.06 (1.19) |
| Endocarditis | 20 | 7.49 (4.4–12.76) | 7.48 (76.11) | 5.39 (3.17) | 2.43 (1.41) |
| Autoimmune colitis | 20 | 4.19 (2.56–6.87) | 4.19 (38.37) | 3.52 (2.15) | 1.82 (0.94) |
| Contraindicated product administered | 18 | 14.9 (7.82–28.39) | 14.89 (119.76) | 8.13 (4.27) | 3.02 (1.73) |
| Acute generalised exanthematous pustulosis | 18 | 5.05 (2.97–8.6) | 5.05 (44.27) | 4.07 (2.39) | 2.02 (1.05) |
| Lip oedema | 16 | 12.58 (6.52–24.28) | 12.57 (94.69) | 7.43 (3.85) | 2.89 (1.56) |
| Extremity necrosis | 16 | 4.57 (2.62–7.98) | 4.57 (34.59) | 3.77 (2.16) | 1.91 (0.9) |
| Immune-mediated nephritis | 16 | 4.26 (2.45–7.41) | 4.26 (31.43) | 3.57 (2.05) | 1.83 (0.84) |
| Immune-mediated hypophysitis | 15 | 7.37 (3.99–13.61) | 7.37 (56.2) | 5.33 (2.89) | 2.42 (1.21) |
| Raynaud's phenomenon | 14 | 7.1 (3.78–13.35) | 7.1 (50.53) | 5.2 (2.77) | 2.38 (1.14) |
| Febrile infection | 14 | 6.11 (3.3–11.34) | 6.11 (43.1) | 4.68 (2.52) | 2.23 (1.04) |
| Kounis syndrome | 13 | 204.4 (26.74–1562.55) | 204.3 (187.86) | 15.52 (2.03) | 3.96 (1.81) |
| Myelitis transverse | 13 | 8.18 (4.18–15.98) | 8.17 (53.84) | 5.72 (2.93) | 2.52 (1.17) |
| Jugular vein thrombosis | 13 | 5.38 (2.86–10.1) | 5.38 (34.51) | 4.26 (2.27) | 2.09 (0.9) |
| Papilloedema | 12 | 5.72 (2.95–11.07) | 5.71 (34.23) | 4.46 (2.3) | 2.16 (0.89) |
| Demyelinating polyneuropathy | 12 | 8.2 (4.08–16.49) | 8.2 (49.85) | 5.73 (2.85) | 2.52 (1.11) |
| Progressive multifocal leukoencephalopathy | 12 | 6.09 (3.13–11.85) | 6.08 (36.75) | 4.66 (2.4) | 2.22 (0.93) |
| Cutaneous lupus erythematosus | 11 | 6.92 (3.4–14.06) | 6.91 (38.65) | 5.11 (2.51) | 2.35 (0.94) |
| Peripheral sensorimotor neuropathy | 11 | 7.52 (3.66–15.43) | 7.52 (42.04) | 5.41 (2.64) | 2.44 (0.99) |
| Autoimmune hypothyroidism | 11 | 9.1 (4.33–19.13) | 9.1 (50.22) | 6.13 (2.92) | 2.62 (1.09) |
| Tracheo-oesophageal fistula | 11 | 5.58 (2.8–11.1) | 5.58 (30.5) | 4.38 (2.2) | 2.13 (0.81) |
| Sarcoidosis | 10 | 5.42 (2.64–11.12) | 5.42 (26.8) | 4.29 (2.09) | 2.1 (0.71) |
| Macular oedema | 10 | 5.24 (2.56–10.72) | 5.24 (25.72) | 4.18 (2.04) | 2.06 (0.69) |
| Rash morbilliform | 10 | 5.24 (2.56–10.72) | 5.24 (25.72) | 4.18 (2.04) | 2.06 (0.69) |
| Troponin t increased | 10 | 6.29 (3.02–13.09) | 6.29 (31.75) | 4.78 (2.29) | 2.26 (0.81) |
| Optic ischaemic neuropathy | 10 | 9.25 (4.23–20.2) | 9.24 (46.3) | 6.19 (2.83) | 2.63 (1.01) |
| Large intestine infection | 10 | 9.25 (4.23–20.2) | 9.24 (46.3) | 6.19 (2.83) | 2.63 (1.01) |
| Vascular purpura | 9 | 9.43 (4.13–21.55) | 9.43 (42.39) | 6.27 (2.74) | 2.65 (0.92) |
| Palmar erythema | 9 | 6.43 (2.96–13.97) | 6.43 (29.28) | 4.85 (2.23) | 2.28 (0.73) |
| Blood culture positive | 9 | 5.66 (2.64–12.12) | 5.66 (25.38) | 4.42 (2.07) | 2.15 (0.66) |
| Autoimmune nephritis | 9 | 5.66 (2.64–12.12) | 5.66 (25.38) | 4.42 (2.07) | 2.15 (0.66) |
| Infusion site erythema | 9 | 10.11 (4.37–23.35) | 10.1 (44.93) | 6.54 (2.83) | 2.71 (0.95) |
| Necrotising myositis | 9 | 10.88 (4.65–25.46) | 10.88 (47.72) | 6.84 (2.92) | 2.77 (0.98) |
| Magnetic resonance imaging abnormal | 9 | 14.15 (5.75–34.82) | 14.14 (57.86) | 7.92 (3.22) | 2.99 (1.07) |
| Vascular device infection | 9 | 9.43 (4.13–21.55) | 9.43 (42.39) | 6.27 (2.74) | 2.65 (0.92) |
| Oesophagitis ulcerative | 8 | 7.86 (3.36–18.37) | 7.86 (31.92) | 5.57 (2.38) | 2.48 (0.73) |
| Intestinal pseudo-obstruction | 8 | 10.48 (4.28–25.64) | 10.48 (41.15) | 6.69 (2.73) | 2.74 (0.85) |
| Exposure during pregnancy | 8 | 11.43 (4.6–28.42) | 11.43 (44.08) | 7.04 (2.83) | 2.82 (0.88) |
| Glucocorticoid deficiency | 8 | 7.4 (3.19–17.14) | 7.4 (30.09) | 5.35 (2.31) | 2.42 (0.7) |
| Herpes simplex reactivation | 8 | 20.96 (7.27–60.41) | 20.95 (65.15) | 9.55 (3.31) | 3.26 (1.03) |
| Clonus | 7 | 15.72 (5.51–44.82) | 15.72 (48.23) | 8.36 (2.93) | 3.06 (0.81) |
| Endophthalmitis | 7 | 27.51 (8.05–93.98) | 27.5 (65.01) | 10.64 (3.11) | 3.41 (0.9) |
| Actinic keratosis | 7 | 11 (4.19–28.91) | 11 (37.44) | 6.88 (2.62) | 2.78 (0.72) |
| Terminal agitation | 7 | 55.02 (11.43–264.86) | 55 (82.48) | 13 (2.7) | 3.7 (0.95) |
| Lupus-like syndrome | 7 | 6.47 (2.68–15.61) | 6.47 (22.94) | 4.88 (2.02) | 2.29 (0.51) |
| Myeloproliferative neoplasm | 7 | 11 (4.19–28.91) | 11 (37.44) | 6.88 (2.62) | 2.78 (0.72) |
| Chronic myeloid leukaemia | 7 | 8.46 (3.38–21.22) | 8.46 (29.94) | 5.85 (2.33) | 2.55 (0.63) |
| Vitritis | 6 | 31.44 (7.86–125.71) | 31.43 (58.93) | 11.14 (2.79) | 3.48 (0.71) |
| Shock symptom | 6 | 10.48 (3.73–29.44) | 10.48 (30.86) | 6.69 (2.38) | 2.74 (0.53) |
| Fanconi syndrome | 6 | 15.72 (5.07–48.74) | 15.72 (41.34) | 8.36 (2.7) | 3.06 (0.62) |
| Hyperphosphaturia | 6 | 23.58 (6.65–83.56) | 23.57 (51.88) | 10.03 (2.83) | 3.33 (0.68) |
| Pseudomonal sepsis | 6 | 9.43 (3.43–25.95) | 9.43 (28.26) | 6.27 (2.28) | 2.65 (0.5) |
| Haemorrhagic disorder | 6 | 9.43 (3.43–25.95) | 9.43 (28.26) | 6.27 (2.28) | 2.65 (0.5) |
| Paraneoplastic rash | 6 | 31.44 (7.86–125.71) | 31.43 (58.93) | 11.14 (2.79) | 3.48 (0.71) |
| Antinuclear antibody | 6 | 31.44 (7.86–125.71) | 31.43 (58.93) | 11.14 (2.79) | 3.48 (0.71) |
| Genital tract inflammation | 6 | 47.16 (9.52–233.66) | 47.15 (67.75) | 12.54 (2.53) | 3.65 (0.73) |
| Lung carcinoma cell type unspecified stage iii | 6 | 31.44 (7.86–125.71) | 31.43 (58.93) | 11.14 (2.79) | 3.48 (0.71) |
| Infusion related hypersensitivity reaction | 6 | 10.48 (3.73–29.44) | 10.48 (30.86) | 6.69 (2.38) | 2.74 (0.53) |
| Idiopathic intracranial hypertension | 6 | 15.72 (5.07–48.74) | 15.72 (41.34) | 8.36 (2.7) | 3.06 (0.62) |
| Blood phosphorus abnormal | 6 | 13.47 (4.53–40.09) | 13.47 (37.3) | 7.71 (2.59) | 2.95 (0.59) |
| Immune-mediated encephalopathy | 6 | 7.25 (2.76–19.09) | 7.25 (22.13) | 5.28 (2.01) | 2.4 (0.4) |
| Intensive care | 5 | 9.82 (3.21–30.03) | 9.82 (24.38) | 6.43 (2.1) | 2.68 (0.3) |
| Punctate keratitis | 5 | 26.2 (6.26–109.63) | 26.19 (45.43) | 10.45 (2.5) | 3.39 (0.44) |
| Fournier's gangrene | 5 | 19.65 (5.28–73.17) | 19.64 (39.32) | 9.29 (2.49) | 3.22 (0.42) |
| Occipital neuralgia | 5 | 19.65 (5.28–73.17) | 19.64 (39.32) | 9.29 (2.49) | 3.22 (0.42) |
| Hyperphosphataemia | 5 | 11.23 (3.56–35.38) | 11.23 (27.17) | 6.96 (2.21) | 2.8 (0.33) |
| Epidermolysis bullosa | 5 | 9.82 (3.21–30.03) | 9.82 (24.38) | 6.43 (2.1) | 2.68 (0.3) |
| Epiglottic oedema | 5 | 39.3 (7.62–202.56) | 39.29 (53.31) | 11.94 (2.32) | 3.58 (0.46) |
| Escherichia test positive | 5 | 9.82 (3.21–30.03) | 9.82 (24.38) | 6.43 (2.1) | 2.68 (0.3) |
| Subclavian artery occlusion | 5 | 26.2 (6.26–109.63) | 26.19 (45.43) | 10.45 (2.5) | 3.39 (0.44) |
| Chronic myelomonocytic leukaemia | 5 | 11.23 (3.56–35.38) | 11.23 (27.17) | 6.96 (2.21) | 2.8 (0.33) |
| Oligoastrocytoma | 4 | 20.96 (4.69–93.64) | 20.95 (32.58) | 9.55 (2.14) | 3.26 (0.11) |
| Myopathy toxic | 4 | 20.96 (4.69–93.64) | 20.95 (32.58) | 9.55 (2.14) | 3.26 (0.11) |
| Wound necrosis | 4 | 15.72 (3.93–62.85) | 15.72 (27.56) | 8.36 (2.09) | 3.06 (0.09) |
| Pneumobilia | 4 | 15.72 (3.93–62.85) | 15.72 (27.56) | 8.36 (2.09) | 3.06 (0.09) |
| Gastritis bacterial | 4 | 31.44 (5.76–171.64) | 31.43 (39.28) | 11.14 (2.04) | 3.48 (0.13) |
| Serratia infection | 4 | 31.44 (5.76–171.64) | 31.43 (39.28) | 11.14 (2.04) | 3.48 (0.13) |
| Fusarium infection | 4 | 20.96 (4.69–93.64) | 20.95 (32.58) | 9.55 (2.14) | 3.26 (0.11) |
| Type iii immune complex mediated reaction | 4 | 31.44 (5.76–171.64) | 31.43 (39.28) | 11.14 (2.04) | 3.48 (0.13) |

Supplementary Table S10A

Disproportionality analysis of cisplatin-associated positive signals after excluding pemetrexed co-reported cases in the FAERS database

| All qualifying cisplatin-associated preferred terms after excluding pemetrexed co-reported cases (n=3,458) | | | | | |
| --- | --- | --- | --- | --- | --- |
| PT | N | ROR (95%CI) | PRR(χ^2^) | EBGM(EBGM05) | IC(IC025) |
| Neutropenia | 95 | 4.25 (3.45–5.22) | 4.16 (222.79) | 4.07 (3.31) | 2.02 (1.68) |
| Febrile neutropenia | 89 | 4.55 (3.67–5.64) | 4.46 (232.79) | 4.35 (3.51) | 2.12 (1.76) |
| Acute kidney injury | 61 | 3.58 (2.77–4.63) | 3.54 (108.9) | 3.48 (2.69) | 1.8 (1.37) |
| Pancytopenia | 51 | 4.15 (3.14–5.5) | 4.11 (116.83) | 4.02 (3.03) | 2.01 (1.51) |
| Pulmonary embolism | 49 | 3.56 (2.67–4.73) | 3.52 (86.57) | 3.46 (2.6) | 1.79 (1.3) |
| Leukopenia | 39 | 3.92 (2.85–5.4) | 3.89 (81.65) | 3.81 (2.77) | 1.93 (1.37) |
| Neutropenic sepsis | 33 | 11.12 (7.79–15.87) | 11.02 (278.94) | 10.29 (7.21) | 3.36 (2.5) |
| Hyponatraemia | 32 | 3.56 (2.51–5.07) | 3.54 (57.03) | 3.48 (2.44) | 1.8 (1.18) |
| Renal failure | 30 | 2.97 (2.07–4.27) | 2.95 (38.06) | 2.91 (2.03) | 1.54 (0.93) |
| Septic shock | 30 | 5.12 (3.55–7.38) | 5.08 (95.11) | 4.94 (3.43) | 2.3 (1.6) |
| Aortic thrombosis | 28 | 109.21 (66.57–179.16) | 108.33 (1675.11) | 61.37 (37.41) | 5.94 (3.66) |
| Toxicity to various agents | 26 | 4.82 (3.25–7.13) | 4.79 (75.5) | 4.66 (3.15) | 2.22 (1.47) |
| Oesophagitis | 23 | 6.28 (4.13–9.55) | 6.24 (97.07) | 6.02 (3.96) | 2.59 (1.71) |
| Nephropathy toxic | 19 | 9.78 (6.13–15.59) | 9.73 (139.18) | 9.16 (5.75) | 3.2 (2.03) |
| Acute myocardial infarction | 16 | 7.06 (4.27–11.68) | 7.03 (78.84) | 6.74 (4.07) | 2.75 (1.61) |
| Cytomegalovirus oesophagitis | 15 | 262.29 (111.13–619.06) | 261.16 (1352.22) | 91.49 (38.76) | 6.52 (2.84) |
| Multiple organ dysfunction syndrome | 15 | 4.55 (2.72–7.61) | 4.53 (40.03) | 4.42 (2.64) | 2.14 (1.13) |
| Renal salt-wasting syndrome | 14 | 244.74 (102.6–583.77) | 243.75 (1230.8) | 89.27 (37.43) | 6.48 (2.73) |
| Hypomagnesaemia | 13 | 4.53 (2.6–7.88) | 4.52 (34.49) | 4.41 (2.53) | 2.14 (1.04) |
| Inappropriate antidiuretic hormone secretion | 12 | 6.9 (3.86–12.33) | 6.88 (57.48) | 6.6 (3.69) | 2.72 (1.38) |
| Tinnitus | 11 | 7.08 (3.86–12.99) | 7.06 (54.49) | 6.77 (3.69) | 2.76 (1.33) |
| Orthostatic hypotension | 11 | 11.47 (6.2–21.22) | 11.43 (96.82) | 10.64 (5.75) | 3.41 (1.69) |
| Gastrointestinal toxicity | 11 | 7.05 (3.84–12.93) | 7.03 (54.17) | 6.74 (3.67) | 2.75 (1.33) |
| Renal infarct | 10 | 48.17 (23.46–98.91) | 48.03 (342.45) | 35.97 (17.52) | 5.17 (2.11) |
| Renal tubular necrosis | 10 | 7.31 (3.87–13.82) | 7.29 (51.62) | 6.98 (3.69) | 2.8 (1.28) |
| Cyanosis | 9 | 7.52 (3.84–14.73) | 7.51 (48.18) | 7.17 (3.67) | 2.84 (1.21) |
| Arterial thrombosis | 9 | 26.18 (12.84–53.4) | 26.12 (183.09) | 22.15 (10.86) | 4.47 (1.83) |
| Posterior reversible encephalopathy syndrome | 9 | 6.51 (3.33–12.71) | 6.5 (39.99) | 6.25 (3.2) | 2.64 (1.09) |
| Lower respiratory tract infection | 9 | 4.62 (2.38–8.98) | 4.61 (24.63) | 4.49 (2.31) | 2.17 (0.8) |
| Cerebral venous thrombosis | 8 | 101.53 (40.81–252.58) | 101.3 (459.97) | 59.07 (23.74) | 5.88 (1.82) |
| Neurotoxicity | 7 | 5.34 (2.51–11.36) | 5.33 (23.71) | 5.17 (2.43) | 2.37 (0.72) |
| Bronchospasm | 7 | 4.67 (2.2–9.93) | 4.67 (19.51) | 4.55 (2.14) | 2.18 (0.61) |
| Peripheral artery thrombosis | 7 | 25.05 (11.2–56.04) | 25 (136.75) | 21.35 (9.54) | 4.42 (1.48) |
| Presyncope | 6 | 9.2 (4.02–21.03) | 9.18 (41.06) | 8.68 (3.8) | 3.12 (0.91) |
| Hepatitis b | 6 | 16.74 (7.17–39.07) | 16.71 (79.16) | 15.03 (6.44) | 3.91 (1.16) |
| Hypovolaemia | 6 | 9.2 (4.02–21.03) | 9.18 (41.06) | 8.68 (3.8) | 3.12 (0.91) |
| Agitation | 6 | 5.69 (2.51–12.89) | 5.69 (22.26) | 5.5 (2.43) | 2.46 (0.62) |
| Splenic infarction | 6 | 25.37 (10.62–60.58) | 25.32 (118.63) | 21.58 (9.04) | 4.43 (1.26) |
| Type i hypersensitivity | 6 | 32.2 (13.24–78.27) | 32.14 (147.11) | 26.3 (10.82) | 4.72 (1.3) |
| Arterial occlusive disease | 6 | 25.37 (10.62–60.58) | 25.32 (118.63) | 21.58 (9.04) | 4.43 (1.26) |
| Empyema | 5 | 10.57 (4.25–26.24) | 10.55 (40.19) | 9.88 (3.98) | 3.3 (0.76) |
| Intestinal ischaemia | 5 | 9.96 (4.02–24.7) | 9.95 (37.57) | 9.35 (3.77) | 3.23 (0.74) |
| Pulmonary nocardiosis | 5 | 99.63 (31.61–314.07) | 99.49 (284.38) | 58.45 (18.54) | 5.87 (1.03) |
| Hypoparathyroidism | 5 | 49.82 (17.93–138.38) | 49.74 (175.98) | 36.92 (13.29) | 5.21 (1.05) |
| Dilated cardiomyopathy | 5 | 16.22 (6.42–40.97) | 16.2 (63.87) | 14.61 (5.78) | 3.87 (0.9) |
| Peripheral ischaemia | 5 | 5.81 (2.37–14.22) | 5.8 (19.09) | 5.61 (2.29) | 2.49 (0.45) |
| Neutropenic colitis | 5 | 8.83 (3.57–21.81) | 8.82 (32.59) | 8.35 (3.38) | 3.06 (0.68) |
| Tonsillitis | 4 | 30.99 (10.48–91.61) | 30.95 (94.86) | 25.51 (8.63) | 4.67 (0.68) |
| Kaposi's sarcoma | 4 | 139.45 (34.86–557.8) | 139.29 (274.59) | 70.14 (17.53) | 6.13 (0.62) |
| Granulocytopenia | 4 | 6.89 (2.52–18.8) | 6.88 (19.15) | 6.6 (2.42) | 2.72 (0.3) |
| Bicytopenia | 4 | 6.06 (2.23–16.5) | 6.06 (16.18) | 5.85 (2.15) | 2.55 (0.24) |
| Embolism venous | 4 | 8.08 (2.95–22.16) | 8.07 (23.44) | 7.69 (2.8) | 2.94 (0.38) |
| Diastolic dysfunction | 4 | 26.56 (9.11–77.42) | 26.53 (82.55) | 22.45 (7.7) | 4.49 (0.67) |
| Radiation oesophagitis | 4 | 6.89 (2.52–18.8) | 6.88 (19.15) | 6.6 (2.42) | 2.72 (0.3) |
| Necrosis ischaemic | 4 | 557.78 (62.33–4991.92) | 557.14 (444.12) | 112.23 (12.54) | 6.81 (0.51) |
| Ulcer haemorrhage | 4 | 15.49 (5.51–43.55) | 15.48 (48.75) | 14.03 (4.99) | 3.81 (0.58) |
| Recall phenomenon | 4 | 13.94 (4.99–38.99) | 13.93 (43.64) | 12.75 (4.56) | 3.67 (0.56) |
| Embolism arterial | 4 | 10.73 (3.88–29.67) | 10.71 (32.72) | 10.02 (3.62) | 3.32 (0.48) |
| Coronary artery thrombosis | 4 | 39.84 (13.11–121.1) | 39.8 (117.67) | 31.17 (10.26) | 4.96 (0.69) |
| Generalised tonic-clonic seizure | 4 | 6.48 (2.38–17.68) | 6.48 (17.71) | 6.23 (2.29) | 2.64 (0.27) |
| Salmonellosis | 3 | 104.55 (23.39–467.34) | 104.46 (175.67) | 60.12 (13.45) | 5.91 (0.15) |
| Myopericarditis | 3 | 19.01 (5.69–63.54) | 18.99 (45) | 16.83 (5.04) | 4.07 (0.2) |
| Pulse absent | 3 | 9.29 (2.89–29.92) | 9.29 (20.8) | 8.77 (2.72) | 3.13 (0.07) |
| Portal vein thrombosis | 3 | 13.94 (4.25–45.7) | 13.93 (32.73) | 12.75 (3.89) | 3.67 (0.16) |
| Hepatic infarction | 3 | 139.41 (28.13–690.95) | 139.29 (205.94) | 70.14 (14.15) | 6.13 (0.12) |
| Klebsiella infection | 3 | 15.49 (4.7–51.08) | 15.48 (36.56) | 14.03 (4.25) | 3.81 (0.18) |
| Oesophageal fistula | 3 | 12.67 (3.88–41.34) | 12.66 (29.54) | 11.69 (3.58) | 3.55 (0.14) |
| Vaginitis gardnerella | 3 | 418.22 (43.49–4021.71) | 417.86 (311.9) | 105.21 (10.94) | 6.72 (0.01) |
| Retinal artery occlusion | 3 | 32.17 (9.16–112.94) | 32.14 (73.55) | 26.3 (7.49) | 4.72 (0.23) |
| Subclavian vein thrombosis | 3 | 13.94 (4.25–45.7) | 13.93 (32.73) | 12.75 (3.89) | 3.67 (0.16) |
| Cd4 lymphocytes decreased | 3 | 83.64 (19.98–350.14) | 83.57 (152.97) | 52.61 (12.57) | 5.72 (0.18) |

Supplementary **Table S10B**

**Disproportionality analysis of** cisplatin**-associated positive signals after excluding paclitaxel co-reported cases in the FAERS database**

| All qualifying cisplatin-associated preferred terms after excluding paclitaxel co-reported cases (n=5,076) | | | | | |
| --- | --- | --- | --- | --- | --- |
| PT | N | ROR (95%CI) | PRR(χ^2^) | EBGM(EBGM05) | IC(IC025) |
| Neutropenia | 141 | 4.34 (3.66–5.16) | 4.25 (337.88) | 4.11 (3.47) | 2.04 (1.76) |
| Febrile neutropenia | 102 | 3.54 (2.9–4.32) | 3.49 (175.45) | 3.4 (2.78) | 1.76 (1.44) |
| Acute kidney injury | 97 | 3.93 (3.21–4.83) | 3.88 (200.09) | 3.77 (3.07) | 1.91 (1.57) |
| Thrombocytopenia | 79 | 2.61 (2.08–3.26) | 2.58 (74.91) | 2.54 (2.03) | 1.34 (0.99) |
| Pancytopenia | 72 | 4.03 (3.18–5.11) | 3.98 (154.99) | 3.86 (3.05) | 1.95 (1.55) |
| Pulmonary embolism | 61 | 3.02 (2.34–3.9) | 2.99 (78.82) | 2.93 (2.27) | 1.55 (1.13) |
| Leukopenia | 60 | 4.16 (3.21–5.4) | 4.12 (136.44) | 3.99 (3.08) | 2 (1.55) |
| Renal failure | 45 | 3.06 (2.27–4.12) | 3.04 (59.85) | 2.98 (2.21) | 1.57 (1.08) |
| Hyponatraemia | 38 | 2.88 (2.08–3.98) | 2.87 (44.99) | 2.81 (2.03) | 1.49 (0.95) |
| Septic shock | 36 | 4.19 (3–5.86) | 4.17 (83.3) | 4.04 (2.89) | 2.01 (1.41) |
| Neutropenic sepsis | 33 | 7.53 (5.27–10.74) | 7.48 (171.94) | 7.01 (4.91) | 2.81 (2.06) |
| Aortic thrombosis | 29 | 78.8 (48.13–129.01) | 78.36 (1211.33) | 43.3 (26.45) | 5.44 (3.52) |
| Oesophagitis | 28 | 5.24 (3.58–7.67) | 5.21 (90.44) | 4.99 (3.41) | 2.32 (1.58) |
| Toxicity to various agents | 28 | 3.53 (2.42–5.15) | 3.51 (48.58) | 3.42 (2.34) | 1.77 (1.11) |
| Nephropathy toxic | 25 | 8.93 (5.92–13.46) | 8.89 (160.07) | 8.21 (5.44) | 3.04 (2.09) |
| Acute myocardial infarction | 24 | 7.38 (4.86–11.18) | 7.35 (122.15) | 6.89 (4.54) | 2.78 (1.88) |
| Bone marrow failure | 23 | 3.41 (2.25–5.18) | 3.4 (37.74) | 3.32 (2.19) | 1.73 (1) |
| Renal tubular necrosis | 19 | 9.91 (6.17–15.9) | 9.87 (137.24) | 9.03 (5.63) | 3.18 (2.01) |
| Multiple organ dysfunction syndrome | 19 | 3.94 (2.49–6.24) | 3.93 (39.92) | 3.81 (2.41) | 1.93 (1.08) |
| Hypomagnesaemia | 17 | 4.06 (2.5–6.6) | 4.05 (37.47) | 3.92 (2.41) | 1.97 (1.06) |
| Renal salt-wasting syndrome | 16 | 252.98 (98.95–646.77) | 252.18 (1091.77) | 69.5 (27.19) | 6.12 (2.85) |
| Gastrointestinal toxicity | 16 | 7.12 (4.28–11.85) | 7.1 (78.09) | 6.68 (4.01) | 2.74 (1.59) |
| Cytomegalovirus oesophagitis | 15 | 177.84 (75.37–419.64) | 177.32 (914.77) | 62.33 (26.41) | 5.96 (2.74) |
| Tinnitus | 13 | 5.73 (3.27–10.04) | 5.72 (47.75) | 5.45 (3.11) | 2.45 (1.25) |
| Inappropriate antidiuretic hormone secretion | 13 | 5.09 (2.91–8.9) | 5.08 (40.45) | 4.87 (2.79) | 2.28 (1.13) |
| Posterior reversible encephalopathy syndrome | 13 | 6.52 (3.71–11.44) | 6.5 (56.69) | 6.15 (3.5) | 2.62 (1.37) |
| Orthostatic hypotension | 12 | 8.55 (4.73–15.45) | 8.53 (73.21) | 7.91 (4.38) | 2.98 (1.53) |
| Aphasia | 11 | 4.09 (2.23–7.48) | 4.08 (24.53) | 3.95 (2.16) | 1.98 (0.81) |
| Hypoacusis | 11 | 4.05 (2.22–7.42) | 4.05 (24.22) | 3.92 (2.14) | 1.97 (0.8) |
| Renal infarct | 11 | 37.23 (18.53–74.82) | 37.15 (277.85) | 26.96 (13.41) | 4.75 (2.13) |
| Lower respiratory tract infection | 11 | 3.86 (2.11–7.06) | 3.85 (22.34) | 3.74 (2.05) | 1.9 (0.75) |
| Arterial thrombosis | 10 | 20.16 (10.18–39.92) | 20.12 (149.85) | 16.77 (8.47) | 4.07 (1.83) |
| Capillary leak syndrome | 10 | 14.8 (7.6–28.84) | 14.78 (111.1) | 12.91 (6.63) | 3.69 (1.69) |
| Peripheral artery thrombosis | 10 | 26.32 (13.05–53.06) | 26.27 (190.25) | 20.78 (10.3) | 4.38 (1.92) |
| Febrile bone marrow aplasia | 10 | 4.55 (2.41–8.59) | 4.55 (26.41) | 4.38 (2.32) | 2.13 (0.85) |
| Cyanosis | 9 | 5.1 (2.61–9.99) | 5.1 (28.13) | 4.89 (2.5) | 2.29 (0.87) |
| Neurotoxicity | 9 | 4.71 (2.41–9.2) | 4.7 (25) | 4.53 (2.32) | 2.18 (0.8) |
| Presyncope | 9 | 9.69 (4.88–19.25) | 9.67 (63.5) | 8.87 (4.46) | 3.15 (1.34) |
| Enteritis | 9 | 4.61 (2.36–9) | 4.6 (24.2) | 4.43 (2.27) | 2.15 (0.78) |
| Melanoderma | 9 | 77.51 (32.11–187.13) | 77.37 (373.18) | 43.01 (17.81) | 5.43 (1.93) |
| Bronchospasm | 9 | 4.12 (2.11–8.03) | 4.11 (20.32) | 3.98 (2.04) | 1.99 (0.68) |
| Bicytopenia | 9 | 9.8 (4.93–19.47) | 9.78 (64.33) | 8.96 (4.51) | 3.16 (1.35) |
| Arterial occlusive disease | 9 | 28.42 (13.49–59.89) | 28.37 (182.82) | 22.05 (10.47) | 4.46 (1.8) |
| Splenic infarction | 8 | 24.44 (11.23–53.2) | 24.4 (142.74) | 19.6 (9.01) | 4.29 (1.6) |
| Peripheral ischaemia | 8 | 6.47 (3.16–13.26) | 6.47 (34.61) | 6.12 (2.99) | 2.61 (0.96) |
| Cerebral venous thrombosis | 8 | 68.88 (27.7–171.33) | 68.78 (309.36) | 40.24 (16.18) | 5.33 (1.74) |
| Metabolic acidosis | 7 | 5.43 (2.53–11.64) | 5.43 (23.91) | 5.19 (2.42) | 2.37 (0.71) |
| Ototoxicity | 6 | 17.75 (7.42–42.47) | 17.73 (79.78) | 15.09 (6.31) | 3.92 (1.13) |
| Hepatitis b | 6 | 11.36 (4.87–26.51) | 11.35 (50.56) | 10.24 (4.39) | 3.36 (0.98) |
| Hypovolaemia | 6 | 6.24 (2.73–14.26) | 6.24 (24.75) | 5.91 (2.59) | 2.56 (0.66) |
| Ileus paralytic | 6 | 6.6 (2.89–15.12) | 6.6 (26.64) | 6.23 (2.72) | 2.64 (0.7) |
| Radiation oesophagitis | 6 | 7.19 (3.13–16.5) | 7.18 (29.68) | 6.75 (2.94) | 2.75 (0.75) |
| Arteriospasm coronary | 6 | 13.52 (5.75–31.83) | 13.51 (60.82) | 11.95 (5.08) | 3.58 (1.04) |
| Neutropenic colitis | 6 | 7.28 (3.17–16.71) | 7.27 (30.16) | 6.83 (2.97) | 2.77 (0.75) |
| Empyema | 5 | 7.17 (2.89–17.81) | 7.16 (24.66) | 6.73 (2.71) | 2.75 (0.55) |
| Splenic rupture | 5 | 47.33 (16.17–138.52) | 47.28 (151.02) | 31.86 (10.88) | 4.99 (0.98) |
| Intestinal ischaemia | 5 | 6.76 (2.73–16.76) | 6.75 (22.88) | 6.37 (2.57) | 2.67 (0.52) |
| Pulmonary nocardiosis | 5 | 67.61 (21.45–213.11) | 67.55 (191.23) | 39.82 (12.63) | 5.32 (0.98) |
| Hypoparathyroidism | 5 | 33.81 (12.17–93.89) | 33.77 (117.18) | 25.15 (9.06) | 4.65 (0.97) |
| Type i hypersensitivity | 5 | 17.53 (6.75–45.54) | 17.51 (65.69) | 14.93 (5.75) | 3.9 (0.88) |
| Dilated cardiomyopathy | 5 | 11.01 (4.36–27.8) | 11 (40.71) | 9.96 (3.94) | 3.32 (0.74) |
| Hypercreatininaemia | 5 | 10.52 (4.17–26.51) | 10.51 (38.72) | 9.56 (3.79) | 3.26 (0.72) |
| Idiopathic pulmonary fibrosis | 5 | 9.86 (3.92–24.78) | 9.85 (36.01) | 9.02 (3.59) | 3.17 (0.7) |
| Tonsillitis | 4 | 21.03 (7.12–62.16) | 21.02 (62.39) | 17.38 (5.88) | 4.12 (0.59) |
| Kaposi's sarcoma | 4 | 94.64 (23.66–378.54) | 94.57 (185.16) | 47.78 (11.95) | 5.58 (0.58) |
| Diastolic dysfunction | 4 | 18.03 (6.19–52.53) | 18.01 (53.99) | 15.29 (5.25) | 3.93 (0.57) |
| Necrosis ischaemic | 4 | 378.57 (42.3–3387.77) | 378.27 (301.02) | 76.45 (8.54) | 6.26 (0.49) |
| Skin hyperpigmentation | 4 | 6.31 (2.29–17.36) | 6.3 (16.74) | 5.97 (2.17) | 2.58 (0.23) |
| Hypertensive crisis | 4 | 6.01 (2.19–16.51) | 6 (15.69) | 5.71 (2.08) | 2.51 (0.21) |
| Ulcer haemorrhage | 4 | 10.52 (3.74–29.55) | 10.51 (30.97) | 9.56 (3.4) | 3.26 (0.44) |
| Recall phenomenon | 4 | 9.46 (3.38–26.46) | 9.46 (27.5) | 8.69 (3.11) | 3.12 (0.4) |
| Embolism arterial | 4 | 7.28 (2.63–20.13) | 7.27 (20.1) | 6.83 (2.47) | 2.77 (0.3) |
| Subclavian vein thrombosis | 4 | 13.05 (4.59–37.14) | 13.04 (39.09) | 11.58 (4.07) | 3.53 (0.5) |
| Coronary artery thrombosis | 4 | 27.04 (8.9–82.18) | 27.02 (77.96) | 21.24 (6.99) | 4.41 (0.61) |
| Diabetes mellitus inadequate control | 4 | 6.76 (2.45–18.65) | 6.75 (18.31) | 6.37 (2.31) | 2.67 (0.27) |
| Drug monitoring procedure incorrectly performed | 4 | 378.57 (42.3–3387.77) | 378.27 (301.02) | 76.45 (8.54) | 6.26 (0.49) |
| Contraindicated product administered | 4 | 11.47 (4.06–32.39) | 11.46 (34.07) | 10.33 (3.66) | 3.37 (0.47) |
| Fibrosis | 3 | 13.52 (4.03–45.33) | 13.51 (30.41) | 11.95 (3.56) | 3.58 (0.11) |
| Salmonellosis | 3 | 70.97 (15.88–317.18) | 70.93 (118.19) | 40.96 (9.16) | 5.36 (0.12) |
| Myopericarditis | 3 | 12.9 (3.86–43.12) | 12.9 (28.97) | 11.47 (3.43) | 3.52 (0.1) |
| Formication | 3 | 17.74 (5.17–60.91) | 17.73 (39.89) | 15.09 (4.4) | 3.92 (0.14) |
| Haemoperitoneum | 3 | 21.84 (6.22–76.65) | 21.82 (48.43) | 17.92 (5.1) | 4.16 (0.16) |
| Lip oedema | 3 | 8.6 (2.64–28.06) | 8.6 (18.46) | 7.96 (2.44) | 2.99 (0.01) |
| Tearfulness | 3 | 23.66 (6.67–83.85) | 23.64 (52.04) | 19.11 (5.39) | 4.26 (0.16) |
| Ophthalmoplegia | 3 | 14.19 (4.22–47.78) | 14.19 (31.97) | 12.47 (3.7) | 3.64 (0.12) |
| Ventricular dysfunction | 3 | 12.34 (3.7–41.12) | 12.34 (27.64) | 11.03 (3.31) | 3.46 (0.09) |
| Portal vein thrombosis | 3 | 9.46 (2.89–31.01) | 9.46 (20.63) | 8.69 (2.65) | 3.12 (0.04) |
| Hepatic infarction | 3 | 94.62 (19.09–468.94) | 94.57 (138.87) | 47.78 (9.64) | 5.58 (0.09) |
| Mallory-weiss syndrome | 3 | 15.77 (4.64–53.55) | 15.76 (35.55) | 13.65 (4.02) | 3.77 (0.13) |
| Klebsiella infection | 3 | 10.51 (3.19–34.67) | 10.51 (23.23) | 9.56 (2.9) | 3.26 (0.06) |
| Oesophageal fistula | 3 | 8.6 (2.64–28.06) | 8.6 (18.46) | 7.96 (2.44) | 2.99 (0.01) |
| Systemic scleroderma | 3 | 21.84 (6.22–76.65) | 21.82 (48.43) | 17.92 (5.1) | 4.16 (0.16) |
| Ischaemic cardiomyopathy | 3 | 40.55 (10.48–156.87) | 40.53 (80.97) | 28.67 (7.41) | 4.84 (0.16) |
| Retinal artery occlusion | 3 | 21.84 (6.22–76.65) | 21.82 (48.43) | 17.92 (5.1) | 4.16 (0.16) |
| Cerebellar ataxia | 3 | 15.77 (4.64–53.55) | 15.76 (35.55) | 13.65 (4.02) | 3.77 (0.13) |
| Coronary artery occlusion | 3 | 12.34 (3.7–41.12) | 12.34 (27.64) | 11.03 (3.31) | 3.46 (0.09) |
| Psychomotor skills impaired | 3 | 25.81 (7.2–92.53) | 25.79 (56.17) | 20.48 (5.71) | 4.36 (0.17) |
| Cd4 lymphocytes decreased | 3 | 56.77 (13.56–237.63) | 56.74 (102.67) | 35.84 (8.56) | 5.16 (0.14) |
| Carotid artery thrombosis | 3 | 70.97 (15.88–317.18) | 70.93 (118.19) | 40.96 (9.16) | 5.36 (0.12) |
| Drug monitoring procedure not performed | 3 | 56.77 (13.56–237.63) | 56.74 (102.67) | 35.84 (8.56) | 5.16 (0.14) |
| Intra-abdominal haemorrhage | 3 | 25.81 (7.2–92.53) | 25.79 (56.17) | 20.48 (5.71) | 4.36 (0.17) |

Supplementary **Table S10C**

**Disproportionality analysis of** cisplatin**-associated positive signals after excluding albumin-bound paclitaxel co-reported cases in the FAERS database**

| All qualifying cisplatin-associated preferred terms after excluding albumin-bound paclitaxel co-reported cases (n=5,299) | | | | | |
| --- | --- | --- | --- | --- | --- |
| PT | N | ROR (95%CI) | PRR(χ^2^) | EBGM(EBGM05) | IC(IC025) |
| Neutropenia | 144 | 4.25 (3.59–5.03) | 4.16 (332.94) | 4.02 (3.4) | 2.01 (1.73) |
| Febrile neutropenia | 106 | 3.52 (2.9–4.29) | 3.47 (180.9) | 3.38 (2.78) | 1.76 (1.44) |
| Acute kidney injury | 98 | 3.81 (3.1–4.67) | 3.75 (191.05) | 3.64 (2.97) | 1.87 (1.53) |
| Thrombocytopenia | 83 | 2.63 (2.11–3.27) | 2.6 (79.9) | 2.55 (2.05) | 1.35 (1) |
| Pancytopenia | 73 | 3.91 (3.09–4.95) | 3.87 (149.55) | 3.75 (2.96) | 1.91 (1.51) |
| Pulmonary embolism | 65 | 3.09 (2.41–3.96) | 3.06 (87.59) | 2.99 (2.33) | 1.58 (1.17) |
| Leukopenia | 63 | 4.19 (3.25–5.41) | 4.15 (144.72) | 4.02 (3.12) | 2.01 (1.57) |
| Renal failure | 46 | 2.99 (2.23–4.02) | 2.98 (58.66) | 2.91 (2.17) | 1.54 (1.05) |
| Septic shock | 38 | 4.25 (3.07–5.89) | 4.23 (89.6) | 4.08 (2.95) | 2.03 (1.44) |
| Neutropenic sepsis | 33 | 7.2 (5.05–10.28) | 7.17 (162.38) | 6.71 (4.71) | 2.75 (2.01) |
| Toxicity to various agents | 30 | 3.63 (2.52–5.23) | 3.61 (54.59) | 3.51 (2.44) | 1.81 (1.17) |
| Oesophagitis | 29 | 5.2 (3.57–7.57) | 5.18 (92.61) | 4.95 (3.4) | 2.31 (1.58) |
| Aortic thrombosis | 29 | 75.43 (46.08–123.49) | 75.02 (1158.49) | 41.48 (25.34) | 5.37 (3.49) |
| Nephropathy toxic | 25 | 8.55 (5.67–12.89) | 8.51 (151.54) | 7.86 (5.21) | 2.98 (2.04) |
| Acute myocardial infarction | 24 | 7.06 (4.66–10.7) | 7.03 (115.33) | 6.6 (4.35) | 2.72 (1.83) |
| Bone marrow failure | 23 | 3.27 (2.15–4.96) | 3.26 (34.81) | 3.18 (2.1) | 1.67 (0.94) |
| Multiple organ dysfunction syndrome | 20 | 3.98 (2.54–6.24) | 3.97 (42.64) | 3.85 (2.46) | 1.94 (1.11) |
| Renal tubular necrosis | 19 | 9.48 (5.91–15.22) | 9.45 (130.09) | 8.65 (5.39) | 3.11 (1.96) |
| Hypomagnesaemia | 17 | 3.89 (2.39–6.32) | 3.88 (34.84) | 3.76 (2.31) | 1.91 (1.01) |
| Renal salt-wasting syndrome | 16 | 242.19 (94.73–619.17) | 241.46 (1044.97) | 66.58 (26.04) | 6.06 (2.84) |
| Gastrointestinal toxicity | 16 | 6.82 (4.1–11.34) | 6.8 (73.68) | 6.4 (3.85) | 2.68 (1.55) |
| Tinnitus | 15 | 6.39 (3.78–10.8) | 6.38 (63.56) | 6.02 (3.57) | 2.59 (1.44) |
| Cytomegalovirus oesophagitis | 15 | 170.25 (72.15–401.74) | 169.78 (875.42) | 59.7 (25.3) | 5.9 (2.73) |
| Inappropriate antidiuretic hormone secretion | 13 | 4.87 (2.79–8.52) | 4.86 (37.89) | 4.67 (2.67) | 2.22 (1.09) |
| Posterior reversible encephalopathy syndrome | 13 | 6.24 (3.56–10.95) | 6.23 (53.4) | 5.89 (3.36) | 2.56 (1.32) |
| Orthostatic hypotension | 12 | 8.19 (4.53–14.79) | 8.17 (69.28) | 7.58 (4.19) | 2.92 (1.49) |
| Aphasia | 11 | 3.91 (2.14–7.16) | 3.91 (22.81) | 3.79 (2.07) | 1.92 (0.76) |
| Hypoacusis | 11 | 3.88 (2.12–7.1) | 3.88 (22.52) | 3.76 (2.05) | 1.91 (0.75) |
| Renal infarct | 11 | 35.64 (17.74–71.63) | 35.57 (265.38) | 25.82 (12.85) | 4.69 (2.11) |
| Deafness | 10 | 3.96 (2.1–7.46) | 3.95 (21.16) | 3.83 (2.03) | 1.94 (0.71) |
| Arterial thrombosis | 10 | 19.3 (9.75–38.21) | 19.27 (142.81) | 16.06 (8.11) | 4.01 (1.8) |
| Capillary leak syndrome | 10 | 14.17 (7.27–27.61) | 14.15 (105.69) | 12.37 (6.35) | 3.63 (1.66) |
| Peripheral artery thrombosis | 10 | 25.2 (12.5–50.8) | 25.15 (181.52) | 19.9 (9.87) | 4.31 (1.89) |
| Febrile bone marrow aplasia | 10 | 4.36 (2.31–8.23) | 4.35 (24.66) | 4.2 (2.23) | 2.07 (0.8) |
| Cyanosis | 9 | 4.89 (2.5–9.56) | 4.88 (26.35) | 4.68 (2.39) | 2.23 (0.83) |
| Neurotoxicity | 9 | 4.51 (2.31–8.81) | 4.5 (23.37) | 4.34 (2.22) | 2.12 (0.76) |
| Presyncope | 9 | 9.27 (4.67–18.43) | 9.26 (60.18) | 8.49 (4.28) | 3.09 (1.31) |
| Enteritis | 9 | 4.41 (2.26–8.62) | 4.4 (22.6) | 4.25 (2.17) | 2.09 (0.74) |
| Melanoderma | 9 | 74.21 (30.74–179.15) | 74.08 (356.9) | 41.2 (17.06) | 5.36 (1.91) |
| Bicytopenia | 9 | 9.38 (4.72–18.64) | 9.37 (60.97) | 8.58 (4.32) | 3.1 (1.32) |
| Peripheral ischaemia | 9 | 7.04 (3.57–13.87) | 7.03 (43.17) | 6.59 (3.34) | 2.72 (1.12) |
| Arterial occlusive disease | 9 | 27.21 (12.91–57.34) | 27.16 (174.48) | 21.13 (10.03) | 4.4 (1.78) |
| Splenic infarction | 8 | 23.4 (10.75–50.93) | 23.37 (136.15) | 18.78 (8.63) | 4.23 (1.58) |
| Cerebral venous thrombosis | 8 | 65.95 (26.52–164.03) | 65.85 (295.82) | 38.55 (15.5) | 5.27 (1.73) |
| Metabolic acidosis | 7 | 5.2 (2.43–11.15) | 5.2 (22.43) | 4.97 (2.32) | 2.31 (0.67) |
| Empyema | 6 | 8.37 (3.62–19.32) | 8.36 (35.59) | 7.74 (3.35) | 2.95 (0.83) |
| Ototoxicity | 6 | 17 (7.1–40.66) | 16.98 (75.98) | 14.45 (6.04) | 3.85 (1.11) |
| Hepatitis b | 6 | 10.88 (4.66–25.38) | 10.87 (47.99) | 9.81 (4.2) | 3.29 (0.95) |
| Hypovolaemia | 6 | 5.98 (2.61–13.66) | 5.97 (23.29) | 5.66 (2.48) | 2.5 (0.63) |
| Ileus paralytic | 6 | 6.32 (2.76–14.47) | 6.32 (25.11) | 5.97 (2.61) | 2.58 (0.66) |
| Radiation oesophagitis | 6 | 6.88 (3–15.79) | 6.88 (28.01) | 6.46 (2.82) | 2.69 (0.72) |
| Type i hypersensitivity | 6 | 20.92 (8.61–50.84) | 20.9 (92.35) | 17.17 (7.06) | 4.1 (1.16) |
| Arteriospasm coronary | 6 | 12.95 (5.5–30.47) | 12.94 (57.82) | 11.44 (4.86) | 3.52 (1.02) |
| Neutropenic colitis | 6 | 6.97 (3.04–16) | 6.97 (28.47) | 6.54 (2.85) | 2.71 (0.72) |
| Splenic rupture | 5 | 45.32 (15.48–132.62) | 45.27 (144.32) | 30.52 (10.43) | 4.93 (0.97) |
| Intestinal ischaemia | 5 | 6.47 (2.61–16.04) | 6.47 (21.57) | 6.1 (2.46) | 2.61 (0.49) |
| Pulmonary nocardiosis | 5 | 64.74 (20.54–204.04) | 64.68 (182.86) | 38.14 (12.1) | 5.25 (0.97) |
| Hypoparathyroidism | 5 | 32.37 (11.65–89.9) | 32.34 (111.89) | 24.09 (8.67) | 4.59 (0.96) |
| Dilated cardiomyopathy | 5 | 10.54 (4.17–26.61) | 10.53 (38.63) | 9.54 (3.78) | 3.25 (0.72) |
| Hypercreatininaemia | 5 | 10.07 (4–25.38) | 10.06 (36.72) | 9.15 (3.63) | 3.19 (0.7) |
| Idiopathic pulmonary fibrosis | 5 | 9.44 (3.76–23.72) | 9.43 (34.14) | 8.64 (3.44) | 3.11 (0.67) |
| Tonsillitis | 4 | 20.14 (6.81–59.52) | 20.12 (59.47) | 16.64 (5.63) | 4.06 (0.57) |
| Kaposi's sarcoma | 4 | 90.61 (22.66–362.42) | 90.55 (177.12) | 45.77 (11.44) | 5.52 (0.58) |
| Diastolic dysfunction | 4 | 17.26 (5.92–50.3) | 17.25 (51.43) | 14.65 (5.03) | 3.87 (0.55) |
| Necrosis ischaemic | 4 | 362.46 (40.5–3243.56) | 362.19 (288.15) | 73.24 (8.18) | 6.19 (0.48) |
| Skin hyperpigmentation | 4 | 6.04 (2.19–16.63) | 6.04 (15.76) | 5.72 (2.08) | 2.52 (0.21) |
| Ulcer haemorrhage | 4 | 10.07 (3.58–28.3) | 10.06 (29.38) | 9.15 (3.26) | 3.19 (0.42) |
| Recall phenomenon | 4 | 9.06 (3.24–25.33) | 9.05 (26.06) | 8.32 (2.98) | 3.06 (0.38) |
| Embolism arterial | 4 | 6.97 (2.52–19.28) | 6.97 (18.98) | 6.54 (2.36) | 2.71 (0.28) |
| Subclavian vein thrombosis | 4 | 12.5 (4.39–35.56) | 12.49 (37.16) | 11.1 (3.9) | 3.47 (0.48) |
| Coronary artery thrombosis | 4 | 25.89 (8.52–78.68) | 25.87 (74.39) | 20.34 (6.69) | 4.35 (0.6) |
| Diabetes mellitus inadequate control | 4 | 6.47 (2.35–17.85) | 6.47 (17.26) | 6.1 (2.21) | 2.61 (0.24) |
| Drug monitoring procedure incorrectly performed | 4 | 362.46 (40.5–3243.56) | 362.19 (288.15) | 73.24 (8.18) | 6.19 (0.48) |
| Contraindicated product administered | 4 | 10.98 (3.89–31.01) | 10.98 (32.35) | 9.9 (3.5) | 3.31 (0.45) |
| Fibrosis | 3 | 12.94 (3.86–43.4) | 12.94 (28.91) | 11.44 (3.41) | 3.52 (0.1) |
| Salmonellosis | 3 | 67.95 (15.2–303.68) | 67.91 (113.02) | 39.23 (8.78) | 5.29 (0.11) |
| Myopericarditis | 3 | 12.35 (3.7–41.29) | 12.35 (27.53) | 10.99 (3.29) | 3.46 (0.09) |
| Formication | 3 | 16.99 (4.95–58.31) | 16.98 (37.99) | 14.45 (4.21) | 3.85 (0.13) |
| Haemoperitoneum | 3 | 20.91 (5.96–73.39) | 20.9 (46.18) | 17.17 (4.89) | 4.1 (0.15) |
| Tearfulness | 3 | 22.65 (6.39–80.28) | 22.64 (49.64) | 18.31 (5.17) | 4.19 (0.15) |
| Ophthalmoplegia | 3 | 13.59 (4.04–45.75) | 13.58 (30.41) | 11.94 (3.55) | 3.58 (0.11) |
| Ventricular dysfunction | 3 | 11.82 (3.55–39.37) | 11.81 (26.26) | 10.56 (3.17) | 3.4 (0.08) |
| Portal vein thrombosis | 3 | 9.06 (2.76–29.69) | 9.05 (19.54) | 8.32 (2.54) | 3.06 (0.02) |
| Hepatic infarction | 3 | 90.6 (18.28–448.98) | 90.55 (132.84) | 45.77 (9.24) | 5.52 (0.08) |
| Mallory-weiss syndrome | 3 | 15.1 (4.45–51.28) | 15.09 (33.83) | 13.08 (3.85) | 3.71 (0.12) |
| Klebsiella infection | 3 | 10.07 (3.05–33.19) | 10.06 (22.03) | 9.15 (2.78) | 3.19 (0.05) |
| Systemic scleroderma | 3 | 20.91 (5.96–73.39) | 20.9 (46.18) | 17.17 (4.89) | 4.1 (0.15) |
| Ischaemic cardiomyopathy | 3 | 38.83 (10.04–150.19) | 38.81 (77.35) | 27.46 (7.1) | 4.78 (0.15) |
| Retinal artery occlusion | 3 | 20.91 (5.96–73.39) | 20.9 (46.18) | 17.17 (4.89) | 4.1 (0.15) |
| Cerebellar ataxia | 3 | 15.1 (4.45–51.28) | 15.09 (33.83) | 13.08 (3.85) | 3.71 (0.12) |
| Coronary artery occlusion | 3 | 11.82 (3.55–39.37) | 11.81 (26.26) | 10.56 (3.17) | 3.4 (0.08) |
| Psychomotor skills impaired | 3 | 24.71 (6.89–88.59) | 24.69 (53.59) | 19.62 (5.47) | 4.29 (0.16) |
| Cd4 lymphocytes decreased | 3 | 54.36 (12.99–227.52) | 54.33 (98.15) | 34.33 (8.2) | 5.1 (0.13) |
| Carotid artery thrombosis | 3 | 67.95 (15.2–303.68) | 67.91 (113.02) | 39.23 (8.78) | 5.29 (0.11) |
| Drug monitoring procedure not performed | 3 | 54.36 (12.99–227.52) | 54.33 (98.15) | 34.33 (8.2) | 5.1 (0.13) |
| Intra-abdominal haemorrhage | 3 | 24.71 (6.89–88.59) | 24.69 (53.59) | 19.62 (5.47) | 4.29 (0.16) |

Supplementary **Table S11A**

Disproportionality analysis of all qualifying preferred terms associated with carboplatin in the age subgroup of patients aged <65 years in the FAERS database

| All qualifying carboplatin-associated preferred terms in patients aged <65 years (n=11,077) | | | | | |
| --- | --- | --- | --- | --- | --- |
| PT | N | ROR (95%CI) | PRR(χ^2^) | EBGM(EBGM05) | IC(IC025) |
| Pancytopenia | 195 | 5.69 (4.8–6.73) | 5.6 (516.23) | 4.21 (3.55) | 2.07 (1.81) |
| Leukopenia | 129 | 4.7 (3.84–5.76) | 4.66 (273.4) | 3.69 (3.01) | 1.88 (1.57) |
| Mucosal inflammation | 62 | 3.14 (2.37–4.14) | 3.12 (72.24) | 2.71 (2.05) | 1.44 (1) |
| Therapy partial responder | 60 | 4.42 (3.29–5.92) | 4.4 (117.65) | 3.53 (2.63) | 1.82 (1.34) |
| Septic shock | 58 | 3.43 (2.57–4.58) | 3.42 (78.52) | 2.91 (2.18) | 1.54 (1.08) |
| Hepatic cytolysis | 56 | 3.98 (2.95–5.38) | 3.97 (95.27) | 3.27 (2.42) | 1.71 (1.22) |
| Therapeutic product effect incomplete | 44 | 6.55 (4.55–9.41) | 6.52 (136.81) | 4.67 (3.25) | 2.22 (1.6) |
| Anaphylactic shock | 43 | 5.56 (3.89–7.96) | 5.55 (112.19) | 4.18 (2.92) | 2.06 (1.46) |
| Neutropenic sepsis | 34 | 4.83 (3.26–7.17) | 4.82 (75) | 3.78 (2.55) | 1.92 (1.25) |
| Polyneuropathy | 31 | 7.03 (4.54–10.9) | 7.02 (103.64) | 4.9 (3.16) | 2.29 (1.52) |
| Therapy non-responder | 28 | 4.96 (3.21–7.67) | 4.95 (63.8) | 3.85 (2.49) | 1.95 (1.19) |
| Posterior reversible encephalopathy syndrome | 28 | 3.93 (2.58–6.01) | 3.93 (46.85) | 3.24 (2.12) | 1.7 (0.98) |
| Febrile bone marrow aplasia | 22 | 4.31 (2.66–6.98) | 4.3 (41.81) | 3.48 (2.14) | 1.8 (0.96) |
| Therapeutic response decreased | 20 | 5.62 (3.32–9.5) | 5.61 (52.81) | 4.21 (2.49) | 2.07 (1.13) |
| Immune-mediated hypothyroidism | 18 | 6.12 (3.49–10.72) | 6.11 (52.23) | 4.47 (2.55) | 2.16 (1.13) |
| Restlessness | 16 | 6.26 (3.45–11.38) | 6.25 (47.59) | 4.54 (2.5) | 2.18 (1.08) |
| Angioedema | 15 | 4.72 (2.61–8.54) | 4.72 (32.21) | 3.72 (2.06) | 1.9 (0.83) |
| Myelodysplastic syndrome | 14 | 7.86 (4.04–15.28) | 7.85 (52.05) | 5.26 (2.71) | 2.39 (1.13) |
| Congenital aplasia | 14 | 8.61 (4.38–16.94) | 8.6 (56.43) | 5.56 (2.83) | 2.48 (1.18) |
| Renal tubular disorder | 14 | 7.53 (3.9–14.57) | 7.53 (50.04) | 5.12 (2.65) | 2.36 (1.1) |
| Autoimmune haemolytic anaemia | 14 | 10.64 (5.24–21.58) | 10.62 (66.95) | 6.28 (3.09) | 2.65 (1.28) |
| Bicytopenia | 13 | 7.3 (3.7–14.41) | 7.29 (45.1) | 5.02 (2.54) | 2.33 (1.03) |
| Neutropenic colitis | 13 | 7.63 (3.84–15.15) | 7.62 (47.03) | 5.16 (2.6) | 2.37 (1.05) |
| Endocarditis | 12 | 9.12 (4.35–19.09) | 9.11 (50.77) | 5.75 (2.75) | 2.52 (1.08) |
| Lip oedema | 11 | 14.2 (6.03–33.45) | 14.19 (64.23) | 7.28 (3.09) | 2.86 (1.18) |
| Jugular vein thrombosis | 11 | 6.17 (3.01–12.67) | 6.17 (32.24) | 4.5 (2.19) | 2.17 (0.8) |
| Rash vesicular | 10 | 8.61 (3.87–19.16) | 8.6 (40.31) | 5.56 (2.5) | 2.48 (0.9) |
| Raynaud's phenomenon | 10 | 7.17 (3.31–15.54) | 7.17 (34.12) | 4.96 (2.29) | 2.31 (0.81) |
| Faecaloma | 9 | 8.3 (3.59–19.18) | 8.29 (35.14) | 5.44 (2.35) | 2.44 (0.79) |
| Contraindicated product administered | 9 | 9.68 (4.08–22.98) | 9.68 (40.01) | 5.96 (2.51) | 2.57 (0.85) |
| Herpes simplex reactivation | 8 | 17.21 (5.97–49.62) | 17.2 (52.32) | 7.94 (2.76) | 2.99 (0.9) |
| Cutaneous lupus erythematosus | 8 | 10.33 (4.08–26.17) | 10.32 (37.41) | 6.18 (2.44) | 2.63 (0.76) |
| Exposure during pregnancy | 7 | 11.29 (4.09–31.15) | 11.29 (35.01) | 6.49 (2.35) | 2.7 (0.65) |
| Fanconi syndrome | 6 | 12.91 (4.16–40.03) | 12.9 (32.94) | 6.95 (2.24) | 2.8 (0.5) |
| Hyperphosphaturia | 6 | 19.36 (5.46–68.62) | 19.35 (41.77) | 8.34 (2.35) | 3.06 (0.57) |
| Antinuclear antibody | 6 | 25.81 (6.46–103.23) | 25.8 (47.68) | 9.27 (2.32) | 3.21 (0.6) |
| Aplasia pure red cell | 6 | 9.68 (3.36–27.9) | 9.68 (26.67) | 5.96 (2.07) | 2.57 (0.43) |
| Optic ischaemic neuropathy | 6 | 38.72 (7.81–191.88) | 38.7 (55.09) | 10.43 (2.1) | 3.38 (0.63) |
| Magnetic resonance imaging abnormal | 6 | 12.91 (4.16–40.03) | 12.9 (32.94) | 6.95 (2.24) | 2.8 (0.5) |
| Immune-mediated hyperthyroidism | 6 | 11.06 (3.72–32.92) | 11.06 (29.56) | 6.42 (2.16) | 2.68 (0.47) |
| Blood phosphorus abnormal | 6 | 19.36 (5.46–68.62) | 19.35 (41.77) | 8.34 (2.35) | 3.06 (0.57) |
| Stenosis | 5 | 12.91 (3.74–44.59) | 12.9 (27.45) | 6.95 (2.01) | 2.8 (0.28) |
| Paraneoplastic rash | 5 | 21.51 (5.14–90.02) | 21.5 (36.65) | 8.69 (2.08) | 3.12 (0.34) |
| Normocytic anaemia | 5 | 16.13 (4.33–60.09) | 16.13 (31.53) | 7.72 (2.07) | 2.95 (0.31) |

Supplementary **Table S11B**

Disproportionality analysis of all qualifying preferred terms associated with carboplatin in the age subgroup of patients aged ≥65 years in the FAERS database

| All qualifying carboplatin-associated preferred terms in patients aged ≥65 years (n=15,567) | | | | | |
| --- | --- | --- | --- | --- | --- |
| PT | N | ROR (95%CI) | PRR(χ^2^) | EBGM(EBGM05) | IC(IC025) |
| Thrombocytopenia | 304 | 3.65 (3.21–4.15) | 3.6 (444.12) | 3.01 (2.65) | 1.59 (1.4) |
| Pancytopenia | 298 | 5.83 (5.08–6.7) | 5.74 (799.07) | 4.23 (3.69) | 2.08 (1.87) |
| Neutropenia | 296 | 3.35 (2.94–3.81) | 3.31 (377.85) | 2.82 (2.48) | 1.49 (1.3) |
| Leukopenia | 172 | 5.05 (4.23–6.03) | 5 (392.98) | 3.85 (3.22) | 1.94 (1.67) |
| Septic shock | 96 | 3.24 (2.59–4.06) | 3.23 (117.24) | 2.77 (2.21) | 1.47 (1.12) |
| Mucosal inflammation | 81 | 3 (2.35–3.82) | 2.98 (86.22) | 2.6 (2.04) | 1.38 (1) |
| Neutropenic sepsis | 79 | 5.92 (4.52–7.74) | 5.89 (217.29) | 4.31 (3.29) | 2.11 (1.67) |
| Polyneuropathy | 67 | 8.36 (6.13–11.41) | 8.33 (257.93) | 5.37 (3.94) | 2.43 (1.91) |
| Therapy partial responder | 57 | 3.21 (2.4–4.3) | 3.2 (68.64) | 2.75 (2.05) | 1.46 (0.99) |
| Agranulocytosis | 56 | 4.67 (3.43–6.35) | 4.66 (116.79) | 3.65 (2.68) | 1.87 (1.36) |
| Hypomagnesaemia | 51 | 3.84 (2.8–5.26) | 3.83 (81.34) | 3.16 (2.3) | 1.66 (1.15) |
| Rash maculo-papular | 49 | 3.93 (2.85–5.42) | 3.92 (80.8) | 3.21 (2.33) | 1.68 (1.16) |
| Febrile bone marrow aplasia | 44 | 7.87 (5.39–11.49) | 7.85 (160.65) | 5.18 (3.55) | 2.37 (1.73) |
| Hypertransaminasaemia | 40 | 6.49 (4.43–9.52) | 6.48 (121.47) | 4.59 (3.13) | 2.2 (1.54) |
| Hepatocellular injury | 31 | 4.39 (2.91–6.62) | 4.39 (59.77) | 3.5 (2.32) | 1.81 (1.11) |
| Toxic skin eruption | 30 | 4.02 (2.66–6.07) | 4.01 (51.22) | 3.27 (2.17) | 1.71 (1.02) |
| Therapy non-responder | 25 | 3.95 (2.52–6.2) | 3.94 (41.64) | 3.23 (2.06) | 1.69 (0.93) |
| Immune-mediated hypothyroidism | 24 | 4.93 (3.07–7.92) | 4.92 (53.62) | 3.8 (2.37) | 1.93 (1.1) |
| Acute myeloid leukaemia | 22 | 4.3 (2.65–6.99) | 4.3 (41.28) | 3.44 (2.12) | 1.78 (0.95) |
| Lymphopenia | 21 | 4.54 (2.75–7.49) | 4.53 (42.3) | 3.58 (2.17) | 1.84 (0.97) |
| Encephalitis autoimmune | 21 | 4.79 (2.89–7.93) | 4.79 (45.31) | 3.73 (2.25) | 1.9 (1.01) |
| Clostridium difficile colitis | 21 | 4.04 (2.47–6.62) | 4.04 (36.16) | 3.29 (2.01) | 1.72 (0.87) |
| Prescribed overdose | 20 | 4.65 (2.78–7.78) | 4.64 (41.54) | 3.65 (2.18) | 1.87 (0.96) |
| Dermatitis bullous | 20 | 5.13 (3.05–8.65) | 5.13 (46.93) | 3.91 (2.32) | 1.97 (1.04) |
| Bicytopenia | 19 | 6.89 (3.93–12.07) | 6.88 (61.25) | 4.77 (2.72) | 2.25 (1.23) |
| Dermatitis exfoliative generalised | 18 | 6.34 (3.59–11.19) | 6.33 (53.36) | 4.52 (2.56) | 2.18 (1.14) |
| Posterior reversible encephalopathy syndrome | 17 | 5.66 (3.19–10.05) | 5.66 (44.65) | 4.19 (2.36) | 2.07 (1.03) |
| Immune-mediated hyperthyroidism | 17 | 14.96 (7.37–30.36) | 14.95 (99.92) | 7.3 (3.6) | 2.87 (1.55) |
| Congenital aplasia | 16 | 11.6 (5.86–22.95) | 11.58 (79.72) | 6.45 (3.26) | 2.69 (1.4) |
| Extremity necrosis | 13 | 5.34 (2.78–10.23) | 5.33 (31.94) | 4.02 (2.1) | 2.01 (0.82) |
| Myelitis transverse | 12 | 9.85 (4.61–21.05) | 9.85 (52.99) | 5.91 (2.77) | 2.56 (1.1) |
| Meningitis aseptic | 12 | 6.43 (3.2–12.92) | 6.42 (36.1) | 4.56 (2.27) | 2.19 (0.88) |
| Peripheral sensorimotor neuropathy | 11 | 9.03 (4.15–19.67) | 9.03 (45.3) | 5.63 (2.59) | 2.49 (0.98) |
| Troponin t increased | 10 | 8.8 (3.91–19.81) | 8.79 (40.29) | 5.55 (2.46) | 2.47 (0.89) |
| Jaundice cholestatic | 10 | 6.16 (2.88–13.16) | 6.15 (28.78) | 4.44 (2.08) | 2.15 (0.71) |
| Muscle fatigue | 9 | 9.24 (3.89–21.92) | 9.23 (37.75) | 5.7 (2.4) | 2.51 (0.81) |
| Endocarditis | 8 | 7.04 (2.95–16.78) | 7.03 (26.35) | 4.84 (2.03) | 2.27 (0.59) |
| Pancreatic failure | 8 | 9.85 (3.89–24.96) | 9.85 (35.33) | 5.91 (2.33) | 2.56 (0.72) |
| Systemic lupus erythematosus | 8 | 7.58 (3.14–18.29) | 7.57 (28.26) | 5.07 (2.1) | 2.34 (0.62) |
| Autoimmune hypothyroidism | 8 | 7.58 (3.14–18.29) | 7.57 (28.26) | 5.07 (2.1) | 2.34 (0.62) |
| Clonus | 7 | 12.31 (4.32–35.11) | 12.31 (36.37) | 6.65 (2.33) | 2.73 (0.65) |
| Oesophageal disorder | 7 | 9.58 (3.57–25.72) | 9.57 (30.23) | 5.82 (2.17) | 2.54 (0.58) |
| Oesophagitis ulcerative | 7 | 10.77 (3.91–29.72) | 10.77 (33.09) | 6.21 (2.25) | 2.63 (0.61) |
| Febrile infection | 7 | 9.58 (3.57–25.72) | 9.57 (30.23) | 5.82 (2.17) | 2.54 (0.58) |
| Intestinal pseudo-obstruction | 7 | 9.58 (3.57–25.72) | 9.57 (30.23) | 5.82 (2.17) | 2.54 (0.58) |
| Glucocorticoid deficiency | 7 | 9.58 (3.57–25.72) | 9.57 (30.23) | 5.82 (2.17) | 2.54 (0.58) |
| Large intestine infection | 7 | 7.84 (3.04–20.22) | 7.83 (25.5) | 5.18 (2.01) | 2.37 (0.5) |
| Shock symptom | 6 | 18.47 (5.21–65.46) | 18.46 (39.64) | 7.98 (2.25) | 3 (0.54) |
| Infusion site erythema | 6 | 18.47 (5.21–65.46) | 18.46 (39.64) | 7.98 (2.25) | 3 (0.54) |
| Blood creatine phosphokinase abnormal | 6 | 14.78 (4.51–48.42) | 14.77 (35.01) | 7.26 (2.22) | 2.86 (0.51) |
| Infusion related hypersensitivity reaction | 6 | 18.47 (5.21–65.46) | 18.46 (39.64) | 7.98 (2.25) | 3 (0.54) |
| Immune-mediated encephalopathy | 6 | 10.55 (3.55–31.41) | 10.55 (27.93) | 6.14 (2.06) | 2.62 (0.43) |

Supplementary **Table S11C**

Disproportionality analysis of all qualifying preferred terms associated with carboplatin in the sex subgroup of male patients in the FAERS database

| All qualifying carboplatin-associated preferred terms in male patients (n=16,611) | | | | | |
| --- | --- | --- | --- | --- | --- |
| PT | N | ROR (95%CI) | PRR(χ^2^) | EBGM(EBGM05) | IC(IC025) |
| Pancytopenia | 334 | 5.77 (5.07–6.58) | 5.68 (892.1) | 4.23 (3.71) | 2.08 (1.88) |
| Neutropenia | 320 | 2.95 (2.61–3.33) | 2.91 (328.41) | 2.55 (2.26) | 1.35 (1.17) |
| Thrombocytopenia | 313 | 3.08 (2.72–3.49) | 3.04 (347.96) | 2.64 (2.34) | 1.4 (1.22) |
| Leukopenia | 214 | 5.15 (4.39–6.04) | 5.1 (504.02) | 3.92 (3.34) | 1.97 (1.73) |
| Septic shock | 121 | 3.3 (2.7–4.03) | 3.28 (152.56) | 2.81 (2.3) | 1.49 (1.18) |
| Mucosal inflammation | 94 | 3.45 (2.75–4.34) | 3.44 (128.16) | 2.92 (2.32) | 1.55 (1.19) |
| Therapy partial responder | 73 | 3.94 (3.03–5.12) | 3.92 (121.47) | 3.23 (2.48) | 1.69 (1.27) |
| Neutropenic sepsis | 72 | 5.46 (4.14–7.21) | 5.44 (182.74) | 4.11 (3.11) | 2.04 (1.59) |
| Polyneuropathy | 70 | 9.24 (6.79–12.58) | 9.21 (296.43) | 5.75 (4.22) | 2.52 (2.01) |
| Agranulocytosis | 60 | 4.84 (3.59–6.52) | 4.83 (131.81) | 3.77 (2.8) | 1.91 (1.43) |
| Hepatic cytolysis | 52 | 3.45 (2.54–4.68) | 3.44 (70.75) | 2.92 (2.15) | 1.54 (1.05) |
| Rash maculo-papular | 50 | 3.56 (2.6–4.87) | 3.55 (71.48) | 2.99 (2.18) | 1.58 (1.07) |
| Therapy non-responder | 41 | 5.4 (3.75–7.79) | 5.39 (102.89) | 4.08 (2.83) | 2.03 (1.41) |
| Febrile bone marrow aplasia | 41 | 5.04 (3.51–7.24) | 5.03 (94.67) | 3.88 (2.7) | 1.96 (1.35) |
| Hypomagnesaemia | 39 | 3.43 (2.4–4.88) | 3.42 (52.6) | 2.9 (2.04) | 1.54 (0.96) |
| Hepatocellular injury | 37 | 4.22 (2.91–6.12) | 4.21 (67.95) | 3.41 (2.35) | 1.77 (1.15) |
| Anaphylactic shock | 36 | 3.56 (2.46–5.15) | 3.55 (51.55) | 2.99 (2.07) | 1.58 (0.97) |
| Hypertransaminasaemia | 35 | 4.47 (3.04–6.58) | 4.46 (69.57) | 3.56 (2.42) | 1.83 (1.18) |
| Toxic epidermal necrolysis | 35 | 4.52 (3.07–6.65) | 4.51 (70.5) | 3.59 (2.44) | 1.84 (1.19) |
| Therapeutic product effect incomplete | 33 | 4.54 (3.05–6.75) | 4.53 (66.85) | 3.6 (2.42) | 1.85 (1.17) |
| Acute myeloid leukaemia | 27 | 4.68 (3.01–7.27) | 4.67 (56.89) | 3.68 (2.37) | 1.88 (1.12) |
| Immune-mediated hypothyroidism | 26 | 4.83 (3.08–7.6) | 4.83 (57.12) | 3.77 (2.4) | 1.91 (1.13) |
| Bicytopenia | 24 | 7.59 (4.57–12.59) | 7.58 (85.66) | 5.11 (3.08) | 2.35 (1.44) |
| Therapeutic response decreased | 21 | 4.08 (2.5–6.68) | 4.08 (36.92) | 3.33 (2.03) | 1.73 (0.89) |
| Congenital aplasia | 20 | 10.11 (5.62–18.21) | 10.1 (91.15) | 6.06 (3.36) | 2.6 (1.5) |
| Encephalitis autoimmune | 18 | 4.29 (2.51–7.33) | 4.29 (33.9) | 3.46 (2.02) | 1.79 (0.85) |
| Posterior reversible encephalopathy syndrome | 17 | 4.67 (2.68–8.15) | 4.67 (35.78) | 3.68 (2.11) | 1.88 (0.89) |
| Dermatitis exfoliative generalised | 17 | 8.6 (4.64–15.92) | 8.59 (67.85) | 5.52 (2.98) | 2.46 (1.3) |
| Aphthous ulcer | 16 | 7.22 (3.91–13.35) | 7.22 (54.54) | 4.96 (2.68) | 2.31 (1.16) |
| Acute generalised exanthematous pustulosis | 16 | 5.62 (3.12–10.12) | 5.61 (42) | 4.19 (2.33) | 2.07 (0.99) |
| Immune-mediated hyperthyroidism | 14 | 14.75 (6.82–31.89) | 14.73 (82.73) | 7.34 (3.39) | 2.88 (1.4) |
| Faecaloma | 11 | 6.62 (3.19–13.73) | 6.62 (34.41) | 4.68 (2.26) | 2.23 (0.84) |
| Peripheral sensorimotor neuropathy | 11 | 8.18 (3.83–17.46) | 8.17 (42.04) | 5.35 (2.51) | 2.42 (0.95) |
| Demyelinating polyneuropathy | 11 | 9.27 (4.26–20.18) | 9.26 (46.77) | 5.77 (2.65) | 2.53 (1.01) |
| Lip oedema | 10 | 25.27 (8.64–73.95) | 25.26 (77.66) | 9.09 (3.11) | 3.18 (1.21) |
| Progressive multifocal leukoencephalopathy | 10 | 12.64 (5.26–30.36) | 12.63 (53.55) | 6.81 (2.84) | 2.77 (1.04) |
| Jugular vein thrombosis | 9 | 8.12 (3.52–18.77) | 8.12 (34.2) | 5.33 (2.31) | 2.41 (0.77) |
| Necrotising myositis | 9 | 8.75 (3.74–20.47) | 8.74 (36.47) | 5.58 (2.38) | 2.48 (0.8) |
| Contraindicated product administered | 9 | 10.34 (4.28–24.95) | 10.33 (41.73) | 6.13 (2.54) | 2.62 (0.87) |
| Magnetic resonance imaging abnormal | 9 | 14.21 (5.48–36.85) | 14.21 (52) | 7.22 (2.78) | 2.85 (0.97) |
| Sarcoidosis | 8 | 7.22 (3.03–17.21) | 7.22 (27.26) | 4.96 (2.08) | 2.31 (0.61) |
| Acute promyelocytic leukaemia | 8 | 7.22 (3.03–17.21) | 7.22 (27.26) | 4.96 (2.08) | 2.31 (0.61) |
| Intestinal pseudo-obstruction | 8 | 9.19 (3.7–22.85) | 9.18 (33.78) | 5.74 (2.31) | 2.52 (0.71) |
| Cutaneous lupus erythematosus | 8 | 12.63 (4.74–33.67) | 12.63 (42.84) | 6.81 (2.56) | 2.77 (0.81) |
| Herpes simplex reactivation | 8 | 33.69 (8.94–127.02) | 33.68 (69.18) | 9.91 (2.63) | 3.31 (0.99) |
| Clonus | 7 | 17.69 (5.61–55.74) | 17.68 (45.9) | 7.95 (2.52) | 2.99 (0.74) |
| Oesophagitis ulcerative | 7 | 12.63 (4.43–36.02) | 12.63 (37.48) | 6.81 (2.39) | 2.77 (0.67) |
| Large intestine infection | 7 | 11.05 (4.01–30.49) | 11.05 (34.13) | 6.36 (2.31) | 2.67 (0.63) |
| Vitritis | 6 | 37.9 (7.65–187.8) | 37.89 (53.87) | 10.22 (2.06) | 3.35 (0.62) |
| Cytomegalovirus colitis | 6 | 10.83 (3.64–32.22) | 10.82 (28.81) | 6.29 (2.11) | 2.65 (0.45) |
| Blood creatine phosphokinase abnormal | 6 | 12.63 (4.07–39.18) | 12.63 (32.13) | 6.81 (2.2) | 2.77 (0.49) |
| Anaemia of malignant disease | 6 | 12.63 (4.07–39.18) | 12.63 (32.13) | 6.81 (2.2) | 2.77 (0.49) |
| Acute interstitial pneumonitis | 6 | 15.16 (4.63–49.68) | 15.15 (36.06) | 7.43 (2.27) | 2.89 (0.52) |
| Idiopathic intracranial hypertension | 6 | 25.27 (6.32–101.04) | 25.26 (46.6) | 9.09 (2.27) | 3.18 (0.59) |
| Immune-mediated encephalopathy | 6 | 10.83 (3.64–32.22) | 10.82 (28.81) | 6.29 (2.11) | 2.65 (0.45) |
| Fournier's gangrene | 5 | 15.79 (4.24–58.81) | 15.79 (30.78) | 7.57 (2.03) | 2.92 (0.3) |
| Paraneoplastic rash | 5 | 21.05 (5.03–88.11) | 21.05 (35.81) | 8.52 (2.04) | 3.09 (0.33) |

Supplementary **Table S11D**

Disproportionality analysis of all qualifying preferred terms associated with carboplatin in the sex subgroup of female patients in the FAERS database

| All qualifying carboplatin-associated preferred terms in female patients (n=11,256) | | | | | |
| --- | --- | --- | --- | --- | --- |
| PT | N | ROR (95%CI) | PRR(χ^2^) | EBGM(EBGM05) | IC(IC025) |
| Anaemia | 258 | 3.2 (2.8–3.65) | 3.15 (324.96) | 2.83 (2.48) | 1.5 (1.3) |
| Pancytopenia | 174 | 7.18 (6.02–8.56) | 7.08 (657.92) | 5.39 (4.52) | 2.43 (2.14) |
| Neutropenia | 163 | 3.61 (3.05–4.27) | 3.57 (253.8) | 3.15 (2.66) | 1.66 (1.39) |
| Thrombocytopenia | 150 | 3.64 (3.05–4.34) | 3.6 (236.5) | 3.17 (2.66) | 1.67 (1.39) |
| Febrile neutropenia | 145 | 3.93 (3.28–4.71) | 3.89 (258.11) | 3.39 (2.83) | 1.76 (1.47) |
| Acute kidney injury | 129 | 3.2 (2.65–3.86) | 3.17 (164.14) | 2.85 (2.36) | 1.51 (1.22) |
| Leukopenia | 98 | 5.56 (4.43–6.97) | 5.52 (279.39) | 4.47 (3.57) | 2.16 (1.79) |
| Hypokalaemia | 74 | 3.05 (2.38–3.9) | 3.03 (86.74) | 2.74 (2.14) | 1.46 (1.06) |
| Hypothyroidism | 68 | 3.25 (2.51–4.21) | 3.23 (89.44) | 2.9 (2.24) | 1.54 (1.12) |
| Colitis | 65 | 3.09 (2.37–4.02) | 3.08 (78.16) | 2.78 (2.13) | 1.47 (1.05) |
| Condition aggravated | 60 | 3.38 (2.57–4.46) | 3.37 (84.6) | 3 (2.28) | 1.59 (1.14) |
| Mucosal inflammation | 57 | 2.93 (2.22–3.88) | 2.92 (62.34) | 2.66 (2.01) | 1.41 (0.96) |
| Neutropenic sepsis | 49 | 9.61 (6.8–13.58) | 9.57 (247.39) | 6.63 (4.69) | 2.73 (2.09) |
| Hepatitis | 48 | 3.56 (2.62–4.86) | 3.55 (73.9) | 3.14 (2.3) | 1.65 (1.14) |
| Therapy partial responder | 46 | 4.56 (3.3–6.29) | 4.54 (101.93) | 3.84 (2.78) | 1.94 (1.39) |
| Hyperthyroidism | 41 | 4.02 (2.86–5.63) | 4 (75.95) | 3.47 (2.47) | 1.79 (1.22) |
| Hypomagnesaemia | 39 | 4.88 (3.43–6.96) | 4.87 (94.91) | 4.06 (2.85) | 2.02 (1.4) |
| Anaphylactic shock | 39 | 6.91 (4.78–9.98) | 6.89 (142.78) | 5.28 (3.65) | 2.4 (1.73) |
| Immune-mediated enterocolitis | 39 | 7.25 (5.01–10.51) | 7.23 (150.37) | 5.47 (3.78) | 2.45 (1.77) |
| Polyneuropathy | 38 | 8.97 (6.09–13.23) | 8.94 (180.41) | 6.34 (4.3) | 2.67 (1.93) |
| Therapeutic product effect incomplete | 34 | 6.32 (4.28–9.34) | 6.31 (113.05) | 4.95 (3.35) | 2.31 (1.6) |
| Septic shock | 33 | 3.61 (2.49–5.25) | 3.61 (52.01) | 3.18 (2.19) | 1.67 (1.04) |
| Hepatic cytolysis | 33 | 3.59 (2.47–5.22) | 3.59 (51.52) | 3.16 (2.18) | 1.66 (1.03) |
| Rash maculo-papular | 32 | 3.77 (2.58–5.52) | 3.77 (54) | 3.3 (2.25) | 1.72 (1.07) |
| Toxic skin eruption | 29 | 6.59 (4.31–10.07) | 6.57 (100.97) | 5.1 (3.34) | 2.35 (1.56) |
| Posterior reversible encephalopathy syndrome | 26 | 4.93 (3.2–7.6) | 4.92 (64.1) | 4.09 (2.65) | 2.03 (1.25) |
| Hepatic failure | 25 | 3.62 (2.36–5.56) | 3.61 (39.53) | 3.18 (2.07) | 1.67 (0.93) |
| Febrile bone marrow aplasia | 25 | 11.22 (6.82–18.45) | 11.2 (144.24) | 7.33 (4.46) | 2.87 (1.88) |
| Hypertransaminasaemia | 23 | 6.51 (4.04–10.47) | 6.5 (79.04) | 5.06 (3.14) | 2.34 (1.44) |
| Agranulocytosis | 22 | 4.7 (2.94–7.52) | 4.7 (51) | 3.94 (2.47) | 1.98 (1.13) |
| Renal tubular disorder | 22 | 22.48 (12.06–41.93) | 22.44 (202.84) | 10.65 (5.71) | 3.41 (2.13) |
| Immune-mediated hepatitis | 22 | 5.62 (3.48–9.06) | 5.61 (63.87) | 4.53 (2.81) | 2.18 (1.29) |
| Renal tubular necrosis | 21 | 5.68 (3.48–9.27) | 5.67 (61.74) | 4.57 (2.8) | 2.19 (1.27) |
| Dermatitis bullous | 20 | 8.36 (4.93–14.19) | 8.35 (88.93) | 6.05 (3.57) | 2.6 (1.54) |
| Purpura | 19 | 4.26 (2.59–7.02) | 4.25 (38.42) | 3.64 (2.21) | 1.86 (0.97) |
| Restlessness | 19 | 10.28 (5.86–18.02) | 10.26 (101.9) | 6.94 (3.96) | 2.8 (1.64) |
| Autoimmune hepatitis | 18 | 6.24 (3.66–10.66) | 6.24 (59.09) | 4.91 (2.87) | 2.3 (1.26) |
| Acute myocardial infarction | 18 | 3.85 (2.31–6.4) | 3.84 (31.32) | 3.35 (2.02) | 1.74 (0.84) |
| Immune-mediated hypothyroidism | 17 | 9.47 (5.27–17.01) | 9.46 (84.89) | 6.58 (3.67) | 2.72 (1.52) |
| Disease recurrence | 15 | 5.01 (2.83–8.88) | 5.01 (37.81) | 4.15 (2.34) | 2.05 (0.98) |
| Nephritis | 14 | 5.36 (2.95–9.73) | 5.36 (38.4) | 4.37 (2.41) | 2.13 (0.99) |
| Prescribed overdose | 13 | 6.13 (3.27–11.48) | 6.12 (41.78) | 4.84 (2.58) | 2.28 (1.04) |
| Acute myeloid leukaemia | 13 | 4.98 (2.7–9.19) | 4.97 (32.48) | 4.13 (2.23) | 2.04 (0.88) |
| Hypovolaemia | 12 | 5.65 (2.96–10.8) | 5.65 (35.12) | 4.56 (2.38) | 2.19 (0.92) |
| Pyelonephritis | 12 | 4.9 (2.59–9.27) | 4.9 (29.38) | 4.08 (2.16) | 2.03 (0.82) |
| Myelodysplastic syndrome | 12 | 5.96 (3.11–11.43) | 5.95 (37.37) | 4.74 (2.47) | 2.25 (0.96) |
| Raynaud's phenomenon | 12 | 10.5 (5.17–21.35) | 10.49 (65.58) | 7.04 (3.46) | 2.82 (1.3) |
| Neutropenic colitis | 11 | 20.22 (8.58–47.61) | 20.2 (95.59) | 10.14 (4.31) | 3.34 (1.45) |
| Endocarditis | 10 | 14.14 (6.2–32.24) | 14.12 (68.93) | 8.42 (3.69) | 3.07 (1.25) |
| Congenital aplasia | 10 | 11.48 (5.21–25.31) | 11.48 (58.85) | 7.45 (3.38) | 2.9 (1.17) |
| Dermatitis exfoliative generalised | 10 | 7.07 (3.41–14.66) | 7.06 (37.59) | 5.38 (2.59) | 2.43 (0.93) |
| Autoimmune haemolytic anaemia | 10 | 7.07 (3.41–14.66) | 7.06 (37.59) | 5.38 (2.59) | 2.43 (0.93) |
| Subacute cutaneous lupus erythematosus | 10 | 5.57 (2.74–11.3) | 5.56 (28.74) | 4.5 (2.22) | 2.17 (0.78) |
| Macular oedema | 9 | 15.03 (6.23–36.29) | 15.02 (64.8) | 8.71 (3.61) | 3.12 (1.15) |
| Hepatitis acute | 9 | 5.7 (2.7–12.05) | 5.7 (26.61) | 4.59 (2.17) | 2.2 (0.71) |
| Immune-mediated nephritis | 9 | 8.27 (3.76–18.16) | 8.26 (39.63) | 6.01 (2.74) | 2.59 (0.92) |
| Immune-mediated hypophysitis | 9 | 55.13 (14.92–203.65) | 55.08 (119.48) | 14.52 (3.93) | 3.86 (1.37) |
| Immune-mediated hyperthyroidism | 9 | 18.37 (7.29–46.3) | 18.36 (73.87) | 9.68 (3.84) | 3.28 (1.21) |
| Myelitis transverse | 8 | 9.8 (4.15–23.12) | 9.79 (41.19) | 6.73 (2.85) | 2.75 (0.88) |
| Rash morbilliform | 8 | 11.31 (4.69–27.28) | 11.3 (46.49) | 7.38 (3.06) | 2.88 (0.93) |
| Autoimmune colitis | 8 | 7.35 (3.24–16.69) | 7.34 (31.32) | 5.53 (2.44) | 2.47 (0.75) |
| Escherichia sepsis | 8 | 6.68 (2.97–15.01) | 6.68 (28.32) | 5.16 (2.3) | 2.37 (0.7) |
| Immune-mediated pancreatitis | 8 | 9.19 (3.93–21.47) | 9.18 (38.88) | 6.45 (2.76) | 2.69 (0.85) |
| Bronchopulmonary aspergillosis | 8 | 6.68 (2.97–15.01) | 6.68 (28.32) | 5.16 (2.3) | 2.37 (0.7) |
| Thrombocytosis | 7 | 6.43 (2.72–15.21) | 6.43 (23.76) | 5.02 (2.12) | 2.33 (0.55) |
| Bicytopenia | 7 | 7.56 (3.14–18.24) | 7.56 (28.23) | 5.65 (2.34) | 2.5 (0.63) |
| Oesophageal disorder | 7 | 9.19 (3.71–22.76) | 9.18 (34.02) | 6.45 (2.6) | 2.69 (0.71) |
| Autoimmune nephritis | 7 | 11.69 (4.53–30.16) | 11.68 (41.8) | 7.53 (2.92) | 2.91 (0.8) |
| Acute pulmonary oedema | 7 | 6.43 (2.72–15.21) | 6.43 (23.76) | 5.02 (2.12) | 2.33 (0.55) |
| Pancreatic failure | 7 | 12.86 (4.89–33.79) | 12.85 (45.01) | 7.97 (3.03) | 2.99 (0.83) |
| Sputum discoloured | 7 | 7.56 (3.14–18.24) | 7.56 (28.23) | 5.65 (2.34) | 2.5 (0.63) |
| Escherichia infection | 7 | 6.12 (2.6–14.41) | 6.12 (22.49) | 4.84 (2.06) | 2.28 (0.53) |
| Febrile infection | 7 | 7.56 (3.14–18.24) | 7.56 (28.23) | 5.65 (2.34) | 2.5 (0.63) |
| Exposure during pregnancy | 7 | 14.29 (5.32–38.38) | 14.28 (48.63) | 8.47 (3.15) | 3.08 (0.85) |
| Squamous cell carcinoma of lung | 7 | 7.56 (3.14–18.24) | 7.56 (28.23) | 5.65 (2.34) | 2.5 (0.63) |
| Rales | 6 | 8.48 (3.22–22.31) | 8.47 (27.07) | 6.11 (2.32) | 2.61 (0.52) |
| Shock symptom | 6 | 55.11 (11.12–273.09) | 55.08 (79.65) | 14.52 (2.93) | 3.86 (0.8) |
| Pneumonia pneumococcal | 6 | 12.25 (4.36–34.41) | 12.24 (37.16) | 7.74 (2.76) | 2.95 (0.63) |
| Troponin t increased | 6 | 18.37 (5.92–56.97) | 18.36 (49.25) | 9.68 (3.12) | 3.28 (0.71) |
| Hyperphosphaturia | 6 | 27.56 (7.77–97.66) | 27.54 (61.39) | 11.62 (3.28) | 3.54 (0.76) |
| Infusion site erythema | 6 | 36.74 (9.19–146.93) | 36.72 (69.5) | 12.91 (3.23) | 3.69 (0.78) |
| Antinuclear antibody | 6 | 55.11 (11.12–273.09) | 55.08 (79.65) | 14.52 (2.93) | 3.86 (0.8) |
| Meningitis aseptic | 6 | 7.87 (3.02–20.49) | 7.87 (25.19) | 5.81 (2.23) | 2.54 (0.49) |
| Blood corticotrophin decreased | 6 | 8.48 (3.22–22.31) | 8.47 (27.07) | 6.11 (2.32) | 2.61 (0.52) |
| Immune-mediated thyroiditis | 6 | 6.89 (2.69–17.61) | 6.89 (21.95) | 5.28 (2.07) | 2.4 (0.43) |
| Optic ischaemic neuropathy | 6 | 13.78 (4.78–39.71) | 13.77 (40.61) | 8.3 (2.88) | 3.05 (0.66) |
| Superficial vein thrombosis | 6 | 12.25 (4.36–34.41) | 12.24 (37.16) | 7.74 (2.76) | 2.95 (0.63) |
| Autoimmune hypothyroidism | 6 | 22.04 (6.73–72.24) | 22.03 (54.76) | 10.56 (3.22) | 3.4 (0.74) |
| Blood lactic acid increased | 6 | 11.02 (4.01–30.33) | 11.02 (34.15) | 7.26 (2.64) | 2.86 (0.6) |
| Blood phosphorus abnormal | 6 | 18.37 (5.92–56.97) | 18.36 (49.25) | 9.68 (3.12) | 3.28 (0.71) |
| Stenosis | 5 | 13.12 (4.16–41.35) | 13.11 (32.64) | 8.07 (2.56) | 3.01 (0.42) |
| Vascular purpura | 5 | 9.18 (3.14–26.87) | 9.18 (24.3) | 6.45 (2.21) | 2.69 (0.33) |
| Pemphigus | 5 | 30.61 (7.32–128.12) | 30.6 (53.69) | 12.1 (2.89) | 3.6 (0.51) |
| Terminal agitation | 5 | 45.92 (8.91–236.73) | 45.9 (62.75) | 13.83 (2.68) | 3.79 (0.53) |
| Punctate keratitis | 5 | 30.61 (7.32–128.12) | 30.6 (53.69) | 12.1 (2.89) | 3.6 (0.51) |
| Type i hypersensitivity | 5 | 8.35 (2.9–24.03) | 8.35 (22.23) | 6.05 (2.1) | 2.6 (0.31) |
| Blindness transient | 5 | 8.35 (2.9–24.03) | 8.35 (22.23) | 6.05 (2.1) | 2.6 (0.31) |
| Tumour hyperprogression | 5 | 11.48 (3.75–35.1) | 11.48 (29.43) | 7.45 (2.44) | 2.9 (0.39) |
| Cholestatic liver injury | 5 | 13.12 (4.16–41.35) | 13.11 (32.64) | 8.07 (2.56) | 3.01 (0.42) |
| Escherichia test positive | 5 | 15.31 (4.67–50.16) | 15.3 (36.45) | 8.8 (2.69) | 3.14 (0.45) |
| Tracheo-oesophageal fistula | 5 | 11.48 (3.75–35.1) | 11.48 (29.43) | 7.45 (2.44) | 2.9 (0.39) |
| Immune-mediated arthritis | 5 | 8.35 (2.9–24.03) | 8.35 (22.23) | 6.05 (2.1) | 2.6 (0.31) |
| Wound necrosis | 4 | 24.49 (5.48–109.44) | 24.48 (38.61) | 11.06 (2.48) | 3.47 (0.18) |
| Scleroedema | 4 | 12.24 (3.45–43.4) | 12.24 (24.77) | 7.74 (2.19) | 2.95 (0.12) |
| Fungaemia | 4 | 10.5 (3.07–35.86) | 10.49 (21.86) | 7.04 (2.06) | 2.82 (0.09) |
| Hyperplasia | 4 | 12.24 (3.45–43.4) | 12.24 (24.77) | 7.74 (2.19) | 2.95 (0.12) |
| Abdominal mass | 4 | 12.24 (3.45–43.4) | 12.24 (24.77) | 7.74 (2.19) | 2.95 (0.12) |
| Oligoastrocytoma | 4 | 24.49 (5.48–109.44) | 24.48 (38.61) | 11.06 (2.48) | 3.47 (0.18) |
| Vestibular disorder | 4 | 14.69 (3.95–54.73) | 14.69 (28.35) | 8.6 (2.31) | 3.11 (0.14) |
| Escherichia bacteraemia | 4 | 12.24 (3.45–43.4) | 12.24 (24.77) | 7.74 (2.19) | 2.95 (0.12) |
| Fusarium infection | 4 | 36.73 (6.73–200.58) | 36.72 (46.33) | 12.91 (2.36) | 3.69 (0.19) |
| Spinal cord infarction | 4 | 12.24 (3.45–43.4) | 12.24 (24.77) | 7.74 (2.19) | 2.95 (0.12) |
| Lung carcinoma cell type unspecified stage iii | 4 | 24.49 (5.48–109.44) | 24.48 (38.61) | 11.06 (2.48) | 3.47 (0.18) |
| Contraindicated product administered | 4 | 10.5 (3.07–35.86) | 10.49 (21.86) | 7.04 (2.06) | 2.82 (0.09) |
| Cardiovascular insufficiency | 4 | 24.49 (5.48–109.44) | 24.48 (38.61) | 11.06 (2.48) | 3.47 (0.18) |
| Nephrogenic diabetes insipidus | 4 | 14.69 (3.95–54.73) | 14.69 (28.35) | 8.6 (2.31) | 3.11 (0.14) |
| Vanishing bile duct syndrome | 4 | 18.37 (4.59–73.45) | 18.36 (32.83) | 9.68 (2.42) | 3.28 (0.16) |

Supplementary **Table S12A**

Disproportionality analysis of all qualifying preferred terms associated with cisplatin in the age subgroup of patients aged <65 years in the FAERS database

| All qualifying carboplatin-associated preferred terms in patients aged <65 years (n=2,811) | | | | | |
| --- | --- | --- | --- | --- | --- |
| PT | N | ROR (95%CI) | PRR(χ^2^) | EBGM(EBGM05) | IC(IC025) |
| Neutropenia | 69 | 3.79 (2.96–4.86) | 3.73 (129.56) | 3.55 (2.77) | 1.83 (1.41) |
| Acute kidney injury | 48 | 3.41 (2.54–4.57) | 3.37 (75.5) | 3.23 (2.4) | 1.69 (1.2) |
| Pancytopenia | 37 | 3.31 (2.37–4.62) | 3.28 (55.44) | 3.15 (2.25) | 1.65 (1.09) |
| Aortic thrombosis | 28 | 72.41 (41.07–127.67) | 71.7 (836.86) | 31.3 (17.75) | 4.97 (3.25) |
| Septic shock | 17 | 3.53 (2.16–5.78) | 3.52 (28.78) | 3.36 (2.06) | 1.75 (0.86) |
| Oesophagitis | 16 | 4.13 (2.48–6.88) | 4.12 (35.12) | 3.9 (2.34) | 1.96 (1) |
| Renal salt-wasting syndrome | 13 | 702.34 (91.84–5370.89) | 699.1 (647.36) | 50.86 (6.65) | 5.67 (2.38) |
| Posterior reversible encephalopathy syndrome | 13 | 6.56 (3.68–11.68) | 6.53 (54.37) | 5.93 (3.33) | 2.57 (1.31) |
| Nephropathy toxic | 12 | 8.75 (4.75–16.13) | 8.72 (70.62) | 7.64 (4.15) | 2.93 (1.47) |
| Acute myocardial infarction | 12 | 6.89 (3.77–12.58) | 6.87 (53.36) | 6.2 (3.4) | 2.63 (1.29) |
| Neutropenic sepsis | 11 | 5.21 (2.8–9.68) | 5.19 (33.95) | 4.82 (2.59) | 2.27 (0.99) |
| Gastrointestinal toxicity | 11 | 8.24 (4.37–15.57) | 8.22 (60.51) | 7.26 (3.84) | 2.86 (1.35) |
| Tinnitus | 10 | 5.86 (3.05–11.27) | 5.85 (36.25) | 5.37 (2.79) | 2.42 (1.02) |
| Renal infarct | 10 | 35.98 (16.15–80.15) | 35.85 (203.31) | 21.91 (9.83) | 4.45 (1.86) |
| Hypomagnesaemia | 10 | 4.12 (2.16–7.84) | 4.11 (21.84) | 3.88 (2.04) | 1.96 (0.71) |
| Hyperkalaemia | 10 | 4.25 (2.23–8.09) | 4.23 (22.92) | 4 (2.1) | 2 (0.74) |
| Swelling face | 9 | 5 (2.52–9.91) | 4.99 (26.29) | 4.65 (2.35) | 2.22 (0.8) |
| Renal tubular necrosis | 9 | 4.71 (2.38–9.32) | 4.7 (24.12) | 4.4 (2.22) | 2.14 (0.75) |
| Orthostatic hypotension | 9 | 13.48 (6.49–28.02) | 13.44 (82.96) | 10.96 (5.27) | 3.45 (1.43) |
| Capillary leak syndrome | 9 | 25.55 (11.55–56.53) | 25.47 (143.62) | 17.61 (7.96) | 4.14 (1.65) |
| Febrile bone marrow aplasia | 9 | 6.14 (3.08–12.25) | 6.13 (34.68) | 5.6 (2.81) | 2.49 (0.97) |
| Presyncope | 8 | 15.98 (7.25–35.2) | 15.93 (86.4) | 12.52 (5.68) | 3.65 (1.36) |
| Inappropriate antidiuretic hormone secretion | 8 | 6.85 (3.28–14.3) | 6.83 (35.33) | 6.17 (2.95) | 2.63 (0.94) |
| Hypoacusis | 7 | 5.89 (2.7–12.87) | 5.88 (25.58) | 5.4 (2.47) | 2.43 (0.71) |
| Peripheral ischaemia | 7 | 8.98 (4.03–20.01) | 8.96 (42.46) | 7.83 (3.51) | 2.97 (0.96) |
| Bicytopenia | 6 | 10.78 (4.48–25.91) | 10.76 (44.25) | 9.13 (3.8) | 3.19 (0.87) |
| Enteritis | 6 | 5.77 (2.48–13.41) | 5.76 (21.34) | 5.3 (2.28) | 2.41 (0.55) |
| Melanoderma | 6 | 64.67 (19.72–212.02) | 64.53 (170.6) | 29.88 (9.11) | 4.9 (1.15) |
| Ototoxicity | 6 | 17.02 (6.79–42.64) | 16.98 (68.6) | 13.15 (5.25) | 3.72 (1.02) |
| Polyarthritis | 5 | 7.69 (3.01–19.65) | 7.68 (25.44) | 6.85 (2.68) | 2.78 (0.52) |
| Hypovolaemia | 5 | 12.24 (4.63–32.35) | 12.22 (41.99) | 10.14 (3.84) | 3.34 (0.69) |
| Pulmonary nocardiosis | 5 | 89.79 (21.45–375.89) | 89.63 (164.33) | 34.24 (8.18) | 5.1 (0.84) |
| Dilated cardiomyopathy | 5 | 26.93 (9.2–78.85) | 26.89 (83.09) | 18.26 (6.24) | 4.19 (0.83) |
| Guillain-barre syndrome | 5 | 8.16 (3.18–20.92) | 8.15 (27.23) | 7.21 (2.81) | 2.85 (0.54) |
| Splenic infarction | 5 | 22.45 (7.9–63.75) | 22.41 (72.19) | 16.11 (5.67) | 4.01 (0.81) |
| Neutropenic colitis | 5 | 8.98 (3.48–23.15) | 8.96 (30.33) | 7.83 (3.03) | 2.97 (0.58) |
| Hypercreatininaemia | 5 | 11.71 (4.45–30.82) | 11.69 (40.16) | 9.78 (3.72) | 3.29 (0.68) |
| Peripheral artery thrombosis | 5 | 14.18 (5.29–37.99) | 14.15 (48.39) | 11.41 (4.26) | 3.51 (0.73) |
| Hepatitis b | 4 | 10.26 (3.52–29.9) | 10.24 (28.03) | 8.76 (3.01) | 3.13 (0.35) |
| Tonsillitis | 4 | 21.54 (6.75–68.72) | 21.51 (55.88) | 15.65 (4.91) | 3.97 (0.48) |
| Skin hyperpigmentation | 4 | 13.46 (4.5–40.29) | 13.44 (36.86) | 10.96 (3.66) | 3.45 (0.42) |
| Type i hypersensitivity | 4 | 16.57 (5.4–50.85) | 16.55 (44.69) | 12.89 (4.2) | 3.69 (0.45) |
| Arterial thrombosis | 4 | 21.54 (6.75–68.72) | 21.51 (55.88) | 15.65 (4.91) | 3.97 (0.48) |
| Recall phenomenon | 4 | 9.36 (3.24–27.1) | 9.35 (25.42) | 8.12 (2.8) | 3.02 (0.32) |
| Small intestinal obstruction | 4 | 6.33 (2.25–17.86) | 6.33 (16.05) | 5.77 (2.04) | 2.53 (0.17) |
| Cerebral venous thrombosis | 4 | 43.08 (11.56–160.52) | 43.02 (91.22) | 24.35 (6.53) | 4.61 (0.5) |
| Arterial occlusive disease | 4 | 23.93 (7.37–77.76) | 23.9 (60.77) | 16.85 (5.19) | 4.08 (0.49) |
| Salmonellosis | 3 | 53.83 (10.86–266.84) | 53.78 (77.7) | 27.39 (5.53) | 4.78 (0.02) |
| Pulse absent | 3 | 13.46 (3.8–47.72) | 13.44 (27.65) | 10.96 (3.09) | 3.45 (0.02) |
| Myopericarditis | 3 | 40.37 (9.03–180.48) | 40.33 (65.76) | 23.48 (5.25) | 4.55 (0.04) |
| Haemoperitoneum | 3 | 16.15 (4.44–58.71) | 16.13 (32.76) | 12.64 (3.48) | 3.66 (0.03) |
| Tearfulness | 3 | 17.94 (4.86–66.32) | 17.93 (35.96) | 13.69 (3.71) | 3.78 (0.04) |
| Splenic rupture | 3 | 32.3 (7.72–135.22) | 32.27 (56.81) | 20.54 (4.91) | 4.36 (0.05) |
| Ophthalmoplegia | 3 | 20.19 (5.35–76.13) | 20.17 (39.75) | 14.94 (3.96) | 3.9 (0.05) |
| Oesophageal fistula | 3 | 23.07 (5.96–89.26) | 23.05 (44.29) | 16.43 (4.25) | 4.04 (0.05) |
| Ventricular dysfunction | 3 | 40.37 (9.03–180.48) | 40.33 (65.76) | 23.48 (5.25) | 4.55 (0.04) |
| Mallory-weiss syndrome | 3 | 17.94 (4.86–66.32) | 17.93 (35.96) | 13.69 (3.71) | 3.78 (0.04) |
| Klebsiella infection | 3 | 20.19 (5.35–76.13) | 20.17 (39.75) | 14.94 (3.96) | 3.9 (0.05) |
| Cerebellar ataxia | 3 | 23.07 (5.96–89.26) | 23.05 (44.29) | 16.43 (4.25) | 4.04 (0.05) |
| Coronary artery thrombosis | 3 | 32.3 (7.72–135.22) | 32.27 (56.81) | 20.54 (4.91) | 4.36 (0.05) |
| Psychomotor skills impaired | 3 | 40.37 (9.03–180.48) | 40.33 (65.76) | 23.48 (5.25) | 4.55 (0.04) |
| Cd4 lymphocytes decreased | 3 | 53.83 (10.86–266.84) | 53.78 (77.7) | 27.39 (5.53) | 4.78 (0.02) |

Supplementary **Table S12B**

Disproportionality analysis of all qualifying preferred terms associated with cisplatin in the age subgroup of patients aged ≥65 years in the FAERS database

| All qualifying carboplatin-associated preferred terms in patients aged ≥65 years (n=1,817) | | | | | |
| --- | --- | --- | --- | --- | --- |
| PT | N | ROR (95%CI) | PRR(χ^2^) | EBGM(EBGM05) | IC(IC025) |
| Neutropenia | 55 | 4.74 (3.61–6.23) | 4.63 (151.31) | 4.49 (3.41) | 2.17 (1.68) |
| Febrile neutropenia | 53 | 4.36 (3.3–5.75) | 4.26 (128.19) | 4.14 (3.13) | 2.05 (1.56) |
| Anaemia | 51 | 2.81 (2.12–3.72) | 2.76 (56.36) | 2.72 (2.05) | 1.44 (0.98) |
| Thrombocytopenia | 38 | 3.34 (2.41–4.62) | 3.29 (59.19) | 3.22 (2.33) | 1.69 (1.13) |
| Acute kidney injury | 37 | 3.21 (2.31–4.46) | 3.16 (53.59) | 3.1 (2.23) | 1.63 (1.08) |
| Pancytopenia | 32 | 4.05 (2.84–5.78) | 4 (69.75) | 3.89 (2.73) | 1.96 (1.32) |
| Leukopenia | 28 | 5.65 (3.86–8.28) | 5.58 (100.62) | 5.37 (3.66) | 2.42 (1.67) |
| Renal failure | 24 | 4.02 (2.67–6.05) | 3.98 (51.84) | 3.88 (2.57) | 1.95 (1.2) |
| Hyponatraemia | 24 | 4.29 (2.85–6.47) | 4.25 (57.7) | 4.13 (2.74) | 2.05 (1.28) |
| Neutropenic sepsis | 20 | 10.19 (6.44–16.14) | 10.09 (150.55) | 9.35 (5.9) | 3.22 (2.08) |
| Septic shock | 18 | 4.62 (2.88–7.41) | 4.58 (48.54) | 4.44 (2.77) | 2.15 (1.23) |
| Multiple organ dysfunction syndrome | 11 | 5.29 (2.89–9.71) | 5.27 (36.38) | 5.08 (2.77) | 2.34 (1.06) |
| Renal tubular necrosis | 10 | 17.75 (9.1–34.62) | 17.66 (135.97) | 15.41 (7.9) | 3.95 (1.8) |
| Bone marrow failure | 10 | 4.04 (2.15–7.6) | 4.02 (21.96) | 3.92 (2.08) | 1.97 (0.74) |
| Acute myocardial infarction | 10 | 6.2 (3.28–11.75) | 6.18 (41.17) | 5.91 (3.12) | 2.56 (1.13) |
| Nephropathy toxic | 8 | 7.69 (3.75–15.76) | 7.66 (43.43) | 7.24 (3.53) | 2.86 (1.09) |
| Embolism | 7 | 7.86 (3.65–16.93) | 7.83 (39.04) | 7.39 (3.43) | 2.89 (0.97) |
| Hypocalcaemia | 7 | 4.64 (2.18–9.9) | 4.63 (19.13) | 4.48 (2.1) | 2.16 (0.59) |
| Large intestine perforation | 7 | 6.35 (2.96–13.61) | 6.33 (29.76) | 6.05 (2.82) | 2.6 (0.83) |
| Cytomegalovirus oesophagitis | 7 | 158.83 (50.36–500.91) | 158.22 (455.7) | 66.51 (21.09) | 6.06 (1.55) |
| Arterial thrombosis | 6 | 32.39 (13.06–80.36) | 32.29 (141.52) | 25.34 (10.21) | 4.66 (1.27) |
| Coma | 5 | 6.22 (2.53–15.33) | 6.21 (20.73) | 5.94 (2.41) | 2.57 (0.48) |
| Peritonitis | 5 | 5.15 (2.1–12.63) | 5.14 (15.94) | 4.96 (2.02) | 2.31 (0.36) |
| Hypoparathyroidism | 5 | 80.95 (25.67–255.28) | 80.73 (229.67) | 47.51 (15.06) | 5.57 (1) |
| Metabolic acidosis | 5 | 8.85 (3.56–22.02) | 8.83 (32.21) | 8.26 (3.32) | 3.05 (0.66) |
| Gastrointestinal toxicity | 5 | 8.71 (3.51–21.67) | 8.69 (31.62) | 8.14 (3.28) | 3.03 (0.66) |
| Gastroenteritis | 4 | 5.81 (2.12–15.88) | 5.8 (15.1) | 5.56 (2.03) | 2.48 (0.2) |
| Hemiparesis | 4 | 6.97 (2.54–19.15) | 6.95 (19.22) | 6.61 (2.41) | 2.72 (0.29) |
| Ileus paralytic | 4 | 8.88 (3.21–24.6) | 8.86 (25.88) | 8.29 (2.99) | 3.05 (0.4) |
| Intestinal ischaemia | 4 | 11.61 (4.15–32.54) | 11.59 (35.12) | 10.61 (3.79) | 3.41 (0.49) |
| Circulatory collapse | 4 | 6.86 (2.5–18.85) | 6.85 (18.85) | 6.52 (2.37) | 2.7 (0.29) |
| Peripheral artery thrombosis | 4 | 34.85 (11.35–106.98) | 34.77 (100.35) | 26.83 (8.74) | 4.75 (0.65) |
| Empyema | 3 | 15.44 (4.62–51.62) | 15.41 (35.58) | 13.68 (4.09) | 3.77 (0.15) |
| Fibrosis | 3 | 48.51 (12.54–187.76) | 48.44 (97.56) | 34.2 (8.84) | 5.1 (0.18) |
| Kaposi's sarcoma | 3 | 84.9 (18.99–379.62) | 84.76 (141.9) | 48.86 (10.93) | 5.61 (0.13) |
| Ischaemic cardiomyopathy | 3 | 67.92 (16.22–284.42) | 67.81 (123.43) | 42.76 (10.21) | 5.42 (0.16) |
| Coronary artery occlusion | 3 | 28.3 (7.98–100.37) | 28.25 (63.1) | 22.8 (6.43) | 4.51 (0.2) |
| Cerebral venous thrombosis | 3 | 169.8 (28.36–1016.85) | 169.52 (201.04) | 68.41 (11.42) | 6.1 (0.06) |
| Renal salt-wasting syndrome | 3 | 113.2 (22.83–561.25) | 113.02 (166.54) | 57.01 (11.5) | 5.83 (0.1) |
| Carotid artery thrombosis | 3 | 113.2 (22.83–561.25) | 113.02 (166.54) | 57.01 (11.5) | 5.83 (0.1) |

Supplementary **Table S12C**

Disproportionality analysis of all qualifying preferred terms associated with cisplatin in the sex subgroup of male patients in the FAERS database

| All qualifying carboplatin-associated preferred terms in male patients (n=3,022) | | | | | |
| --- | --- | --- | --- | --- | --- |
| PT | N | ROR (95%CI) | PRR(χ^2^) | EBGM(EBGM05) | IC(IC025) |
| Neutropenia | 76 | 3.5 (2.78–4.42) | 3.44 (126.64) | 3.33 (2.64) | 1.74 (1.35) |
| Acute kidney injury | 66 | 3.64 (2.84–4.67) | 3.58 (117.89) | 3.46 (2.7) | 1.79 (1.37) |
| Febrile neutropenia | 63 | 3.11 (2.41–4.01) | 3.07 (84.89) | 2.99 (2.31) | 1.58 (1.16) |
| Pancytopenia | 48 | 3.49 (2.61–4.67) | 3.45 (80.08) | 3.34 (2.49) | 1.74 (1.25) |
| Leukopenia | 41 | 4.36 (3.17–5.98) | 4.31 (98.83) | 4.13 (3.01) | 2.05 (1.48) |
| Pulmonary embolism | 39 | 3.05 (2.21–4.21) | 3.02 (50.9) | 2.94 (2.13) | 1.56 (1.02) |
| Renal failure | 35 | 3.35 (2.38–4.71) | 3.33 (54.65) | 3.23 (2.29) | 1.69 (1.11) |
| Septic shock | 33 | 4.44 (3.12–6.32) | 4.4 (82.12) | 4.21 (2.96) | 2.07 (1.43) |
| Neutropenic sepsis | 22 | 7.54 (4.86–11.71) | 7.49 (112.52) | 6.9 (4.44) | 2.79 (1.82) |
| Nephropathy toxic | 20 | 11.9 (7.41–19.1) | 11.83 (170.99) | 10.33 (6.44) | 3.37 (2.16) |
| Multiple organ dysfunction syndrome | 18 | 4.79 (2.97–7.73) | 4.77 (50.43) | 4.54 (2.82) | 2.18 (1.25) |
| Aortic thrombosis | 16 | 118.89 (53.91–262.2) | 118.26 (715.59) | 46.1 (20.9) | 5.53 (2.75) |
| Acute myocardial infarction | 13 | 4.97 (2.83–8.72) | 4.95 (38.47) | 4.7 (2.68) | 2.23 (1.09) |
| Hypomagnesaemia | 12 | 5.2 (2.89–9.35) | 5.19 (37.93) | 4.91 (2.73) | 2.3 (1.08) |
| Orthostatic hypotension | 11 | 10.2 (5.42–19.17) | 10.16 (79.92) | 9.06 (4.82) | 3.18 (1.54) |
| Inappropriate antidiuretic hormone secretion | 10 | 5.88 (3.09–11.21) | 5.87 (37.42) | 5.51 (2.89) | 2.46 (1.05) |
| Enteritis | 9 | 5.25 (2.67–10.33) | 5.24 (28.84) | 4.96 (2.52) | 2.31 (0.87) |
| Hypocalcaemia | 9 | 4.25 (2.17–8.32) | 4.24 (21.07) | 4.06 (2.07) | 2.02 (0.69) |
| Arterial thrombosis | 9 | 31.77 (14.54–69.42) | 31.68 (187.19) | 22.47 (10.28) | 4.49 (1.77) |
| Renal salt-wasting syndrome | 9 | 667.21 (84.5–5268.17) | 665.23 (596.93) | 67.42 (8.54) | 6.08 (1.86) |
| Tinnitus | 8 | 7.41 (3.58–15.34) | 7.39 (40.21) | 6.81 (3.29) | 2.77 (1.03) |
| Bicytopenia | 8 | 10.58 (5.04–22.22) | 10.56 (60.59) | 9.36 (4.46) | 3.23 (1.24) |
| Peritonitis | 8 | 4.46 (2.18–9.1) | 4.45 (20.17) | 4.25 (2.08) | 2.09 (0.64) |
| Febrile bone marrow aplasia | 8 | 4.36 (2.13–8.9) | 4.35 (19.49) | 4.16 (2.04) | 2.06 (0.62) |
| Deafness | 7 | 4.98 (2.32–10.72) | 4.97 (20.84) | 4.72 (2.2) | 2.24 (0.62) |
| Renal infarct | 7 | 34.57 (14.09–84.85) | 34.49 (155.23) | 23.84 (9.71) | 4.58 (1.43) |
| Renal tubular necrosis | 7 | 5.24 (2.43–11.28) | 5.23 (22.35) | 4.95 (2.3) | 2.31 (0.66) |
| Splenic infarction | 7 | 37.04 (14.94–91.84) | 36.96 (163.27) | 24.97 (10.07) | 4.64 (1.44) |
| Metabolic acidosis | 7 | 8.64 (3.95–18.92) | 8.62 (42.26) | 7.83 (3.57) | 2.97 (0.99) |
| Hepatitis b | 6 | 15.32 (6.36–36.93) | 15.29 (66.42) | 12.84 (5.33) | 3.68 (1.05) |
| Ileus paralytic | 6 | 7.17 (3.1–16.58) | 7.15 (28.96) | 6.61 (2.86) | 2.72 (0.72) |
| Cytomegalovirus oesophagitis | 6 | 88.87 (27.11–291.36) | 88.7 (236.49) | 40.86 (12.46) | 5.35 (1.22) |
| Intestinal ischaemia | 5 | 8.22 (3.26–20.73) | 8.21 (28.51) | 7.49 (2.97) | 2.91 (0.59) |
| Hypoparathyroidism | 5 | 41.13 (13.78–122.8) | 41.06 (125.65) | 26.75 (8.96) | 4.74 (0.93) |
| Type i hypersensitivity | 5 | 37.02 (12.64–108.36) | 36.96 (116.62) | 24.97 (8.53) | 4.64 (0.92) |
| Neutropenic colitis | 5 | 7.12 (2.84–17.83) | 7.11 (23.94) | 6.57 (2.62) | 2.72 (0.52) |
| Idiopathic pulmonary fibrosis | 5 | 9.25 (3.65–23.46) | 9.24 (32.66) | 8.32 (3.28) | 3.06 (0.64) |
| Arterial occlusive disease | 5 | 26.44 (9.52–73.45) | 26.4 (90.03) | 19.71 (7.1) | 4.3 (0.9) |
| Empyema | 4 | 6.73 (2.42–18.73) | 6.72 (17.85) | 6.24 (2.24) | 2.64 (0.24) |
| Photophobia | 4 | 8.22 (2.92–23.11) | 8.21 (22.81) | 7.49 (2.66) | 2.91 (0.32) |
| Hypovolaemia | 4 | 7.4 (2.65–20.7) | 7.39 (20.1) | 6.81 (2.44) | 2.77 (0.28) |
| Colitis ischaemic | 4 | 6.17 (2.22–17.11) | 6.16 (15.96) | 5.76 (2.08) | 2.53 (0.2) |
| Diastolic dysfunction | 4 | 26.91 (8.56–84.57) | 26.88 (73.09) | 19.98 (6.36) | 4.32 (0.56) |
| Coronary artery thrombosis | 4 | 22.77 (7.42–69.88) | 22.74 (63.59) | 17.63 (5.74) | 4.14 (0.55) |
| Diabetes mellitus inadequate control | 4 | 8.22 (2.92–23.11) | 8.21 (22.81) | 7.49 (2.66) | 2.91 (0.32) |
| Cerebral venous thrombosis | 4 | 59.21 (15.89–220.6) | 59.13 (127) | 33.3 (8.94) | 5.06 (0.56) |
| Fibrosis | 3 | 20.18 (5.63–72.36) | 20.16 (42.92) | 16.05 (4.48) | 4 (0.11) |
| Melanoderma | 3 | 73.99 (14.93–366.73) | 73.91 (107.89) | 37.46 (7.56) | 5.23 (0.06) |
| Ototoxicity | 3 | 11.1 (3.3–37.36) | 11.09 (23.94) | 9.77 (2.9) | 3.29 (0.04) |
| Ophthalmoplegia | 3 | 17.07 (4.86–59.94) | 17.06 (36.85) | 14.05 (4) | 3.81 (0.1) |
| Tonsillitis | 3 | 36.99 (9.25–147.99) | 36.96 (69.97) | 24.97 (6.24) | 4.64 (0.11) |
| Kaposi's sarcoma | 3 | 55.49 (12.41–248.04) | 55.44 (91.64) | 32.11 (7.18) | 5 (0.09) |
| Mallory-weiss syndrome | 3 | 20.18 (5.63–72.36) | 20.16 (42.92) | 16.05 (4.48) | 4 (0.11) |
| Klebsiella infection | 3 | 22.2 (6.11–80.69) | 22.17 (46.66) | 17.29 (4.76) | 4.11 (0.12) |
| Cerebellar ataxia | 3 | 17.07 (4.86–59.94) | 17.06 (36.85) | 14.05 (4) | 3.81 (0.1) |
| Progressive multifocal leukoencephalopathy | 3 | 13.06 (3.82–44.57) | 13.04 (28.36) | 11.24 (3.29) | 3.49 (0.07) |
| Peripheral artery thrombosis | 3 | 10.09 (3.02–33.72) | 10.08 (21.59) | 8.99 (2.69) | 3.17 (0.02) |
| Psychomotor skills impaired | 3 | 55.49 (12.41–248.04) | 55.44 (91.64) | 32.11 (7.18) | 5 (0.09) |
| Cd4 lymphocytes decreased | 3 | 73.99 (14.93–366.73) | 73.91 (107.89) | 37.46 (7.56) | 5.23 (0.06) |

Supplementary **Table S12D**

Disproportionality analysis of all qualifying preferred terms associated with cisplatin in the sex subgroup of female patients in the FAERS database

| All qualifying cisplatin-associated preferred terms in female patients (n=1,892) | | | | | |
| --- | --- | --- | --- | --- | --- |
| PT | N | ROR (95%CI) | PRR(χ^2^) | EBGM(EBGM05) | IC(IC025) |
| Neutropenia | 57 | 7.08 (5.4–9.28) | 6.89 (272.15) | 6.56 (5) | 2.71 (2.18) |
| Febrile neutropenia | 30 | 4.34 (3.01–6.27) | 4.29 (73.16) | 4.17 (2.89) | 2.06 (1.39) |
| Acute kidney injury | 30 | 4.1 (2.84–5.92) | 4.05 (66.79) | 3.94 (2.73) | 1.98 (1.32) |
| Sepsis | 19 | 3.8 (2.4–6.02) | 3.77 (37.59) | 3.68 (2.33) | 1.88 (1.04) |
| Pancytopenia | 18 | 3.41 (2.13–5.46) | 3.39 (29.48) | 3.32 (2.07) | 1.73 (0.89) |
| Leukopenia | 16 | 4.51 (2.73–7.44) | 4.48 (41.67) | 4.35 (2.63) | 2.12 (1.14) |
| Aortic thrombosis | 13 | 64.98 (32.87–128.46) | 64.54 (519.63) | 41.59 (21.04) | 5.38 (2.5) |
| Gastrointestinal toxicity | 13 | 18.91 (10.5–34.06) | 18.79 (188.1) | 16.28 (9.04) | 4.02 (2.12) |
| Renal tubular necrosis | 12 | 17.9 (9.73–32.95) | 17.79 (164.63) | 15.53 (8.44) | 3.96 (2.01) |
| Lung adenocarcinoma | 11 | 4.52 (2.47–8.27) | 4.5 (28.87) | 4.37 (2.39) | 2.13 (0.91) |
| Oesophagitis | 9 | 5.35 (2.74–10.45) | 5.32 (30.23) | 5.13 (2.63) | 2.36 (0.92) |
| Swelling face | 9 | 5.73 (2.93–11.21) | 5.71 (33.32) | 5.48 (2.8) | 2.46 (0.98) |
| Bone marrow failure | 9 | 5.55 (2.84–10.84) | 5.52 (31.84) | 5.32 (2.72) | 2.41 (0.95) |
| Capillary leak syndrome | 9 | 41.3 (19.25–88.59) | 41.11 (258.99) | 30.49 (14.21) | 4.93 (1.9) |
| Acute myocardial infarction | 9 | 10.86 (5.48–21.55) | 10.82 (73.28) | 9.97 (5.02) | 3.32 (1.43) |
| Posterior reversible encephalopathy syndrome | 9 | 9.05 (4.59–17.87) | 9.01 (59.47) | 8.43 (4.27) | 3.08 (1.32) |
| Embolism | 8 | 7.9 (3.86–16.2) | 7.87 (44.94) | 7.43 (3.62) | 2.89 (1.11) |
| Ischaemic stroke | 7 | 6.91 (3.22–14.84) | 6.89 (33.26) | 6.56 (3.05) | 2.71 (0.89) |
| Haematotoxicity | 7 | 7.71 (3.58–16.6) | 7.69 (38.16) | 7.26 (3.37) | 2.86 (0.96) |
| Neutropenic sepsis | 7 | 5.9 (2.75–12.62) | 5.88 (26.96) | 5.64 (2.63) | 2.5 (0.78) |
| Peripheral artery thrombosis | 7 | 57.3 (23.1–142.13) | 57.09 (257.2) | 38.39 (15.48) | 5.26 (1.55) |
| Renal salt-wasting syndrome | 7 | 160.45 (50.88–505.98) | 159.86 (460.45) | 67.19 (21.31) | 6.07 (1.55) |
| Tinnitus | 6 | 5.97 (2.63–13.59) | 5.96 (23.54) | 5.71 (2.51) | 2.51 (0.64) |
| Hemiparesis | 6 | 7.99 (3.49–18.3) | 7.97 (34.18) | 7.51 (3.28) | 2.91 (0.82) |
| Polyarthritis | 5 | 15.06 (5.92–38.31) | 15.02 (57.85) | 13.39 (5.27) | 3.74 (0.86) |
| Polyneuropathy | 5 | 5.15 (2.1–12.64) | 5.14 (15.98) | 4.96 (2.02) | 2.31 (0.36) |
| Agitation | 5 | 9.87 (3.95–24.63) | 9.84 (36.58) | 9.14 (3.66) | 3.19 (0.71) |
| Neurotoxicity | 5 | 10.22 (4.09–25.54) | 10.19 (38.08) | 9.44 (3.78) | 3.24 (0.73) |
| Depressed mood | 5 | 7.06 (2.86–17.45) | 7.05 (24.45) | 6.7 (2.71) | 2.74 (0.55) |
| Melanoderma | 5 | 71.55 (23.39–218.92) | 71.36 (213.48) | 44.3 (14.48) | 5.47 (1.01) |
| Peripheral ischaemia | 5 | 26.02 (9.84–68.78) | 25.95 (97.75) | 21.33 (8.07) | 4.41 (0.97) |
| Lower respiratory tract infection | 5 | 5.25 (2.14–12.88) | 5.24 (16.4) | 5.05 (2.06) | 2.34 (0.38) |
| Cyanosis | 4 | 8.8 (3.18–24.35) | 8.78 (25.62) | 8.23 (2.97) | 3.04 (0.39) |
| Ataxia | 4 | 6.93 (2.52–19.04) | 6.92 (19.11) | 6.58 (2.4) | 2.72 (0.29) |
| Renal infarct | 4 | 35.21 (11.47–108.07) | 35.13 (101.44) | 27.1 (8.83) | 4.76 (0.65) |
| Presyncope | 4 | 16.34 (5.73–46.64) | 16.31 (50.31) | 14.4 (5.05) | 3.85 (0.57) |
| Metastases to adrenals | 4 | 9.15 (3.3–25.37) | 9.13 (26.84) | 8.53 (3.08) | 3.09 (0.41) |
| Recall phenomenon | 4 | 25.43 (8.6–75.2) | 25.37 (76.63) | 20.94 (7.08) | 4.39 (0.63) |
| Small intestinal obstruction | 4 | 12.04 (4.29–33.78) | 12.02 (36.57) | 10.97 (3.91) | 3.46 (0.5) |
| Arterial occlusive disease | 4 | 32.69 (10.75–99.41) | 32.62 (95.38) | 25.6 (8.42) | 4.68 (0.65) |
| Salmonellosis | 3 | 171.54 (28.65–1027.25) | 171.27 (203.14) | 69.11 (11.54) | 6.11 (0.06) |
| Myopericarditis | 3 | 85.77 (19.18–383.5) | 85.64 (143.4) | 49.36 (11.04) | 5.63 (0.13) |
| Formication | 3 | 57.18 (14.29–228.81) | 57.09 (110.22) | 38.39 (9.59) | 5.26 (0.17) |
| Haemoperitoneum | 3 | 68.62 (16.39–287.33) | 68.51 (124.74) | 43.19 (10.31) | 5.43 (0.16) |
| Tearfulness | 3 | 34.31 (9.43–124.76) | 34.25 (74.51) | 26.58 (7.31) | 4.73 (0.2) |
| Splenic rupture | 3 | 114.36 (23.07–566.99) | 114.18 (168.29) | 57.59 (11.62) | 5.85 (0.11) |
| Ototoxicity | 3 | 42.88 (11.37–161.78) | 42.82 (89.11) | 31.41 (8.33) | 4.97 (0.19) |
| Pulmonary nocardiosis | 3 | 171.54 (28.65–1027.25) | 171.27 (203.14) | 69.11 (11.54) | 6.11 (0.06) |
| Dilated cardiomyopathy | 3 | 21.44 (6.24–73.65) | 21.41 (49.16) | 18.19 (5.29) | 4.18 (0.19) |
| Radiation oesophagitis | 3 | 13.72 (4.14–45.49) | 13.7 (31.54) | 12.34 (3.72) | 3.63 (0.14) |
| Skin hyperpigmentation | 3 | 9.8 (3.01–31.9) | 9.79 (21.8) | 9.09 (2.79) | 3.18 (0.06) |
| Systemic scleroderma | 3 | 57.18 (14.29–228.81) | 57.09 (110.22) | 38.39 (9.59) | 5.26 (0.17) |
| Oesophageal ulcer | 3 | 8.17 (2.53–26.37) | 8.16 (17.58) | 7.68 (2.38) | 2.94 (0.01) |
| Henoch-schonlein purpura | 3 | 10.09 (3.1–32.88) | 10.07 (22.54) | 9.34 (2.87) | 3.22 (0.07) |
| Cytomegalovirus oesophagitis | 3 | 114.36 (23.07–566.99) | 114.18 (168.29) | 57.59 (11.62) | 5.85 (0.11) |
| Coronary artery occlusion | 3 | 42.88 (11.37–161.78) | 42.82 (89.11) | 31.41 (8.33) | 4.97 (0.19) |
| Contraindicated product administered | 3 | 42.88 (11.37–161.78) | 42.82 (89.11) | 31.41 (8.33) | 4.97 (0.19) |
| Cerebral venous thrombosis | 3 | 114.36 (23.07–566.99) | 114.18 (168.29) | 57.59 (11.62) | 5.85 (0.11) |
| Carotid artery thrombosis | 3 | 114.36 (23.07–566.99) | 114.18 (168.29) | 57.59 (11.62) | 5.85 (0.11) |
| Drug monitoring procedure not performed | 3 | 171.54 (28.65–1027.25) | 171.27 (203.14) | 69.11 (11.54) | 6.11 (0.06) |
| Neutrophil count increased | 3 | 10.72 (3.28–35.04) | 10.7 (24.14) | 9.87 (3.02) | 3.3 (0.09) |
| Renal function test abnormal | 3 | 14.29 (4.3–47.51) | 14.27 (32.92) | 12.8 (3.85) | 3.68 (0.14) |
